# Author Correction: GWAS for male-pattern baldness identifies 71 susceptibility loci explaining 38% of the risk

**DOI:** 10.1038/s41467-018-04857-7

**Published:** 2018-06-29

**Authors:** Nicola Pirastu, Peter K. Joshi, Paul S. de Vries, Marilyn C. Cornelis, Paul M. McKeigue, NaNa Keum, Nora Franceschini, Marco Colombo, Edward L. Giovannucci, Athina Spiliopoulou, Lude Franke, Kari E. North, Peter Kraft, Alanna C. Morrison, Tõnu Esko, James F. Wilson

**Affiliations:** 1 Centre for Global Health Research, Usher Institute of Population Health Sciences and Informatics, University of Edinburgh, Teviot Place, Edinburgh, EH8 9AG Scotland; 2 Human Genetics Center, Department of Epidemiology, Human Genetics and Environmental Sciences, School of Public Health, The University of Texas Health Science Center at Houston, Houston, TX 77030 USA; 3 Department of Preventive Medicine, Northwestern University Feinberg School of Medicine, Chicago, IL 60611 USA; 4 Centre for Population Health Sciences, Usher Institute of Population Health Sciences and Informatics, University of Edinburgh, Teviot Place, Edinburgh, EH8 9AG Scotland; 5 Department of Food Science and Biotechnology, Dongguk University, Goyang, South Korea; 6 Department of Nutrition, Harvard T. H. Chan School of Public Health, Boston, MA 02115 USA; 7 Department of Epidemiology and Carolina Center for Genome Sciences, University of North Carolina, Chapel Hill, NC 27599 USA; 8 Department of Epidemiology, Harvard T. H. Chan School of Public Health, Boston, MA 0211 USA; 9 Channing Division of Network Medicine, Department of Medicine, Brigham and Women’s Hospital and Harvard Medical School, Boston, MA 02115 USA; 10 Pharmatics Ltd, Edinburgh, EH16 4UX Scotland; 11 Department of Genetics, University Medical Center, 9713 GZ Gröningen, The Netherlands; 12 Program in Genetic Epidemiology and Statistical Genetics, Harvard T. H. Chan School of Public Health, Boston, MA 02115 USA; 13 Estonian Genome Center, University of Tartu, Tartu, 51010 Estonia; 14 Broad Institute of Harvard and MIT, Cambridge, MA 02142 USA; 15 MRC Human Genetics Unit, Institute of Genetics and Molecular Medicine, University of Edinburgh, Western General Hospital, Crewe Road, Edinburgh, EH4 2XU Scotland

Correction to: *Nature Communications* 10.1038/s41467-017-01490-8, published online 17 November 2017.

We have been alerted^1^ that in our recent Article^2^ the calculations used to transform the heritability from the observed scale to the liability scale did not take into account the individuals in category 2 of the baldness scale, who were removed in our original analysis. This led to an overestimation of the heritability on the liability scale, which should have been 0.62 instead of 0.94. Moreover, in the Title and in the Abstract, we report that we can explain 38% of the risk, while in fact that is the proportion of heritability explained by the loci we discovered. These errors do not substantially change the paper or its conclusions apart from the statement MBP is therefore probably one of the most heritable complex traits. Genome-wide significant associations and pathway analyses are not affected in any way and male-pattern baldness remains less genetically complex than other complex traits. We wish to thank Yap et al. for bringing this to our attention.
