# Supplementary material for: GWAS for male-pattern baldness identifies 71 susceptibility loci explaining 38% of the risk
Source: Nat Commun. 2017 Nov 17;8:1584. doi: 10.1038/s41467-017-01490-8 (PMC5691155; doi:10.1038/s41467-017-01490-8)
Supplement: Supplementary file 1 — Supplementary Information [file 41467_2017_1490_MOESM1_ESM.pdf]

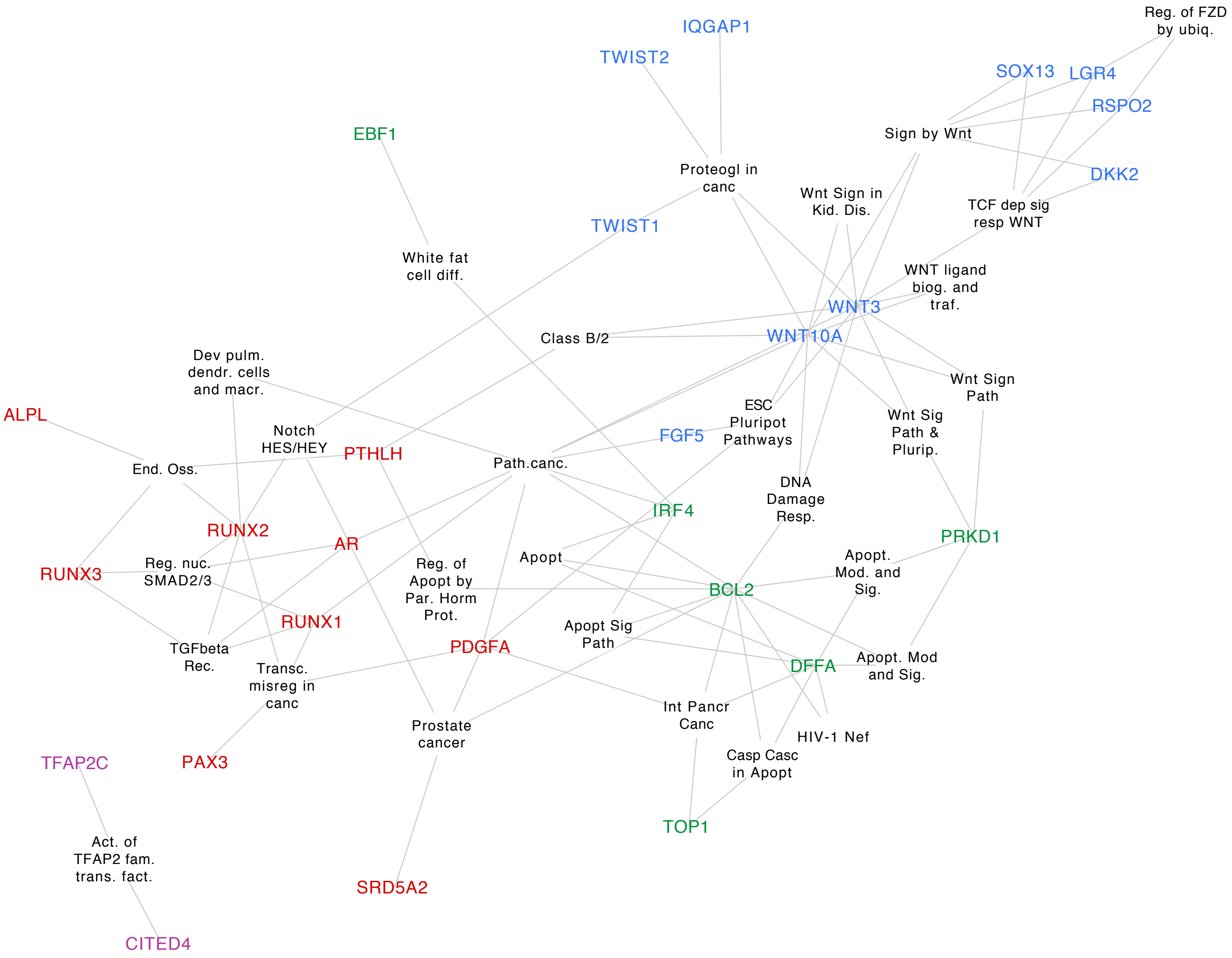

Supplementary Figure 1: Genes annotated to be in known pathways. The colours represent the different communities estimated with Luvain's method. The genes divide into 4 groups, one which includes genes found in Wnt-related pathways (in blue), the second in apoptosis-related pathways (in green) and the third one is more heterogeneous and includes the AR pathway and TGF-beta (in red). The two violet genes represent a single pathway.

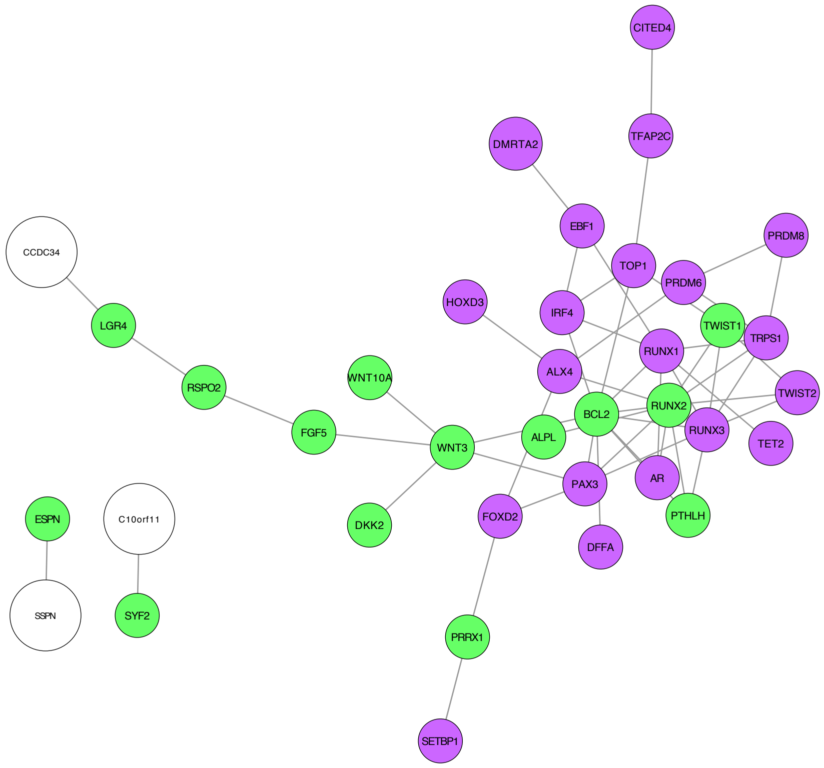

Supplementary Figure 2:The interaction network formed by the genes prioritised in our analyses. Only directly interacting proteins have been included. Colours reflect the communities estimated from the GO-term annotation: genes related to known signalling pathways are in green, while the genes related to the other pathways are in purple. These results suggest a considerable cross-talk between different signalling pathways through a shared trunk which is then transduced to the effector pathways.

Supplementary Figure 3: Regional association plots for the 71 loci associated to Male Pattern Baldness. Distinctly associated SNPs in each locus are represented in blue

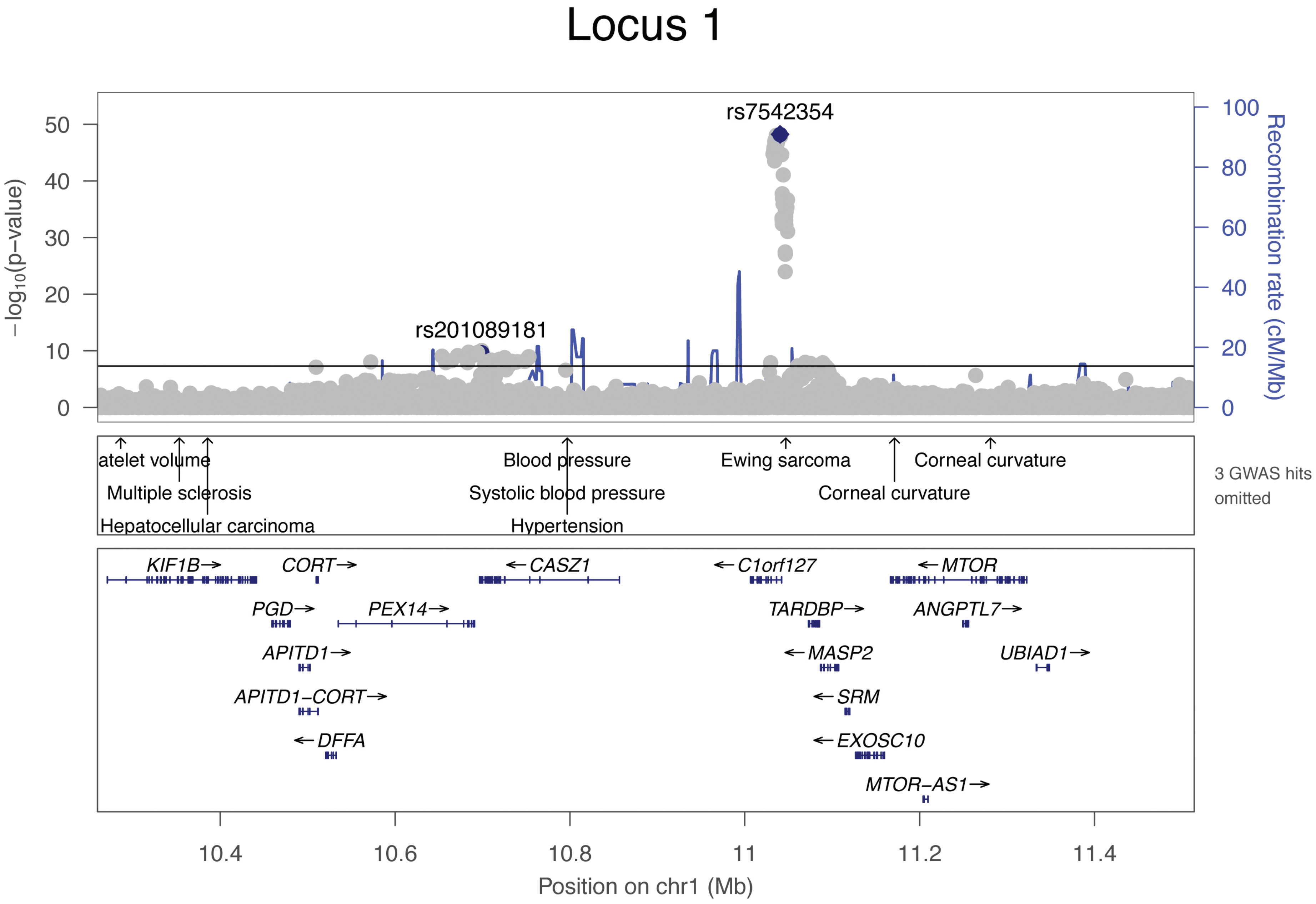

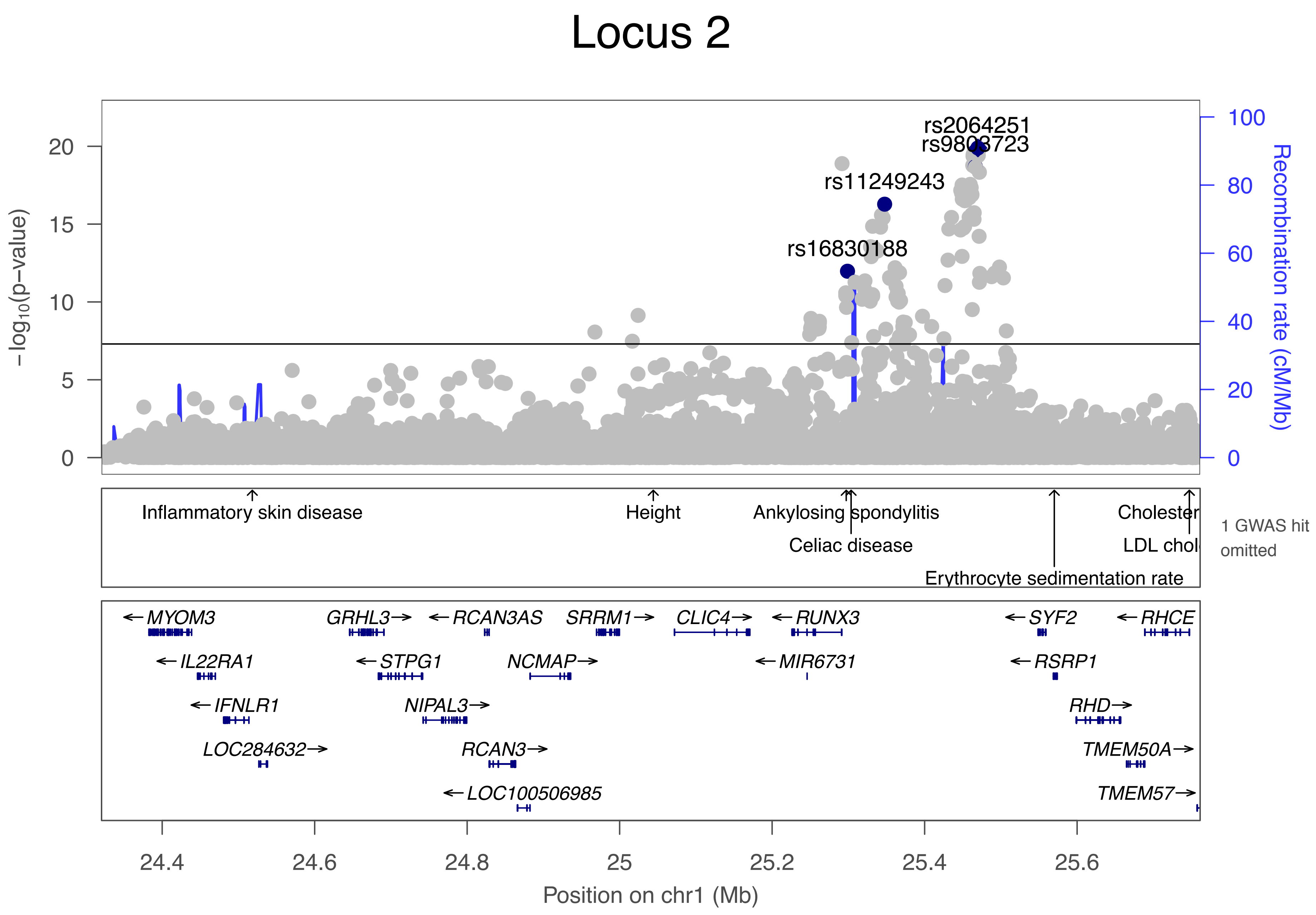

Locus 2
rs2064251
rs9803723
rs11249243
rs16830188
−log10(p−value)
Recombination rate (cM/Mb)
Inflammatory skin disease
Height
Ankylosing spondylitis
Celiac disease
Erythrocyte sedimentation rate
Cholester
LDL chol
1 GWAS hit omitted
MYOM3
IL22RA1
IFNLR1
LOC284632
GRHL3
STPG1
NIPAL3
RCAN3AS
RCAN3
LOC100506985
NCMAP
SRRM1
CLIC4
RUNX3
MIR6731
SYF2
RSRP1
RHD
RHCE
TMEM50A
TMEM57
Position on chr1 (Mb)

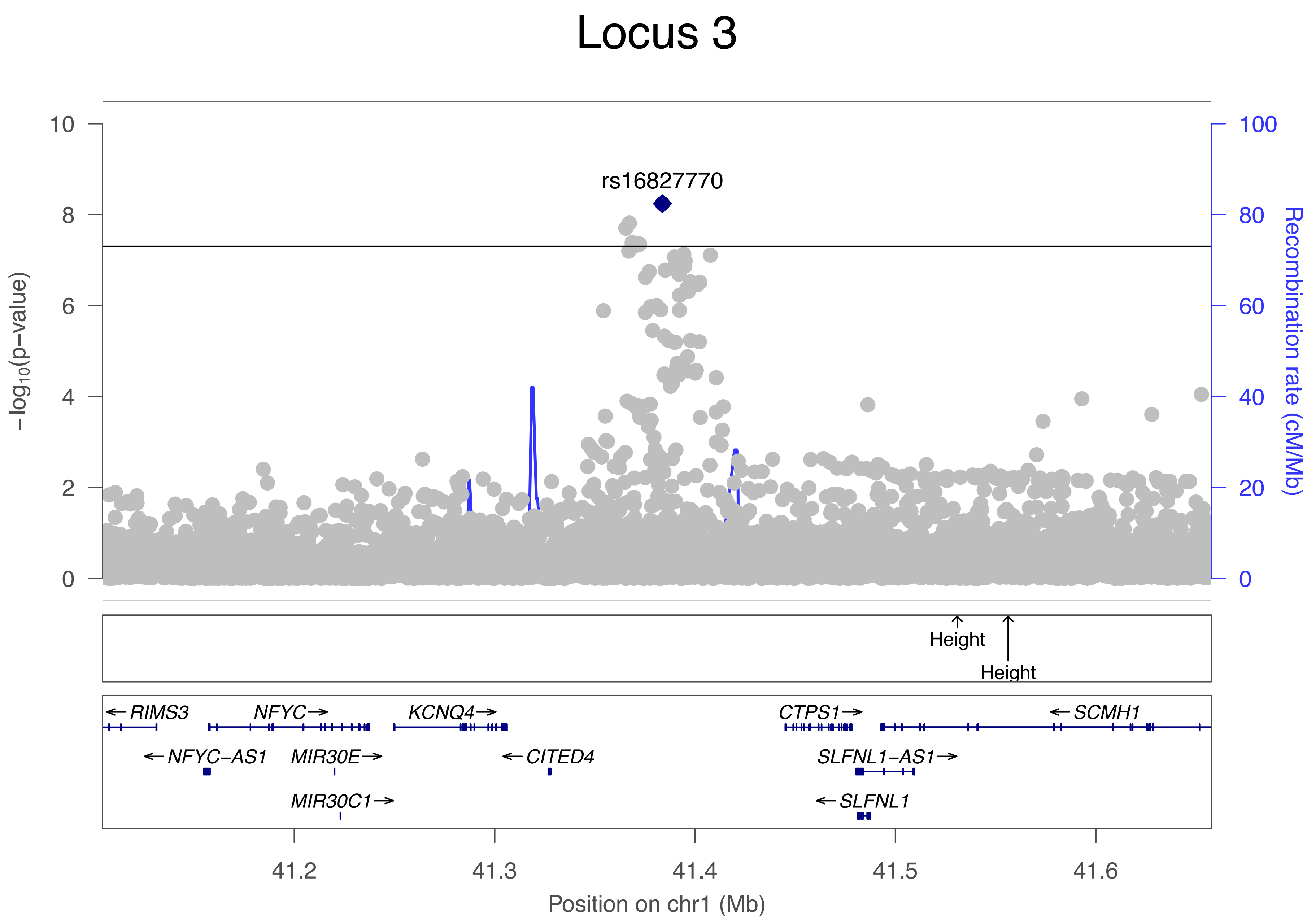
Locus 3
rs16827770
−log10(p−value)
Recombination rate (cM/Mb)
10
8
6
4
2
0
100
80
60
40
20
0
Height
Height
← RIMS3
NFYC→
KCNQ4→
CTPS1→
← SCMH1
← NFYC−AS1
MIR30E→
← CITED4
SLFNL1−AS1→
MIR30C1→
← SLFNL1
41.2
41.3
41.4
41.5
41.6
Position on chr1 (Mb)

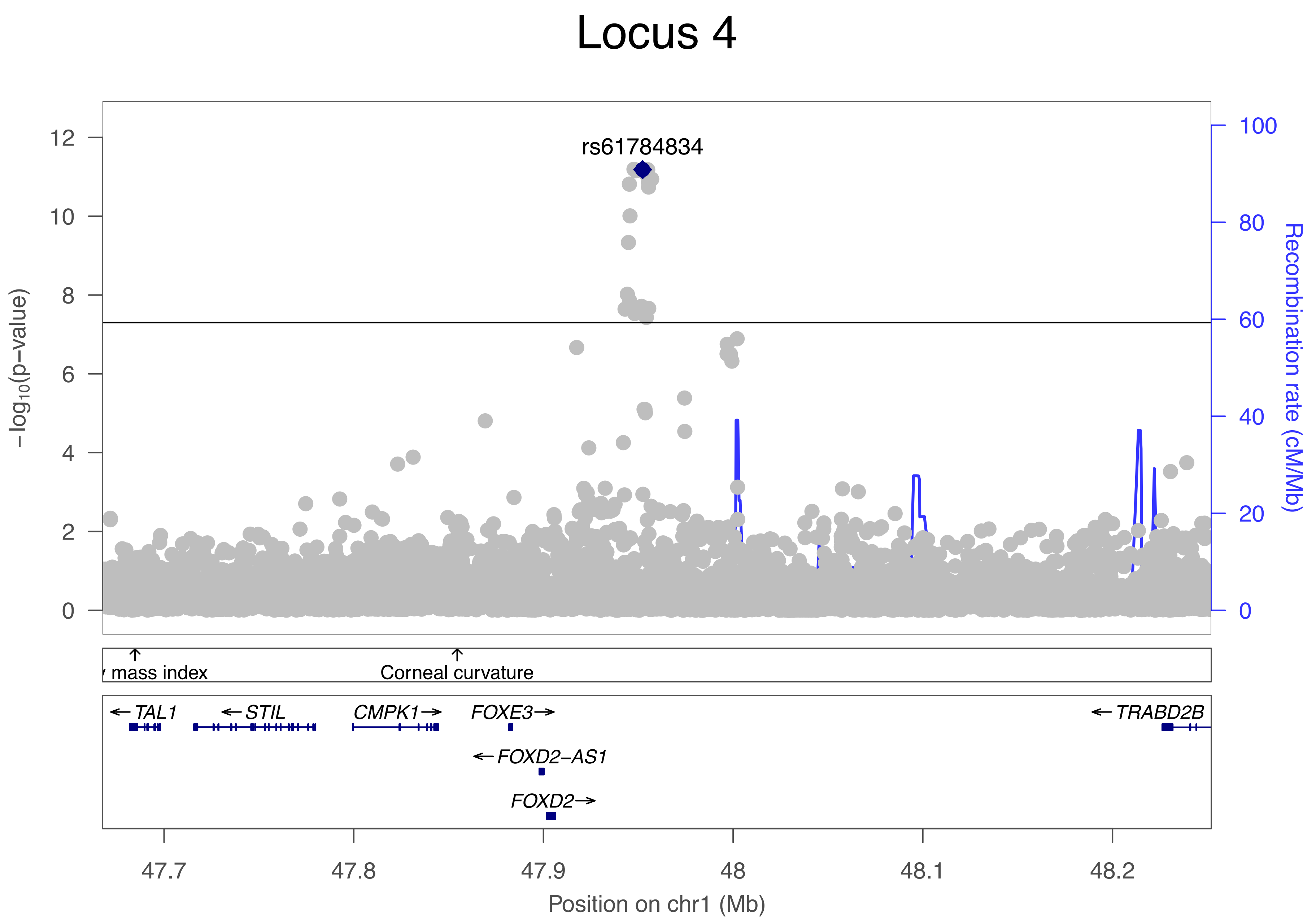

Locus 4
rs61784834
−log10(p−value)
Recombination rate (cM/Mb)
12
10
8
6
4
2
0
100
80
60
40
20
0
mass index
Corneal curvature
TAL1
STIL
CMPK1
FOXE3
TRABD2B
FOXD2−AS1
FOXD2
47.7
47.8
47.9
48
48.1
48.2
Position on chr1 (Mb)

Locus 5

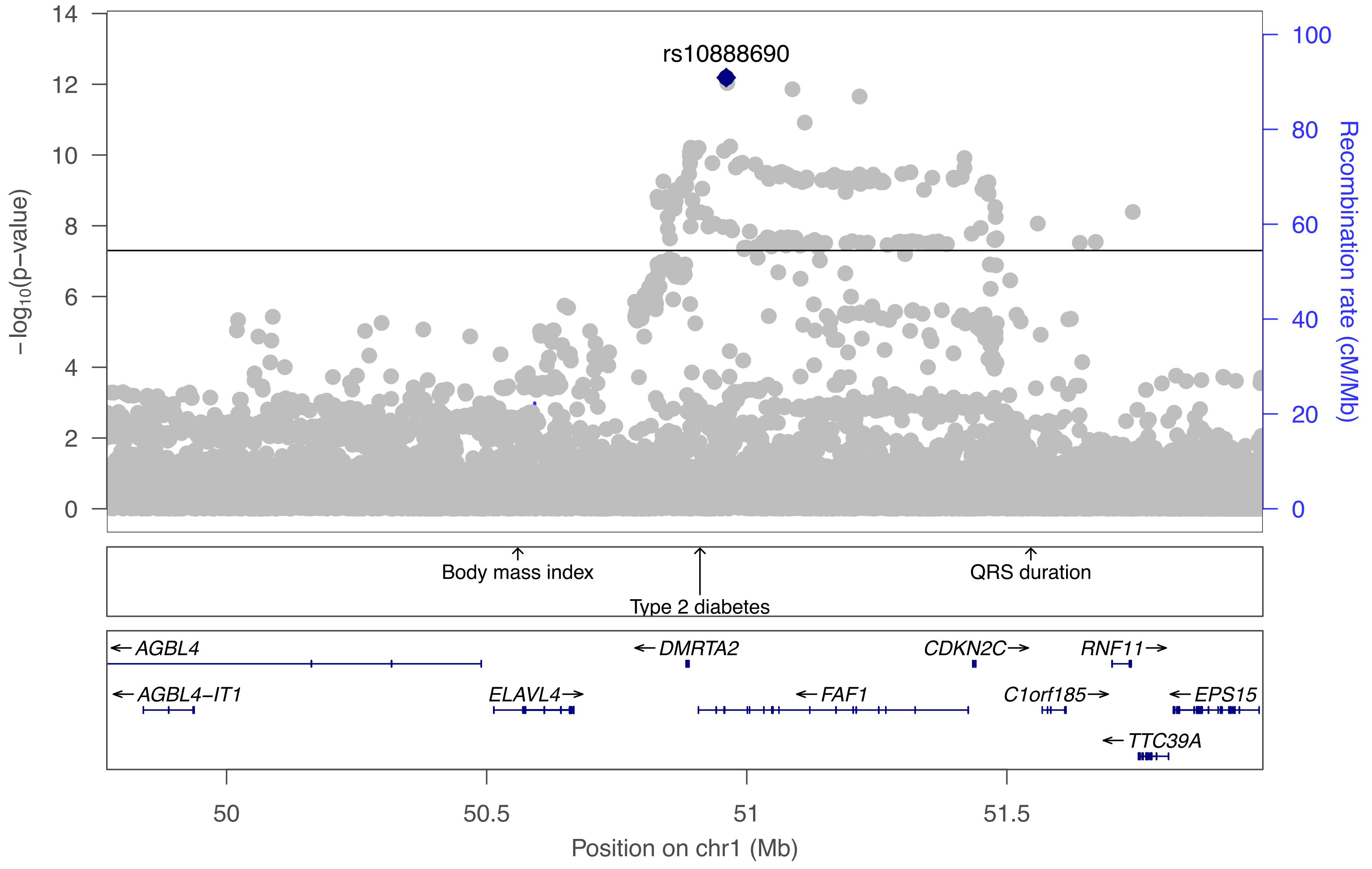
rs10888690
−log10(p−value)
Recombination rate (cM/Mb)
14
12
10
8
6
4
2
0
100
80
60
40
20
0
Body mass index
Type 2 diabetes
QRS duration
AGBL4
DMRTA2
CDKN2C
RNF11
AGBL4−IT1
ELAVL4
FAF1
C1orf185
EPS15
TTC39A
50
50.5
51
51.5
Position on chr1 (Mb)

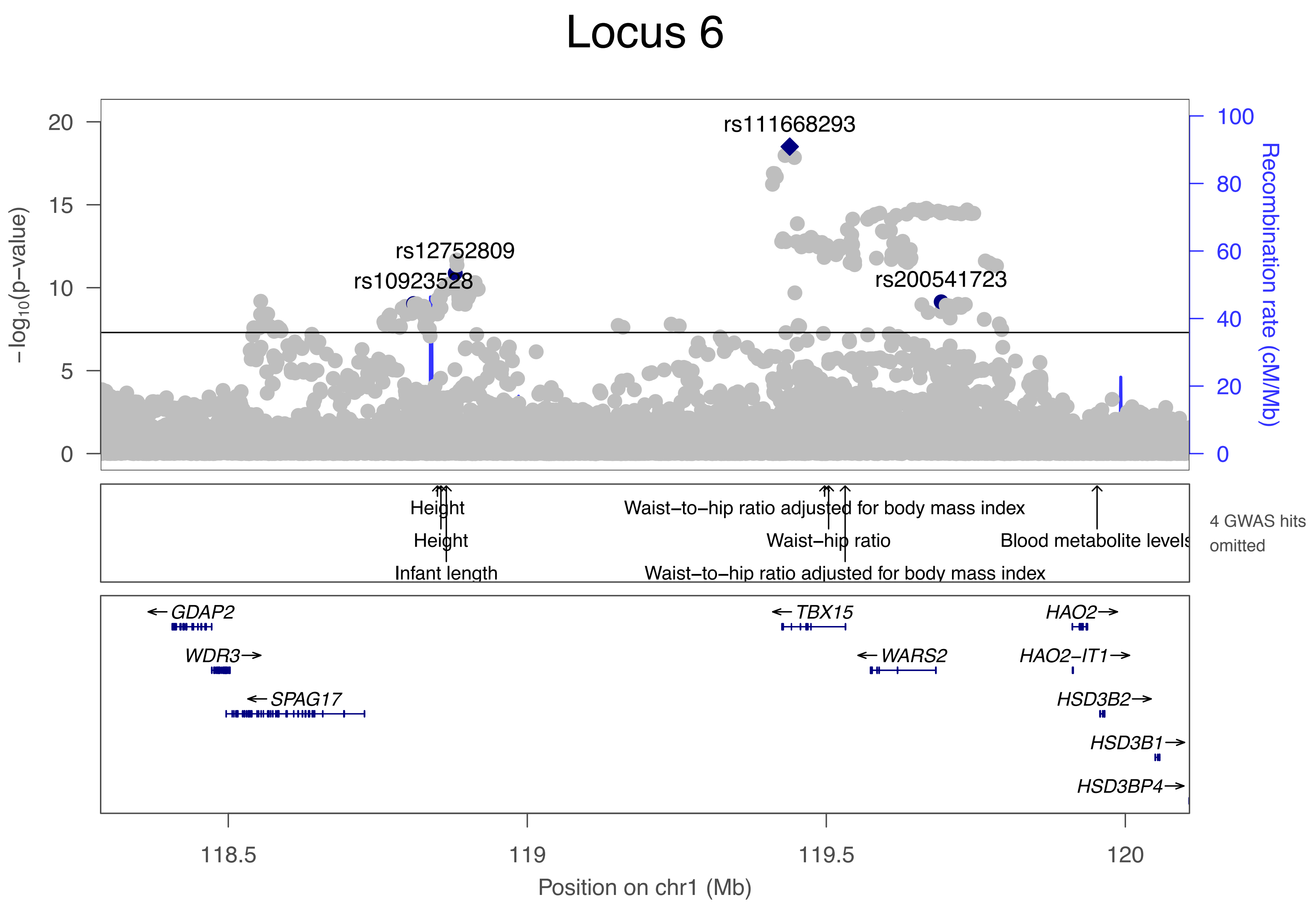
Locus 6
rs111668293
rs12752809
rs10923528
rs200541723
−log10(p−value)
Recombination rate (cM/Mb)
Height
Height
Infant length
Waist−to−hip ratio adjusted for body mass index
Waist−hip ratio
Waist−to−hip ratio adjusted for body mass index
Blood metabolite levels
4 GWAS hits omitted
GDAP2
WDR3
SPAG17
TBX15
WARS2
HAO2
HAO2−IT1
HSD3B2
HSD3B1
HSD3BP4
118.5
119
119.5
120
Position on chr1 (Mb)

Locus 7

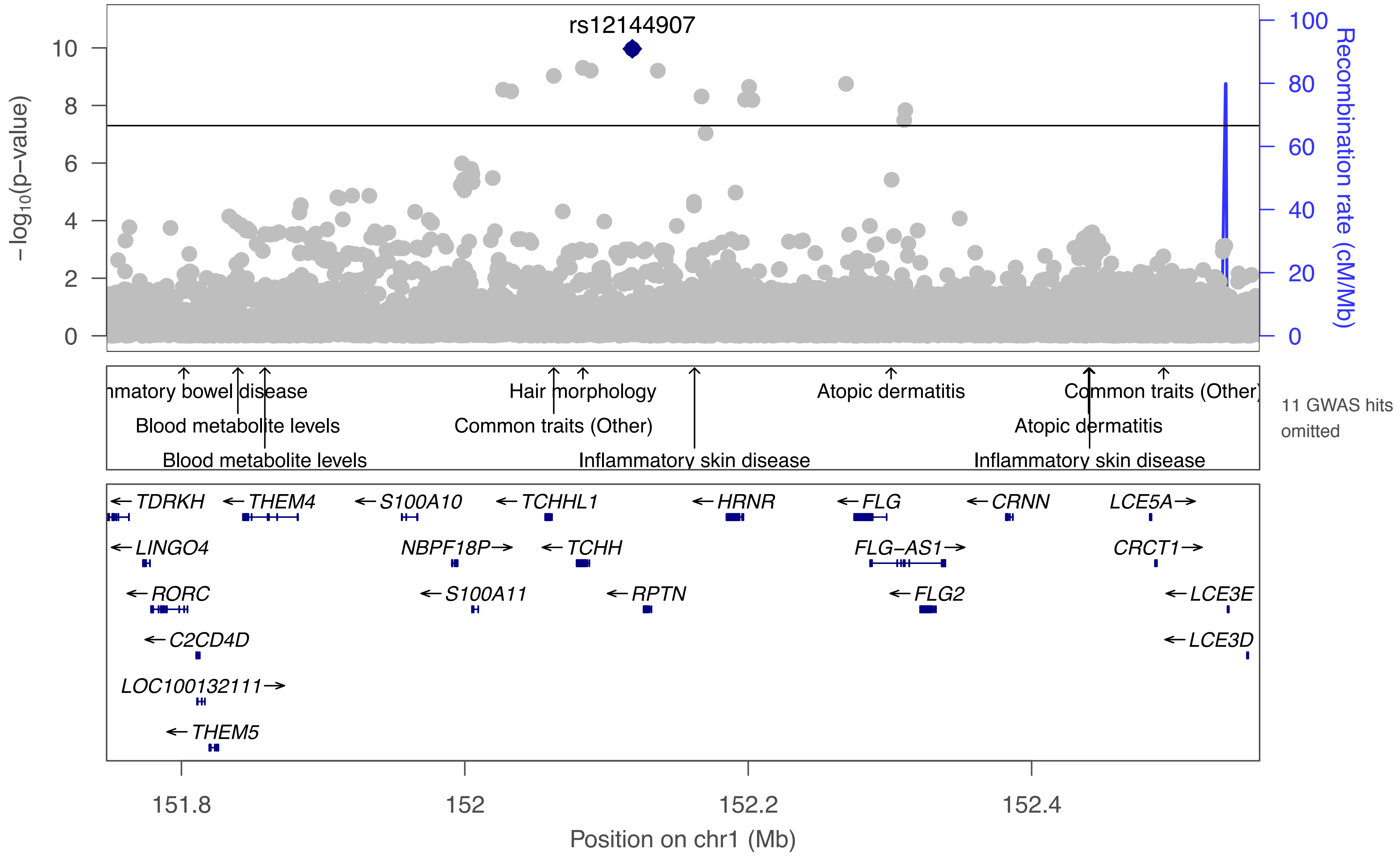
rs12144907
−log10(p−value)
Recombination rate (cM/Mb)
0
2
4
6
8
10
0
20
40
60
80
100
nmatory bowel disease
Blood metabolite levels
Blood metabolite levels
Hair morphology
Common traits (Other)
Inflammatory skin disease
Atopic dermatitis
Common traits (Other)
Atopic dermatitis
Inflammatory skin disease
11 GWAS hits omitted
TDRKH
LINGO4
RORC
C2CD4D
LOC100132111
THEM5
THEM4
S100A10
NBPF18P
S100A11
TCHHL1
TCHH
RPTN
HRNR
FLG
FLG−AS1
FLG2
CRNN
LCE5A
CRCT1
LCE3E
LCE3D
151.8
152
152.2
152.4
Position on chr1 (Mb)

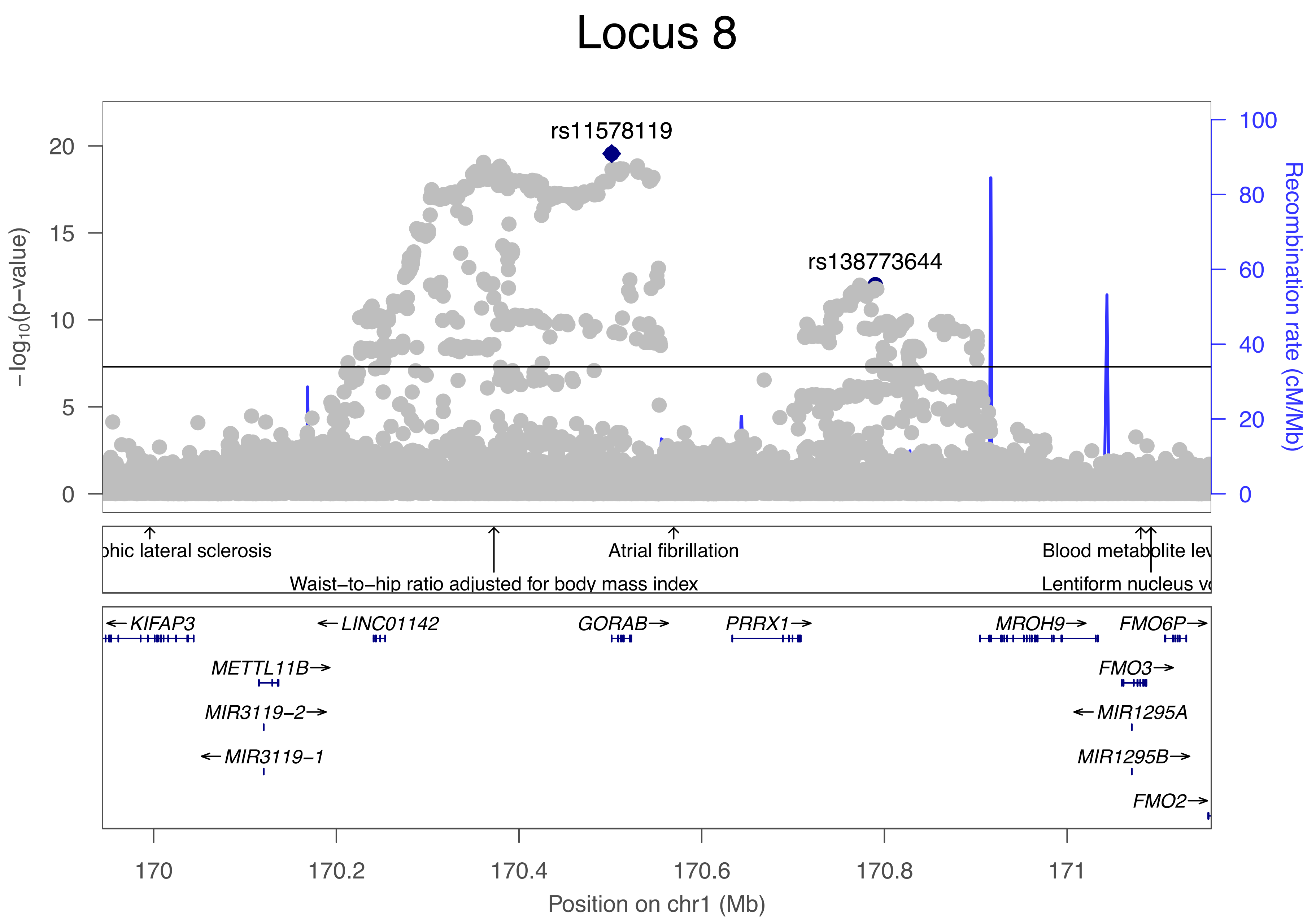
Locus 8
rs11578119
rs138773644
−log10(p−value)
Recombination rate (cM/Mb)
0
5
10
15
20
0
20
40
60
80
100
phic lateral sclerosis
Waist−to−hip ratio adjusted for body mass index
Atrial fibrillation
Blood metabolite lev
Lentiform nucleus v
KIFAP3
LINC01142
GORAB
PRRX1
MROH9
FMO6P
METTL11B
FMO3
MIR3119−2
MIR1295A
MIR3119−1
MIR1295B
FMO2
170
170.2
170.4
170.6
170.8
171
Position on chr1 (Mb)

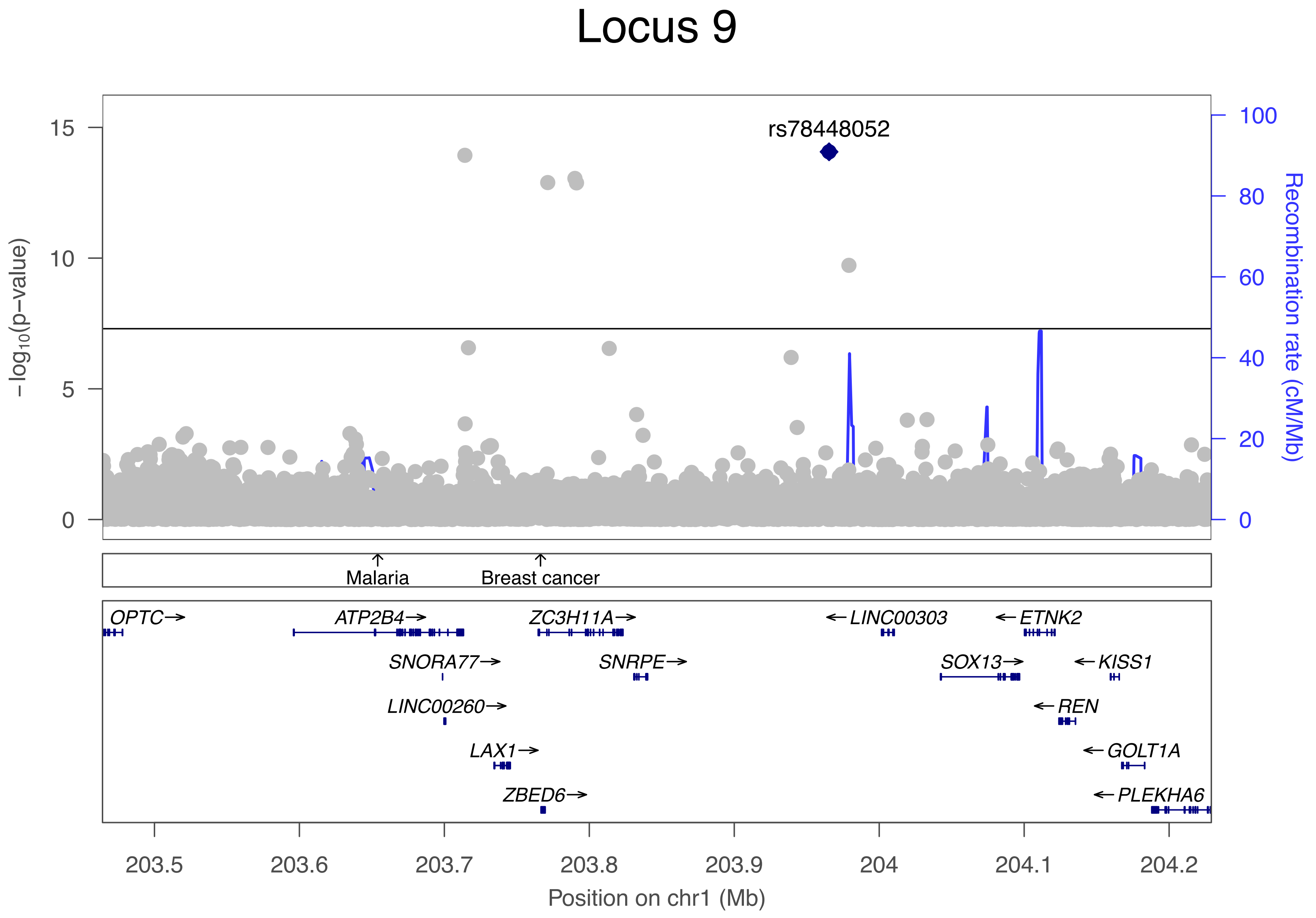
Locus 9
rs78448052
−log10(p−value)
Recombination rate (cM/Mb)
Malaria
Breast cancer
OPTC
ATP2B4
ZC3H11A
LINC00303
ETNK2
SNORA77
SNRPE
SOX13
KISS1
LINC00260
REN
LAX1
GOLT1A
ZBED6
PLEKHA6
203.5
203.6
203.7
203.8
203.9
204
204.1
204.2
Position on chr1 (Mb)

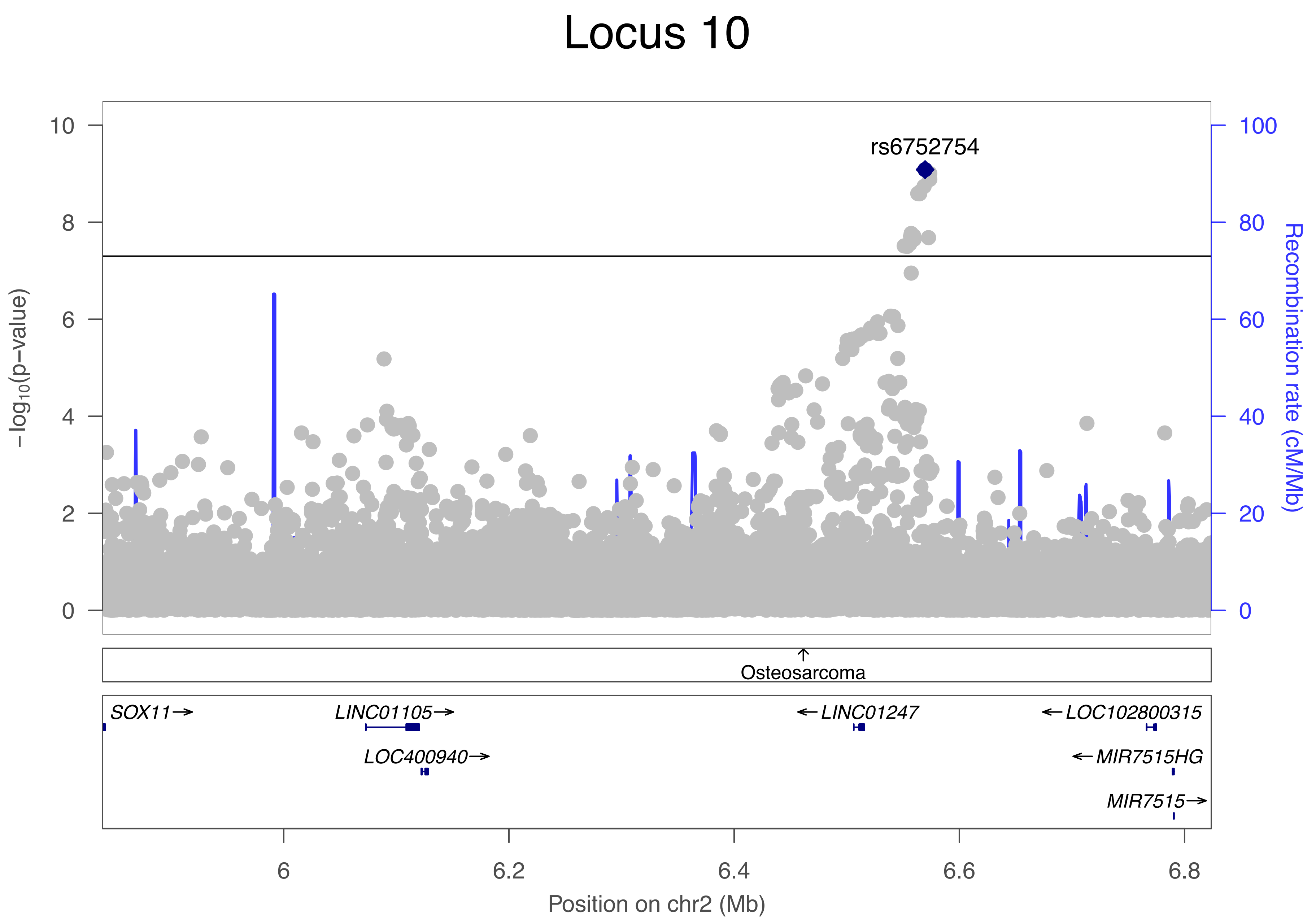
Locus 10
rs6752754
-log10(p-value)
Recombination rate (cM/Mb)
Osteosarcoma
SOX11
LINC01105
LOC400940
LINC01247
LOC102800315
MIR7515HG
MIR7515
Position on chr2 (Mb)

Locus 11

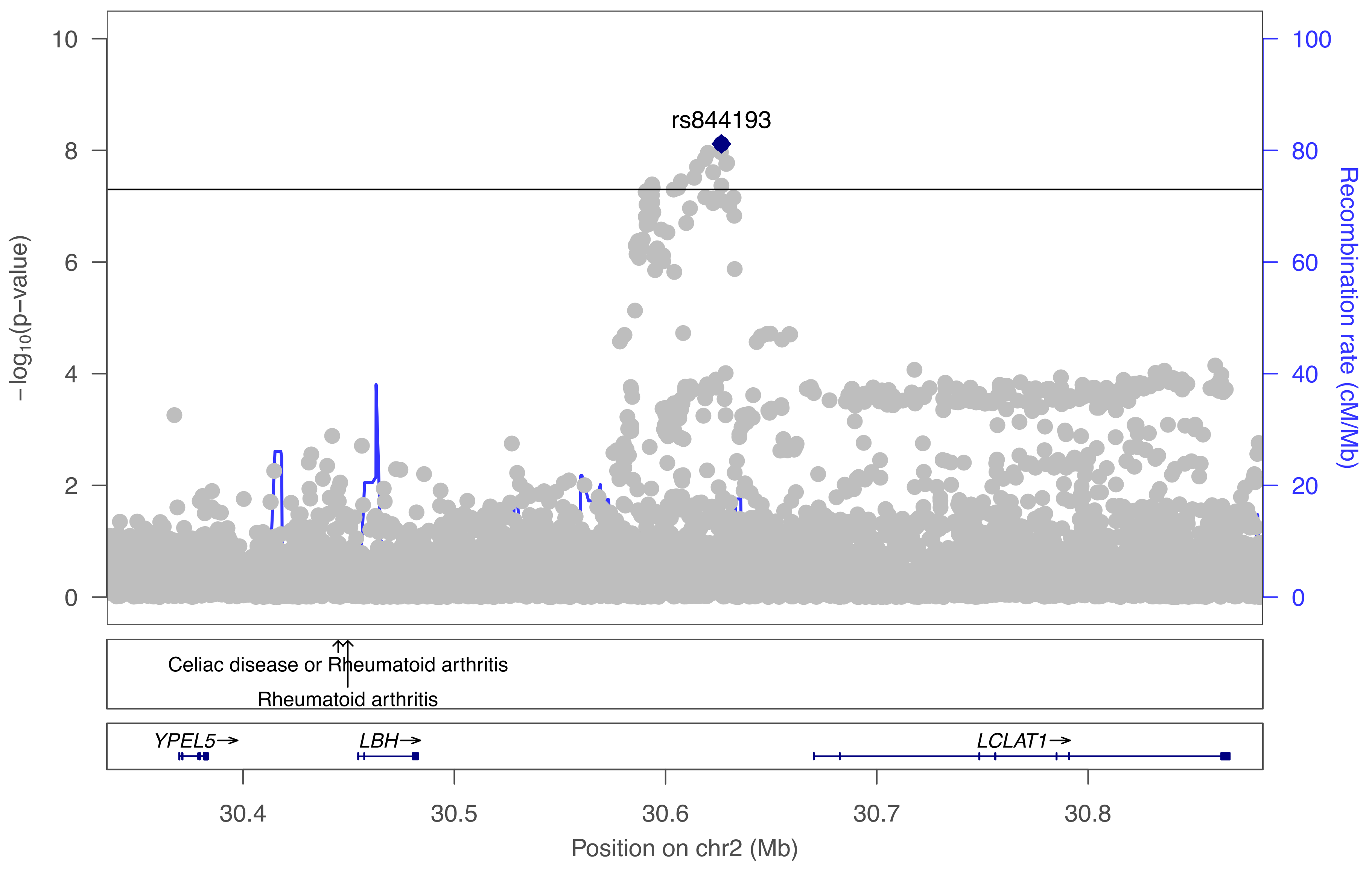
rs844193
−log10(p−value)
Recombination rate (cM/Mb)
0
2
4
6
8
10
0
20
40
60
80
100
Celiac disease or Rheumatoid arthritis
Rheumatoid arthritis
YPEL5→
LBH→
LCLAT1→
30.4
30.5
30.6
30.7
30.8
Position on chr2 (Mb)

Locus 12

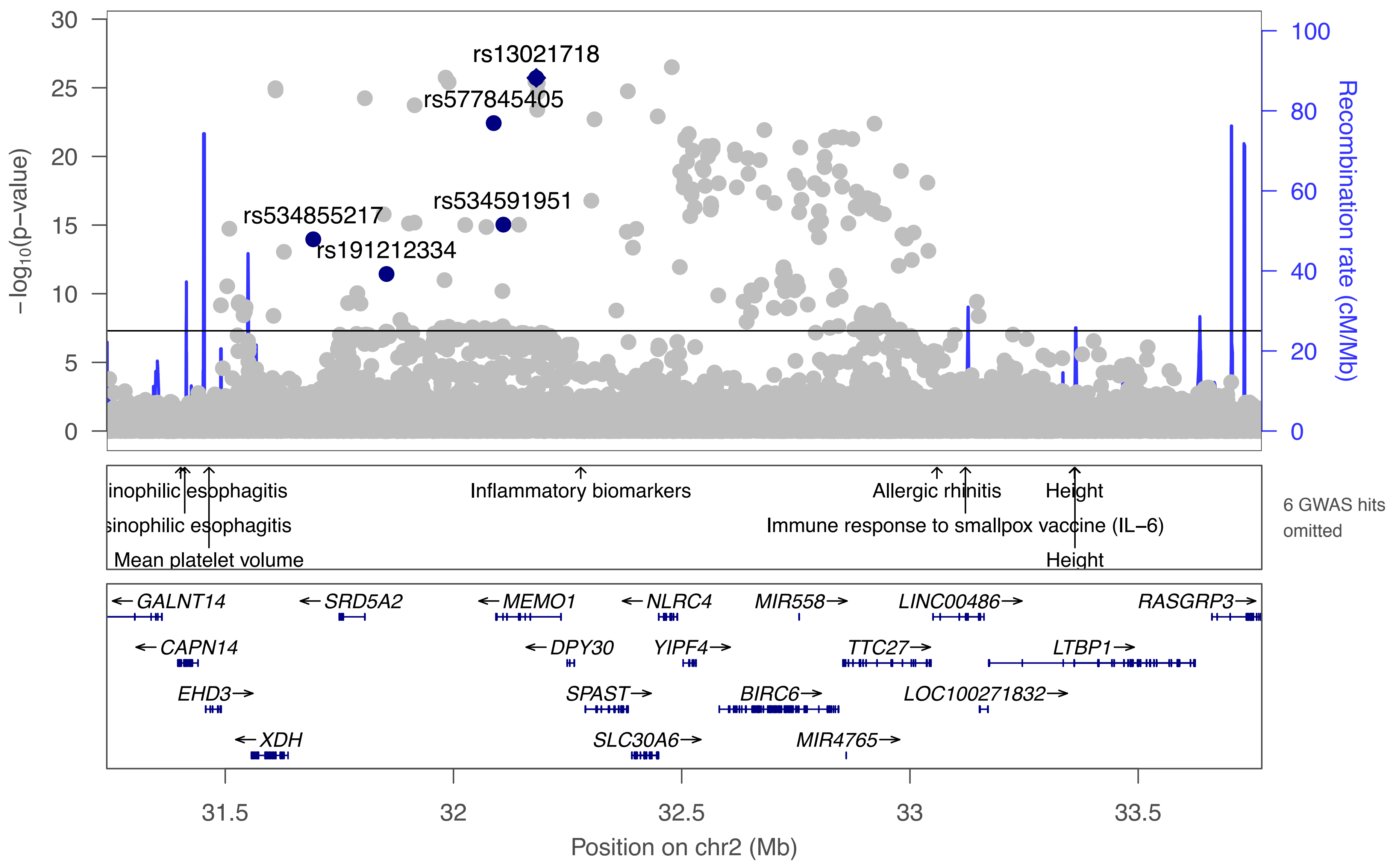

rs13021718
rs577845405
rs534591951
rs534855217
rs191212334
−log10(p−value)
Recombination rate (cM/Mb)
30
25
20
15
10
5
0
100
80
60
40
20
0
inophilic esophagitis
sinophilic esophagitis
Mean platelet volume
Inflammatory biomarkers
Allergic rhinitis
Height
Immune response to smallpox vaccine (IL−6)
Height
6 GWAS hits omitted
← GALNT14
← SRD5A2
← MEMO1
← NLRC4
MIR558 →
LINC00486 →
RASGRP3 →
← CAPN14
← DPY30
YIPF4 →
TTC27 →
LTBP1 →
EHD3 →
SPAST →
BIRC6 →
LOC100271832 →
← XDH
SLC30A6 →
MIR4765 →
31.5
32
32.5
33
33.5
Position on chr2 (Mb)

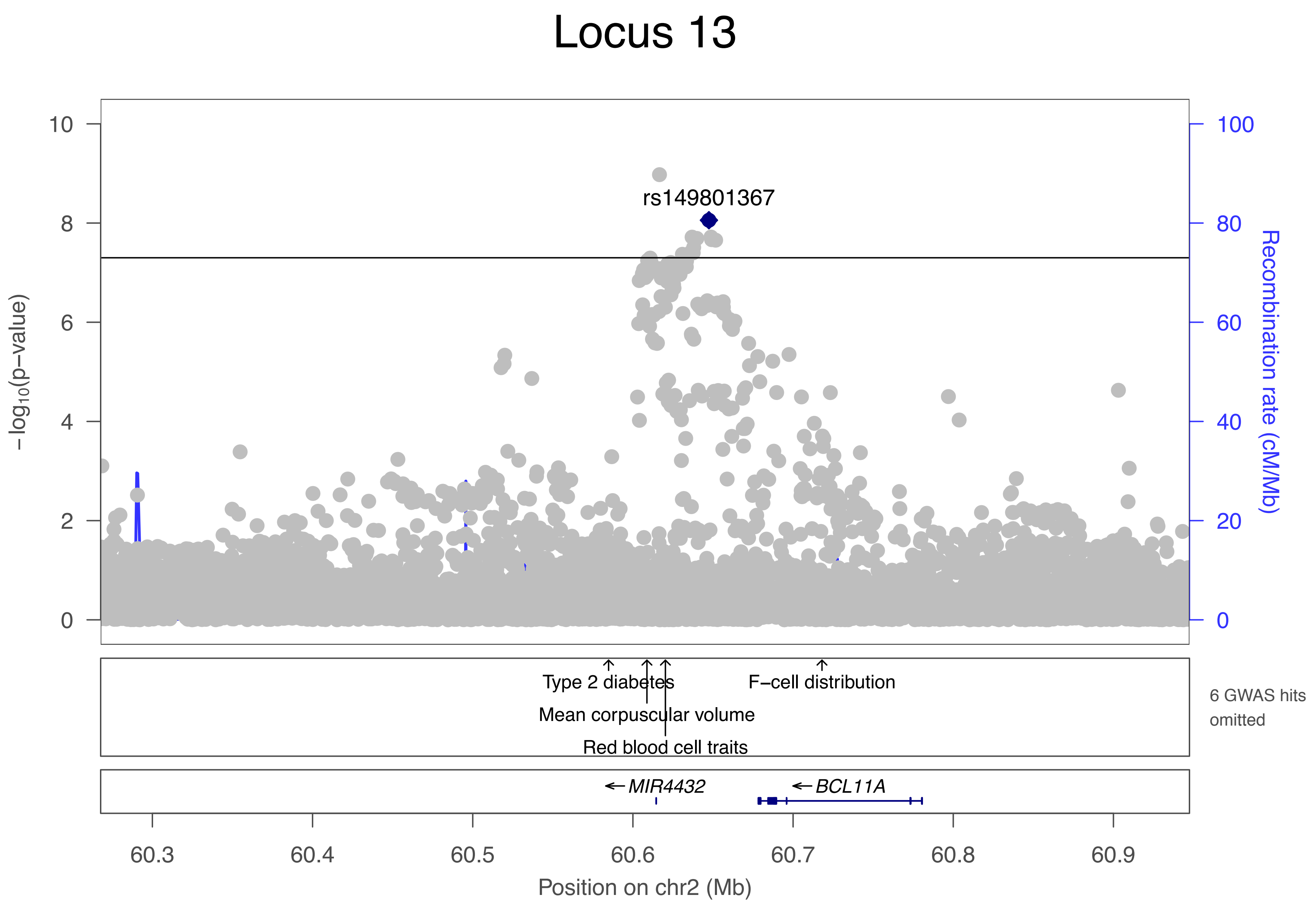

Locus 13
rs149801367
−log10(p−value)
Recombination rate (cM/Mb)
0
2
4
6
8
10
0
20
40
60
80
100
Type 2 diabetes
Mean corpuscular volume
Red blood cell traits
F−cell distribution
6 GWAS hits omitted
MIR4432
BCL11A
60.3
60.4
60.5
60.6
60.7
60.8
60.9
Position on chr2 (Mb)

Locus 14

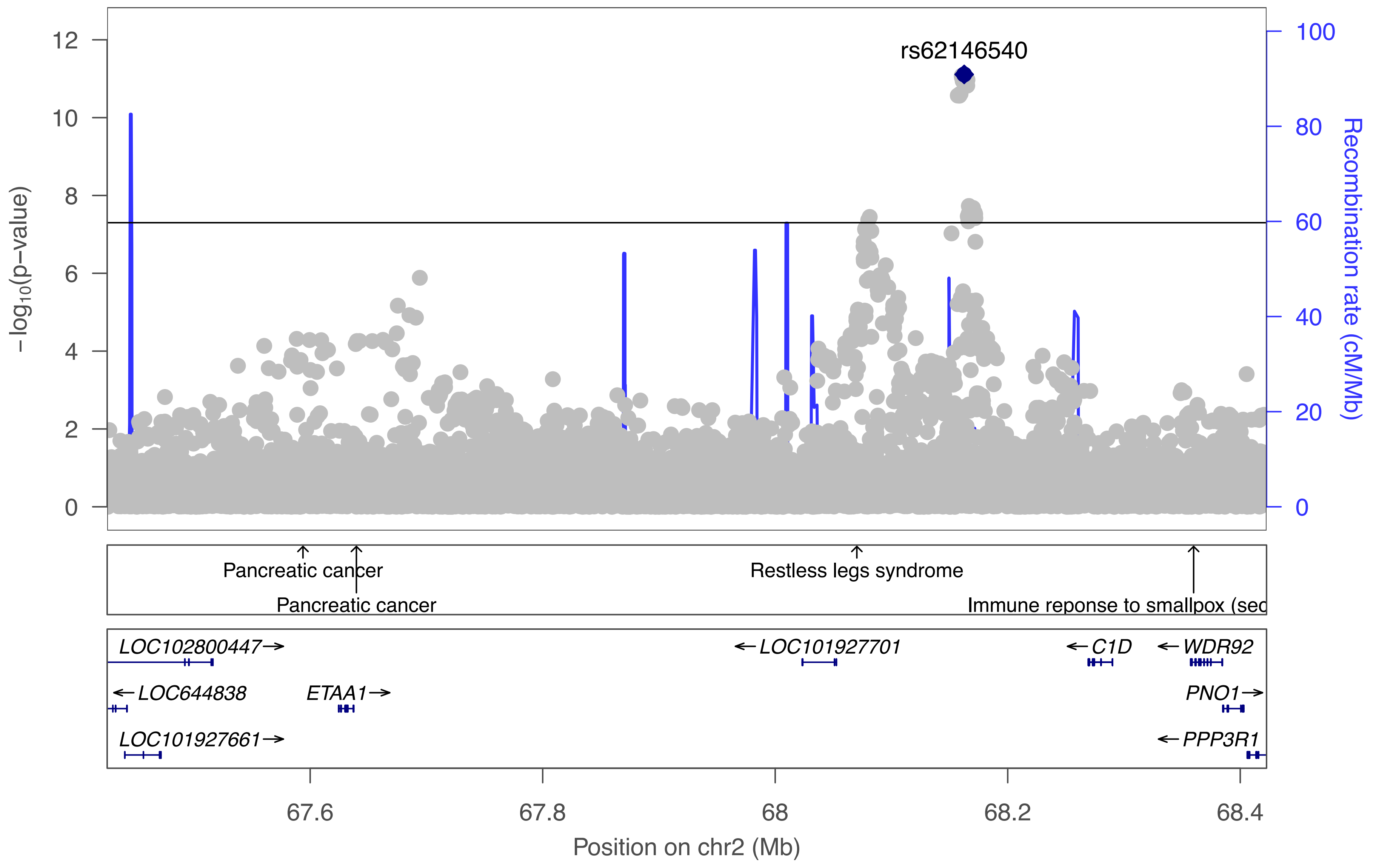

rs62146540
−log10(p−value)
Recombination rate (cM/Mb)
12
10
8
6
4
2
0
100
80
60
40
20
0
Pancreatic cancer
Pancreatic cancer
Restless legs syndrome
Immune reponse to smallpox (sec
LOC102800447
LOC644838
LOC101927661
ETAA1
LOC101927701
C1D
WDR92
PNO1
PPP3R1
67.6
67.8
68
68.2
68.4
Position on chr2 (Mb)

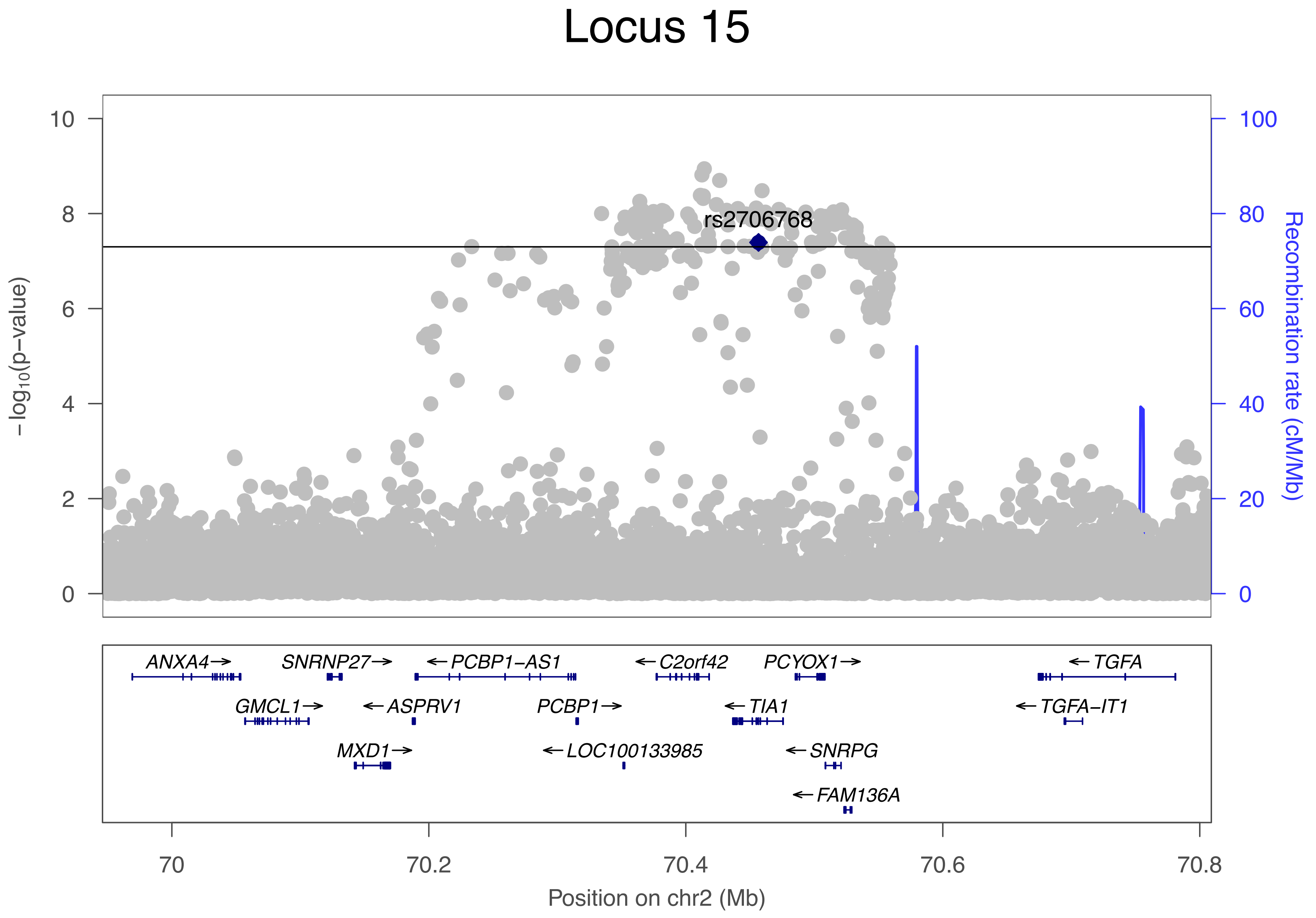

Locus 15
rs2706768
−log10(p−value)
Recombination rate (cM/Mb)
0
2
4
6
8
10
0
20
40
60
80
100
ANXA4→
SNRNP27→
←PCBP1−AS1
←C2orf42
PCYOX1→
←TGFA
GMCL1→
←ASPRV1
PCBP1→
←TIA1
←TGFA−IT1
MXD1→
←LOC100133985
←SNRPG
←FAM136A
70
70.2
70.4
70.6
70.8
Position on chr2 (Mb)

Locus 16

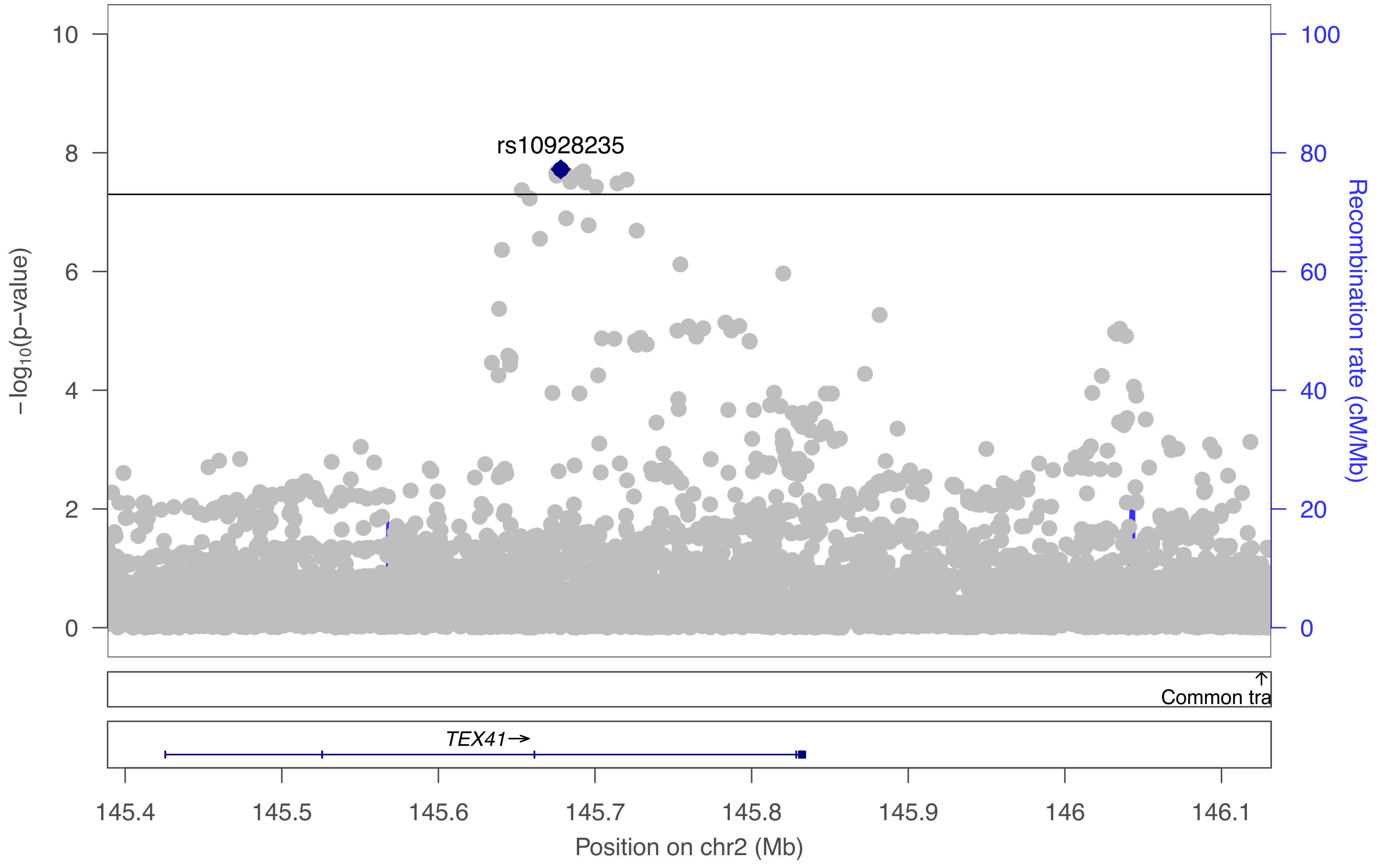

rs10928235
−log10(p−value)
Recombination rate (cM/Mb)
10
8
6
4
2
0
100
80
60
40
20
0
Common tra
TEX41→
145.4
145.5
145.6
145.7
145.8
145.9
146
146.1
Position on chr2 (Mb)

Locus 17

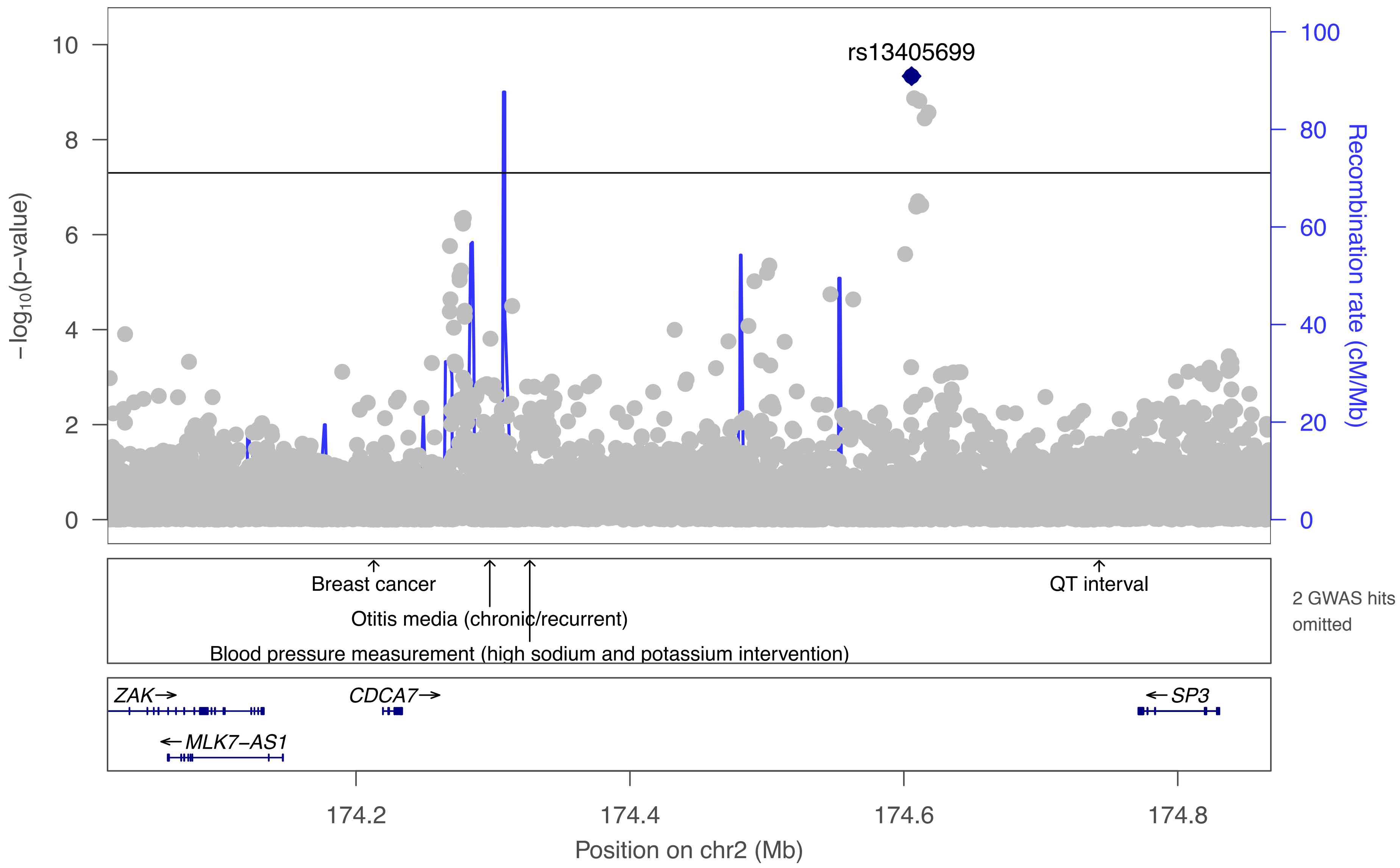

rs13405699
10
8
6
4
2
0
−log10(p−value)
100
80
60
40
20
0
Recombination rate (cM/Mb)
Breast cancer
Otitis media (chronic/recurrent)
Blood pressure measurement (high sodium and potassium intervention)
QT interval
2 GWAS hits omitted
ZAK→
CDCA7→
←SP3
←MLK7−AS1
174.2
174.4
174.6
174.8
Position on chr2 (Mb)

Locus 18

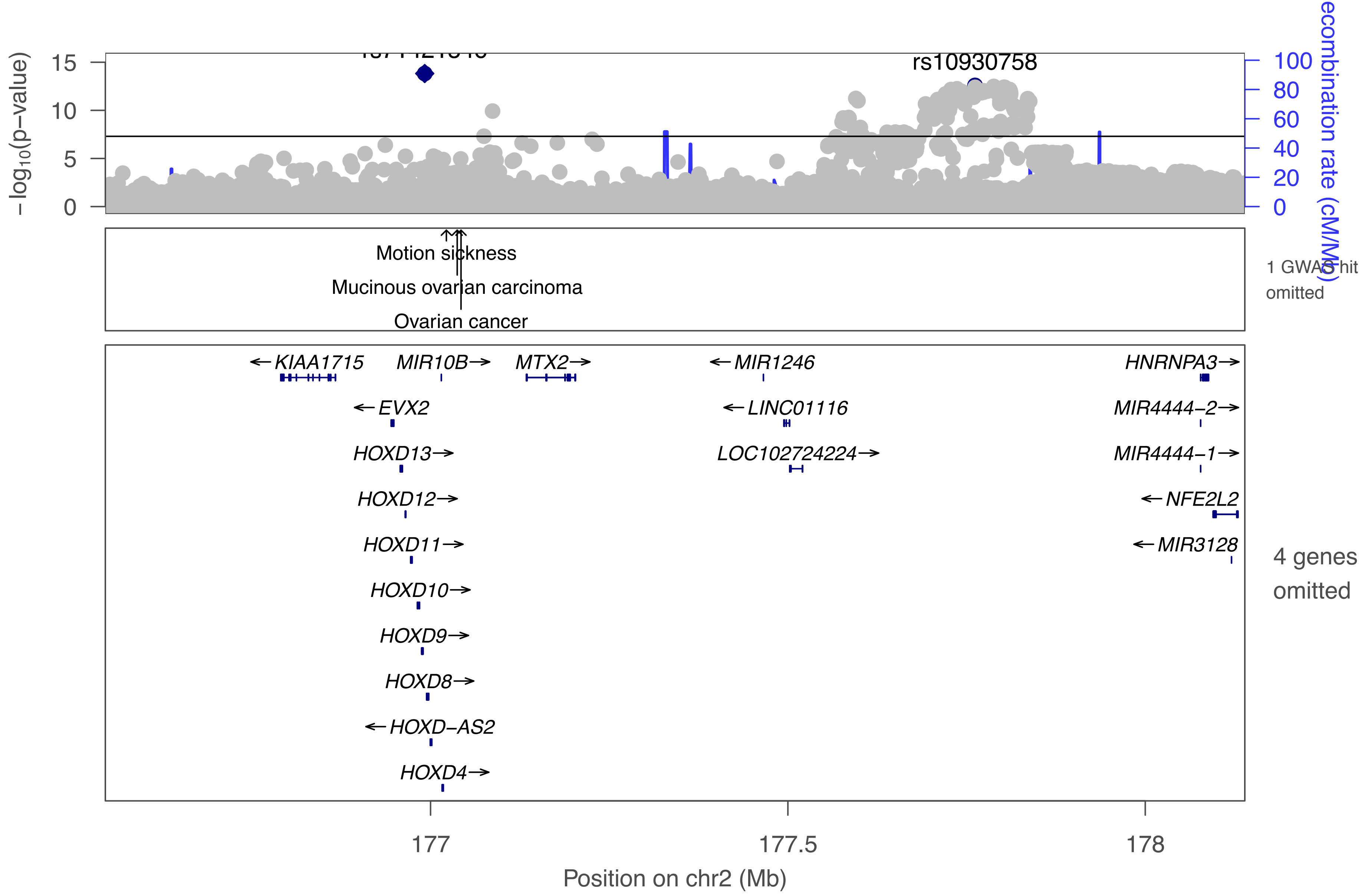

rs10930758
−log10(p−value)
15
10
5
0
Recombination rate (cM/Mb)
100
80
60
40
20
0
Motion sickness
Mucinous ovarian carcinoma
Ovarian cancer
1 GWAS hit omitted
←KIAA1715
MIR10B→
MTX2→
←MIR1246
HNRNPA3→
←EVX2
←LINC01116
MIR4444−2→
HOXD13→
LOC102724224→
MIR4444−1→
HOXD12→
←NFE2L2
HOXD11→
←MIR3128
HOXD10→
HOXD9→
HOXD8→
←HOXD−AS2
HOXD4→
4 genes omitted
177
177.5
178
Position on chr2 (Mb)

Locus 19

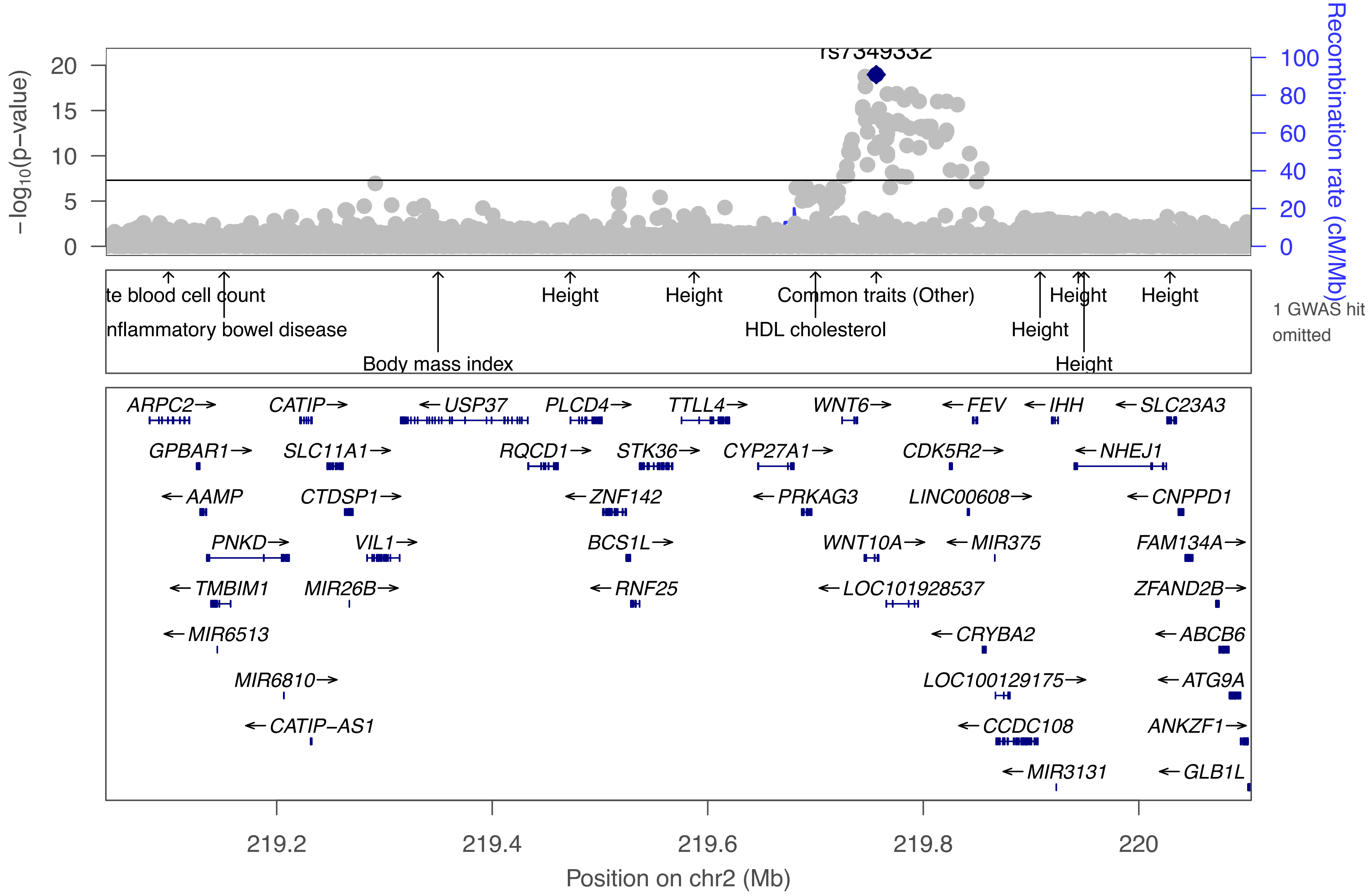
rs7349332
−log10(p−value)
20
15
10
5
0
Recombination rate (cM/Mb)
100
80
60
40
20
0
te blood cell count
nflammatory bowel disease
Body mass index
Height
Height
Common traits (Other)
HDL cholesterol
Height
Height
Height
Height
1 GWAS hit omitted
ARPC2→
CATIP→
←USP37
PLCD4→
TTLL4→
WNT6→
←FEV
←IHH
←SLC23A3
GPBAR1→
SLC11A1→
RQCD1→
STK36→
CYP27A1→
CDK5R2→
←NHEJ1
←AAMP
CTDSP1→
←ZNF142
←PRKAG3
LINC00608→
←CNPPD1
PNKD→
VIL1→
BCS1L→
WNT10A→
←MIR375
FAM134A→
←TMBIM1
MIR26B→
←RNF25
←LOC101928537
ZFAND2B→
←MIR6513
←CRYBA2
←ABCB6
MIR6810→
LOC100129175→
←ATG9A
←CATIP−AS1
←CCDC108
ANKZF1→
←MIR3131
←GLB1L
219.2
219.4
219.6
219.8
220
Position on chr2 (Mb)

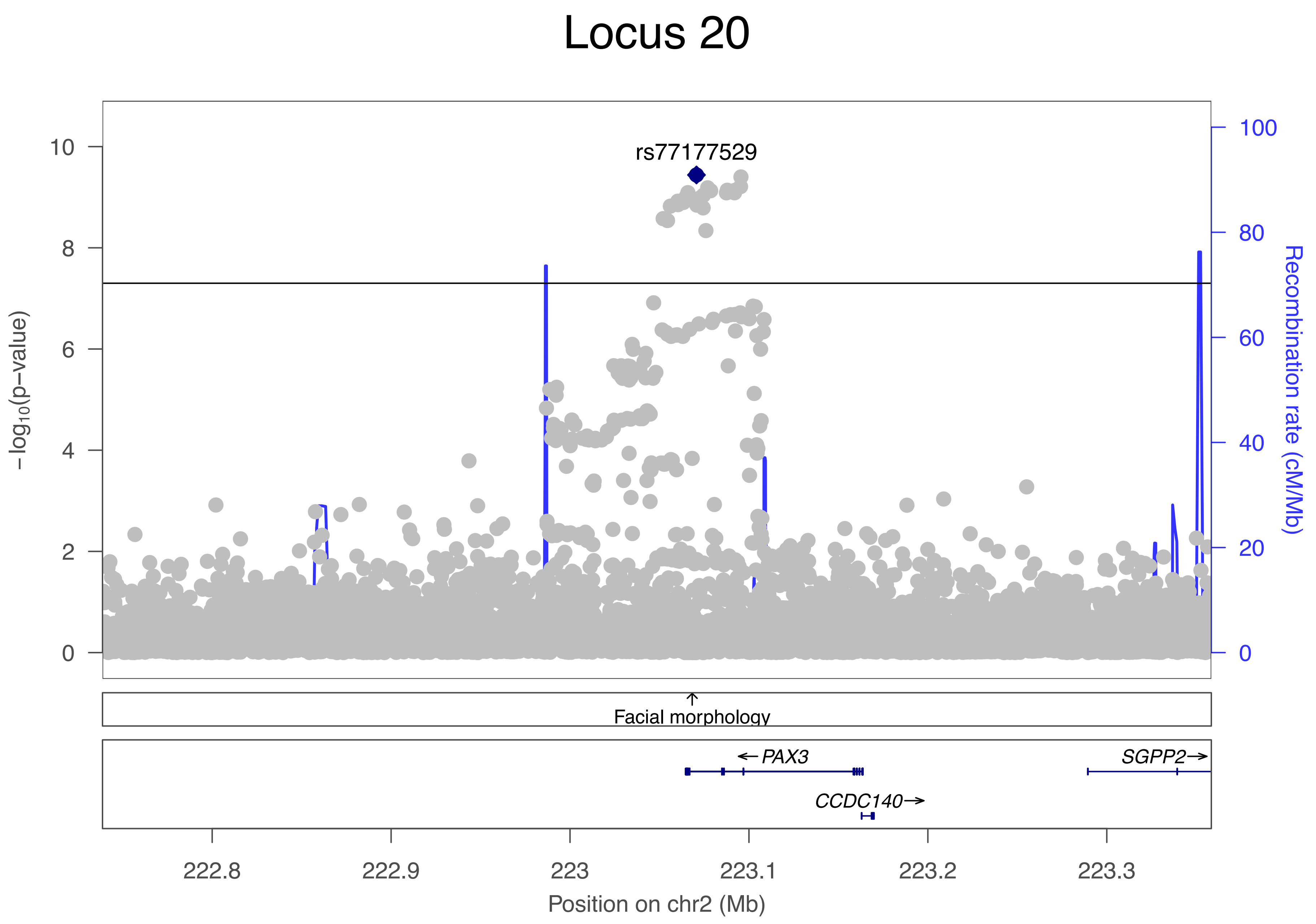
Locus 20
rs77177529
10
8
6
4
2
0
−log10(p−value)
100
80
60
40
20
0
Recombination rate (cM/Mb)
Facial morphology
← PAX3
SGPP2→
CCDC140→
222.8
222.9
223
223.1
223.2
223.3
Position on chr2 (Mb)

Locus 21

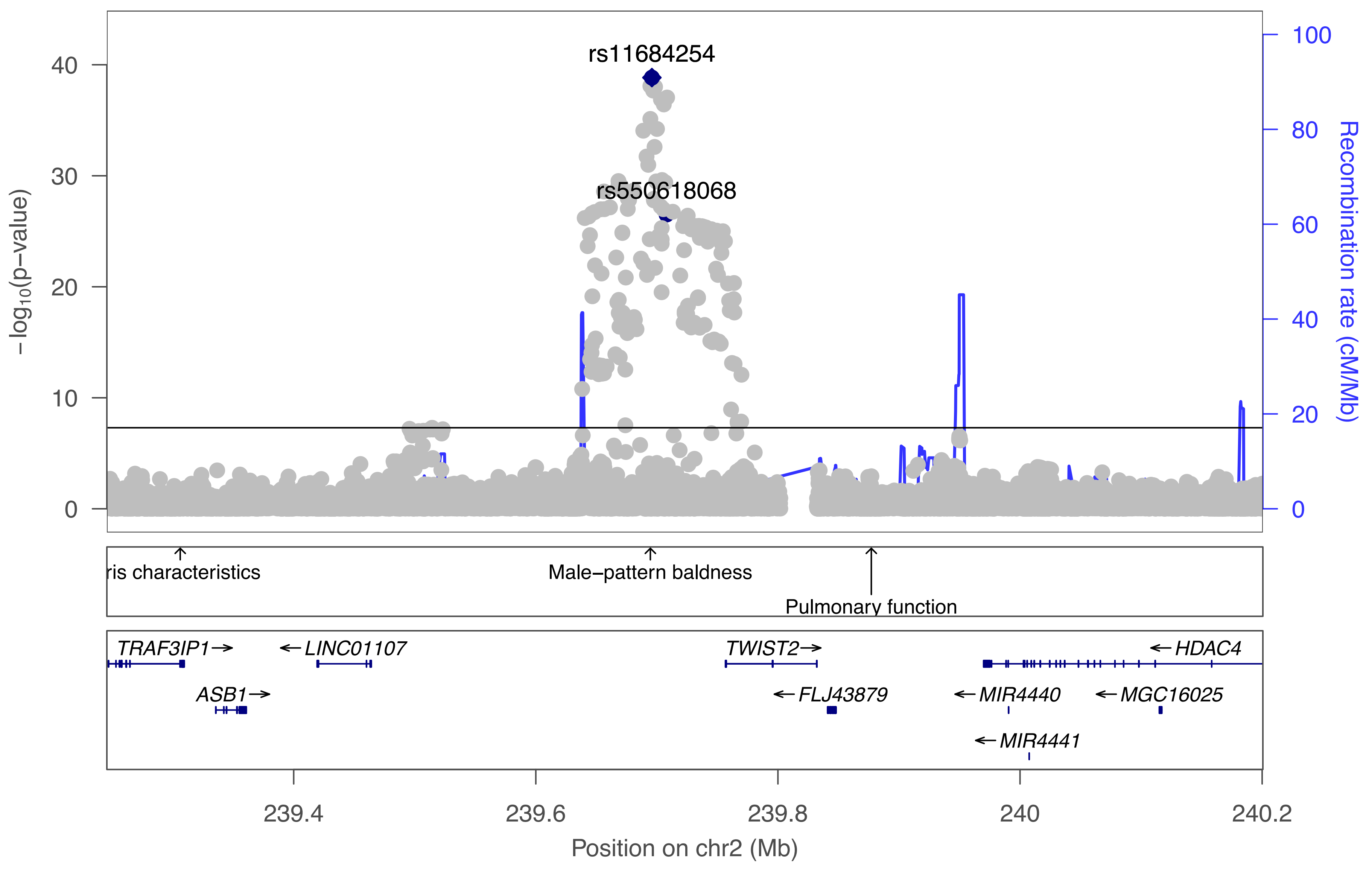
rs11684254
rs550618068
−log10(p−value)
Recombination rate (cM/Mb)
0
10
20
30
40
0
20
40
60
80
100
ris characteristics
Male−pattern baldness
Pulmonary function
TRAF3IP1→
←LINC01107
TWIST2→
←HDAC4
ASB1→
←FLJ43879
←MIR4440
←MGC16025
←MIR4441
239.4
239.6
239.8
240
240.2
Position on chr2 (Mb)

Locus 22

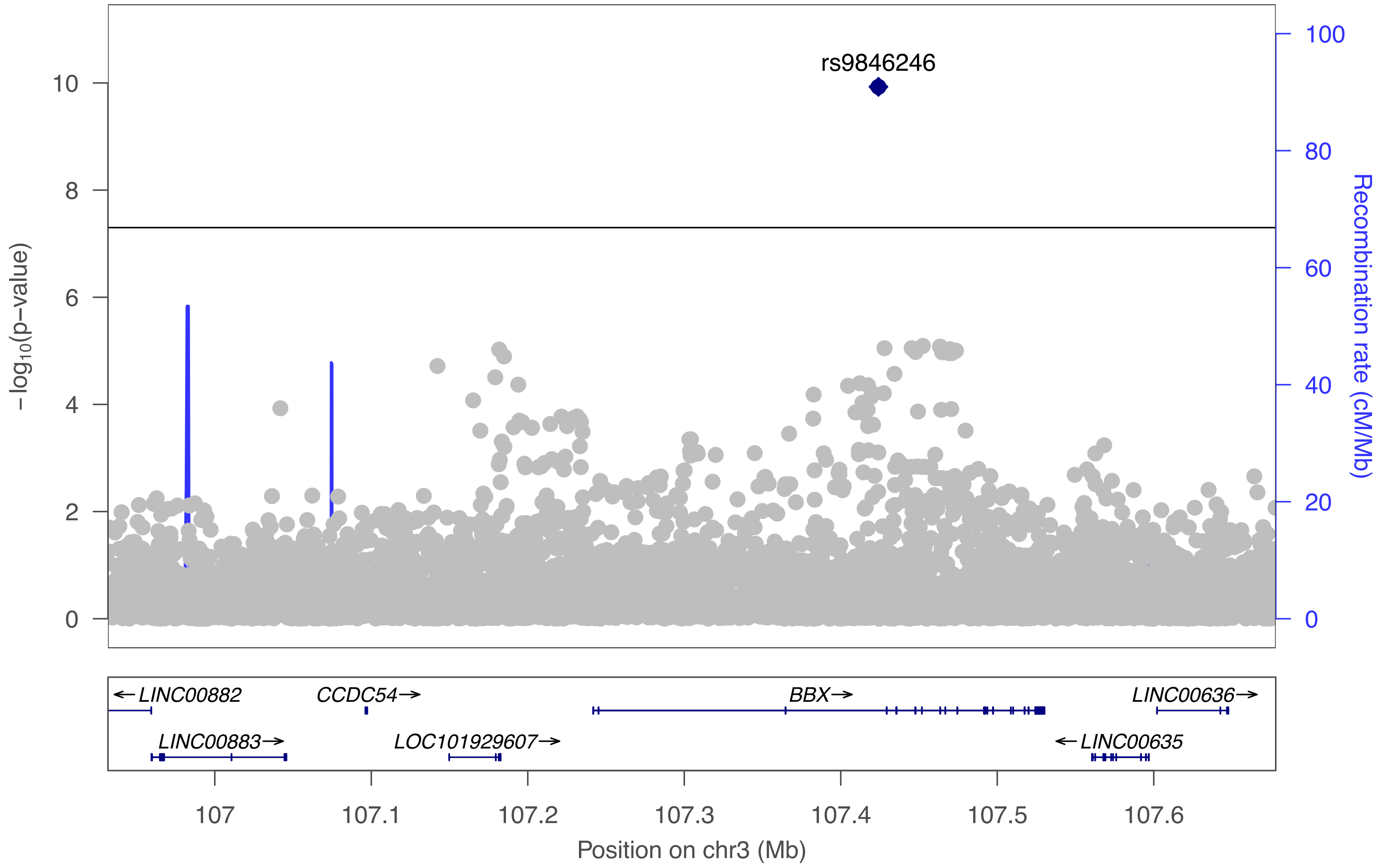

rs9846246
−log10(p−value)
Recombination rate (cM/Mb)
0
2
4
6
8
10
0
20
40
60
80
100
←LINC00882
CCDC54→
BBX→
LINC00636→
LINC00883→
LOC101929607→
←LINC00635
107
107.1
107.2
107.3
107.4
107.5
107.6
Position on chr3 (Mb)

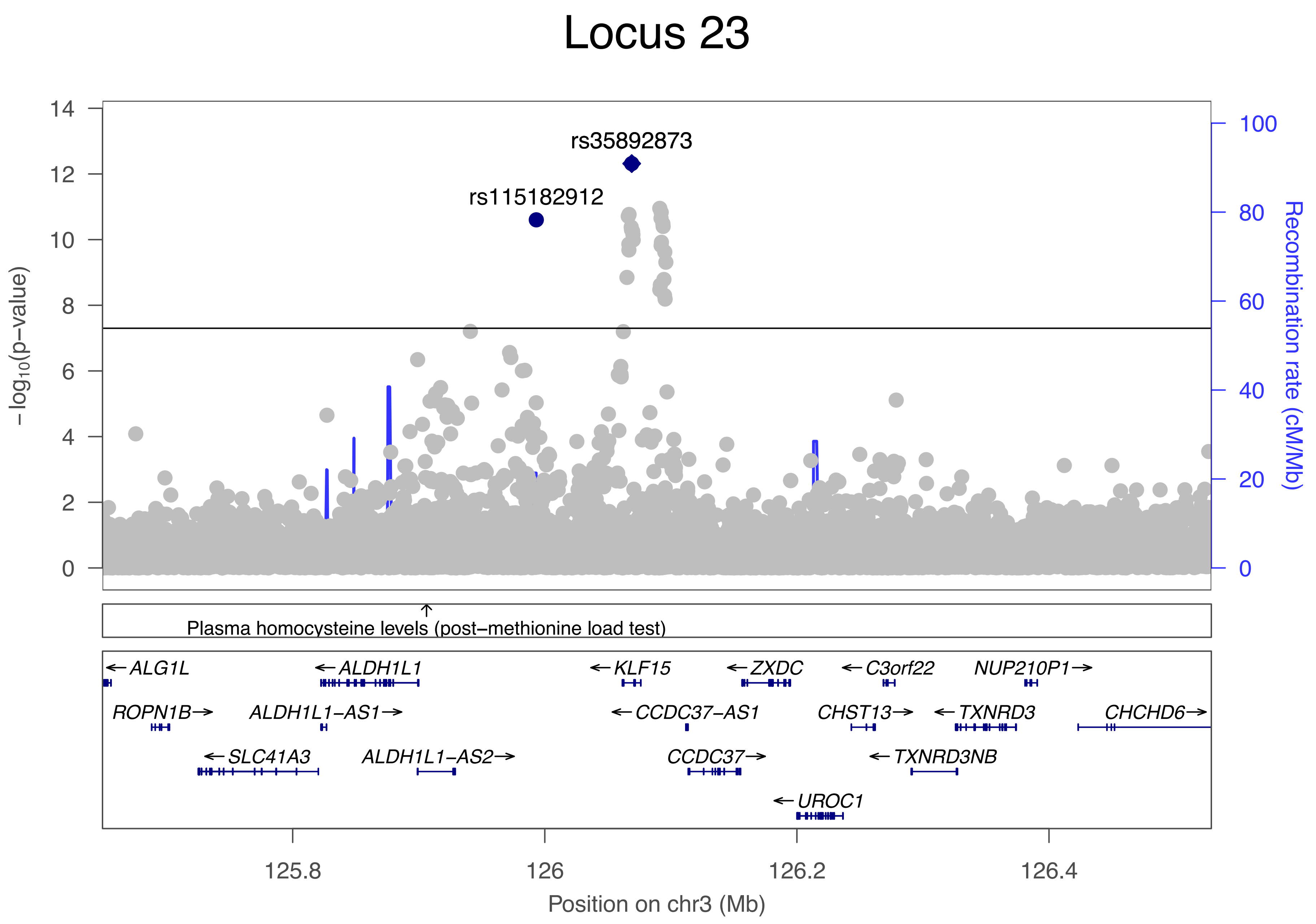

Locus 23
rs35892873
rs115182912
14
12
10
8
6
4
2
0
−log10(p−value)
100
80
60
40
20
0
Recombination rate (cM/Mb)
Plasma homocysteine levels (post−methionine load test)
←ALG1L
←ALDH1L1
←KLF15
←ZXDC
←C3orf22
NUP210P1→
ROPN1B→
ALDH1L1−AS1→
←CCDC37−AS1
CHST13→
←TXNRD3
CHCHD6→
←SLC41A3
ALDH1L1−AS2→
CCDC37→
←TXNRD3NB
←UROC1
125.8
126
126.2
126.4
Position on chr3 (Mb)

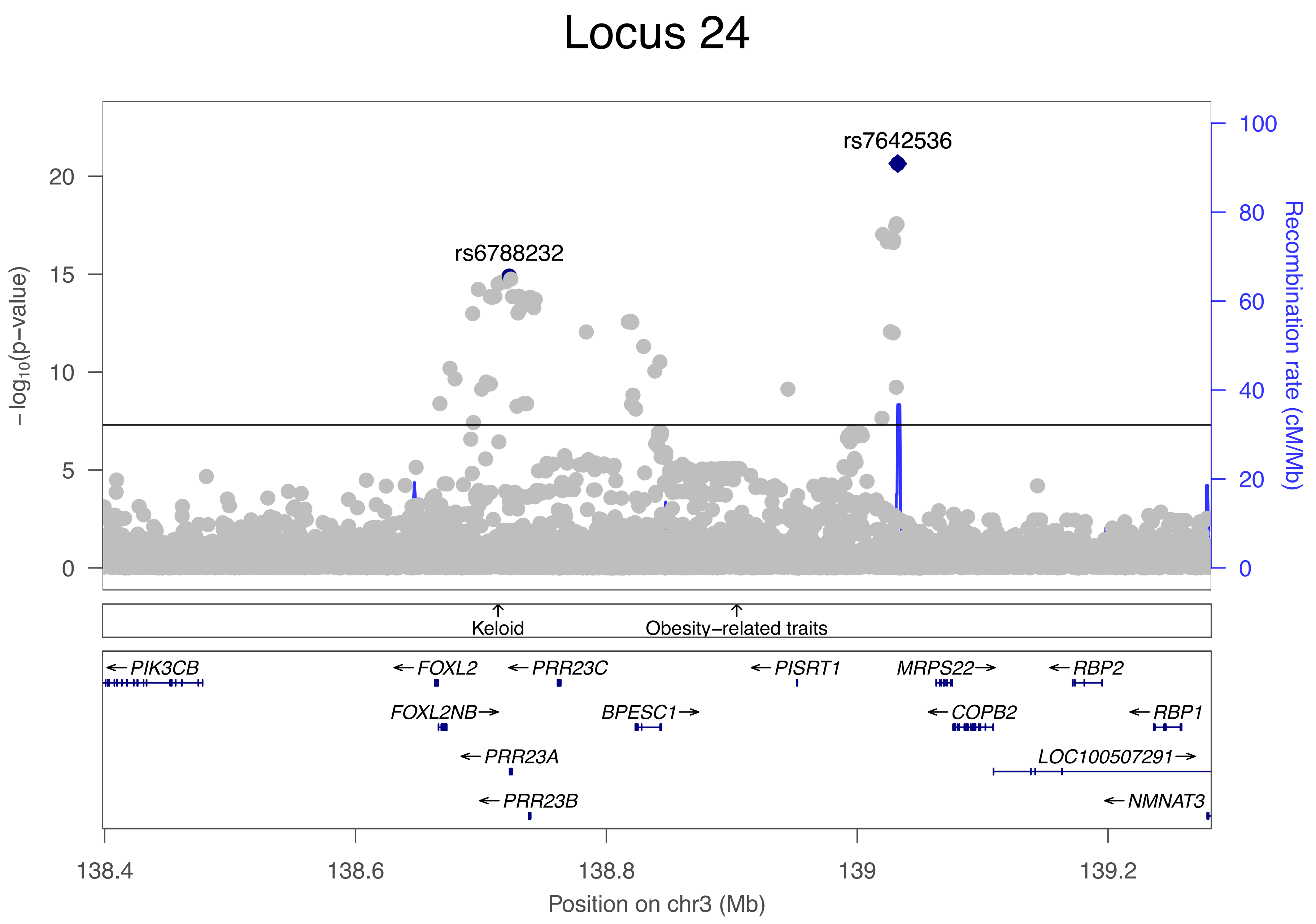
Locus 24
rs7642536
rs6788232
−log10(p−value)
Recombination rate (cM/Mb)
0
5
10
15
20
0
20
40
60
80
100
Keloid
Obesity−related traits
PIK3CB
FOXL2
PRR23C
PISRT1
MRPS22
RBP2
FOXL2NB
BPESC1
COPB2
RBP1
PRR23A
LOC100507291
PRR23B
NMNAT3
138.4
138.6
138.8
139
139.2
Position on chr3 (Mb)

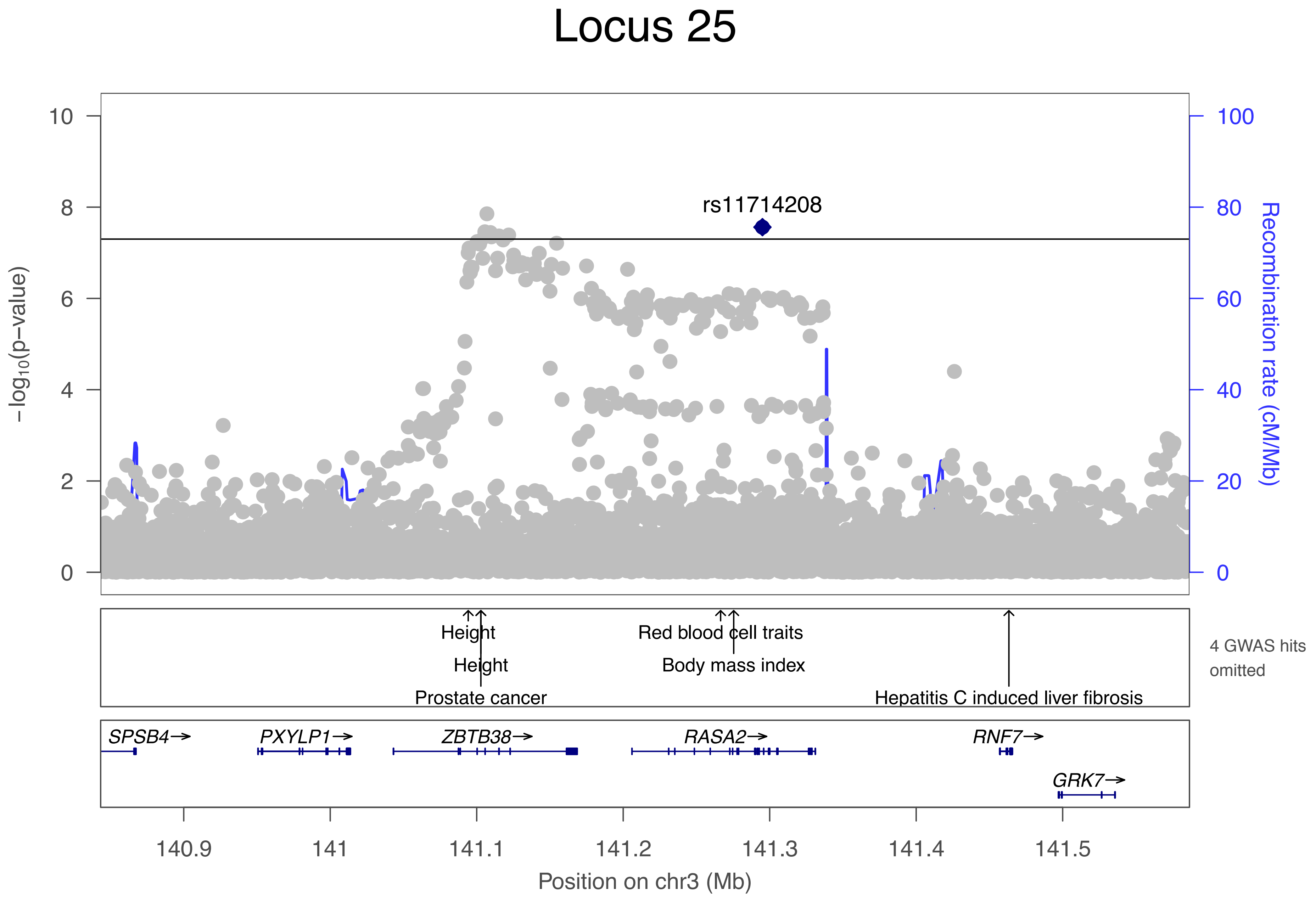
Locus 25
rs11714208
−log10(p−value)
Recombination rate (cM/Mb)
10
8
6
4
2
0
100
80
60
40
20
0
Height
Height
Prostate cancer
Red blood cell traits
Body mass index
Hepatitis C induced liver fibrosis
4 GWAS hits omitted
SPSB4→
PXYLP1→
ZBTB38→
RASA2→
RNF7→
GRK7→
140.9
141
141.1
141.2
141.3
141.4
141.5
Position on chr3 (Mb)

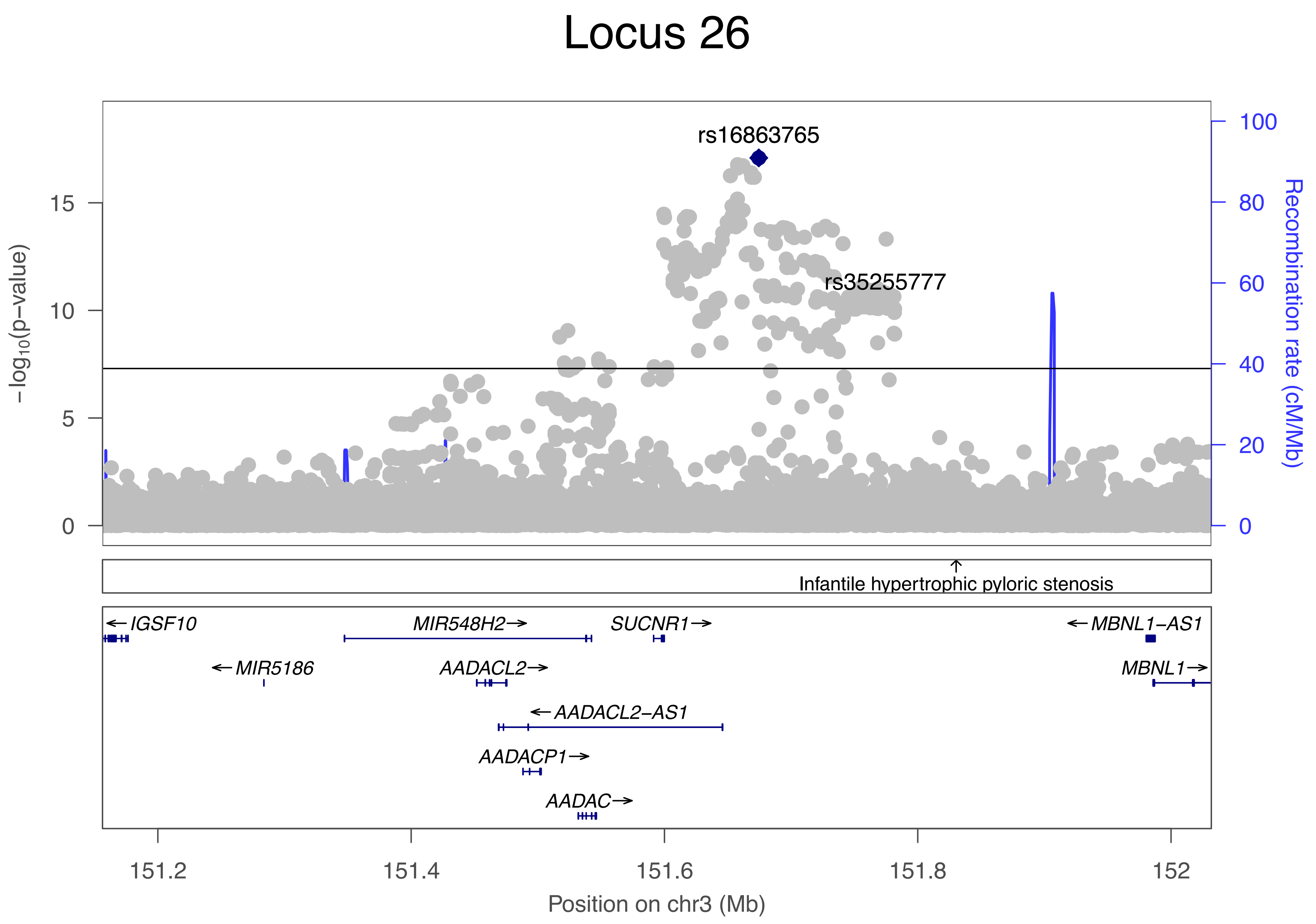

Locus 26
rs16863765
rs35255777
−log10(p−value)
Recombination rate (cM/Mb)
0
5
10
15
0
20
40
60
80
100
Infantile hypertrophic pyloric stenosis
←IGSF10
MIR548H2→
SUCNR1→
←MBNL1−AS1
←MIR5186
AADACL2→
MBNL1→
←AADACL2−AS1
AADACP1→
AADAC→
151.2
151.4
151.6
151.8
152
Position on chr3 (Mb)

Locus 27

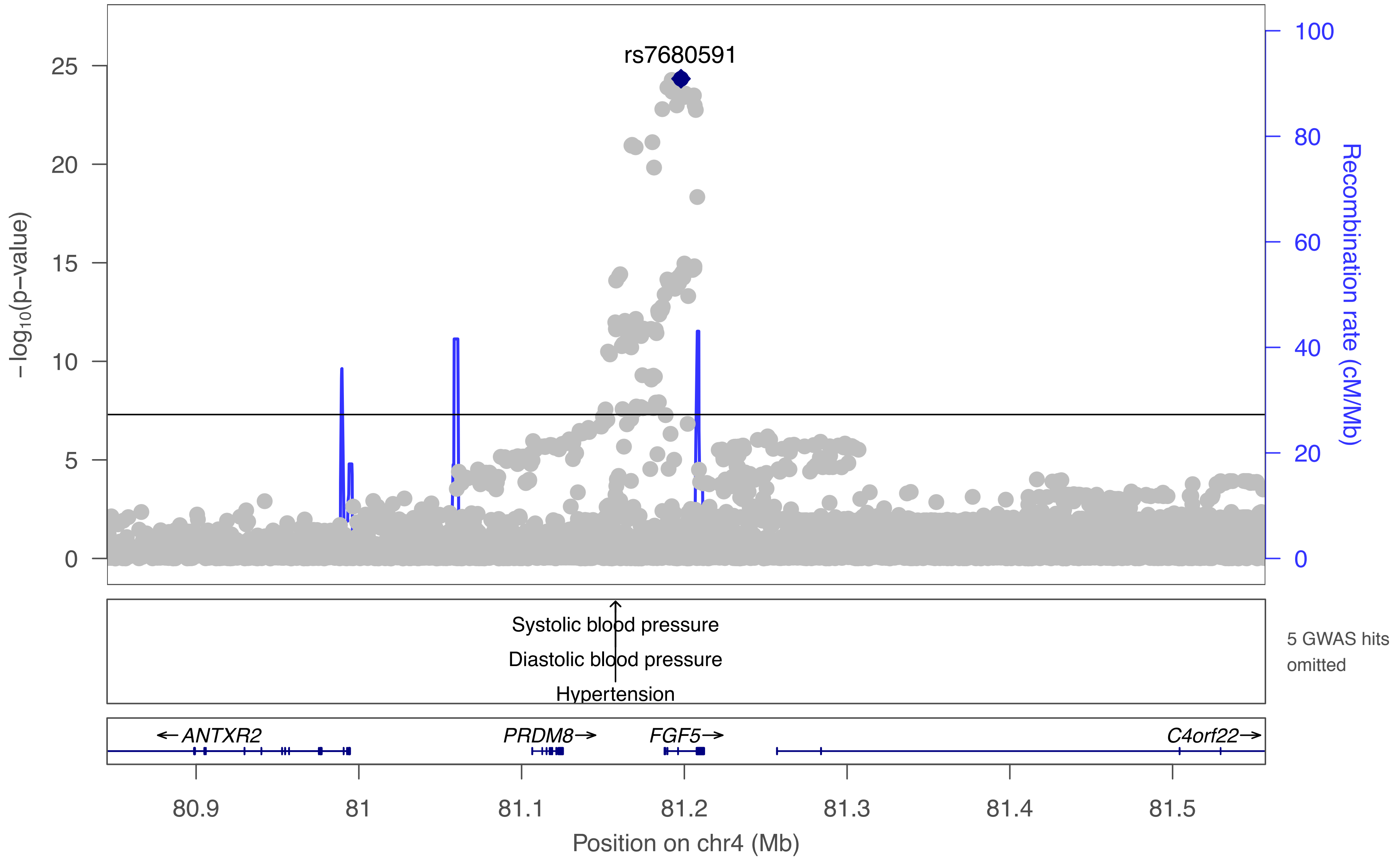

rs7680591
25
20
15
10
5
0
−log10(p−value)
100
80
60
40
20
0
Recombination rate (cM/Mb)
Systolic blood pressure
Diastolic blood pressure
Hypertension
5 GWAS hits omitted
← ANTXR2
PRDM8→
FGF5→
C4orf22→
80.9
81
81.1
81.2
81.3
81.4
81.5
Position on chr4 (Mb)

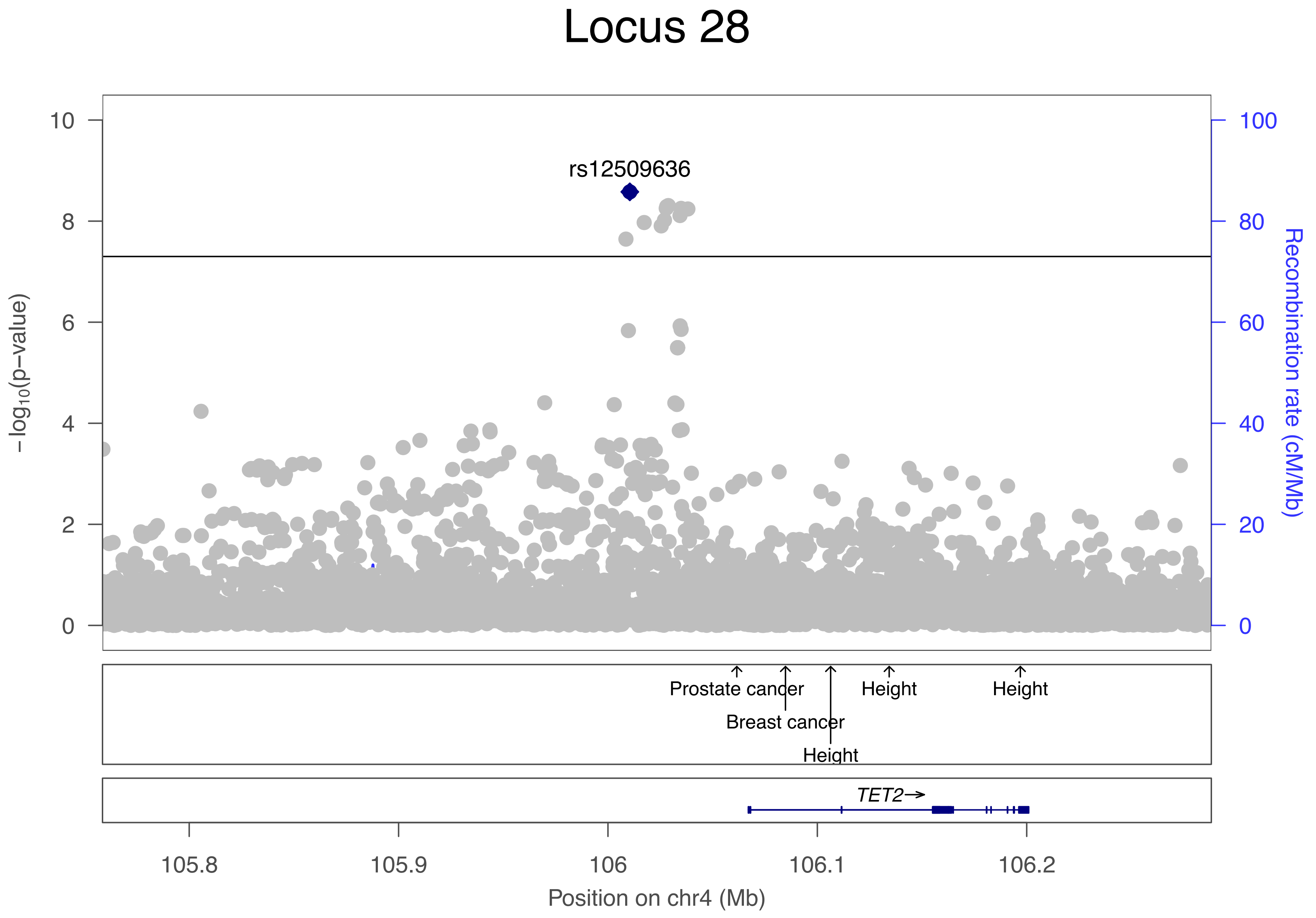

Locus 28
rs12509636
-log10(p-value)
Recombination rate (cM/Mb)
10
8
6
4
2
0
100
80
60
40
20
0
Prostate cancer
Breast cancer
Height
Height
Height
TET2→
105.8
105.9
106
106.1
106.2
Position on chr4 (Mb)

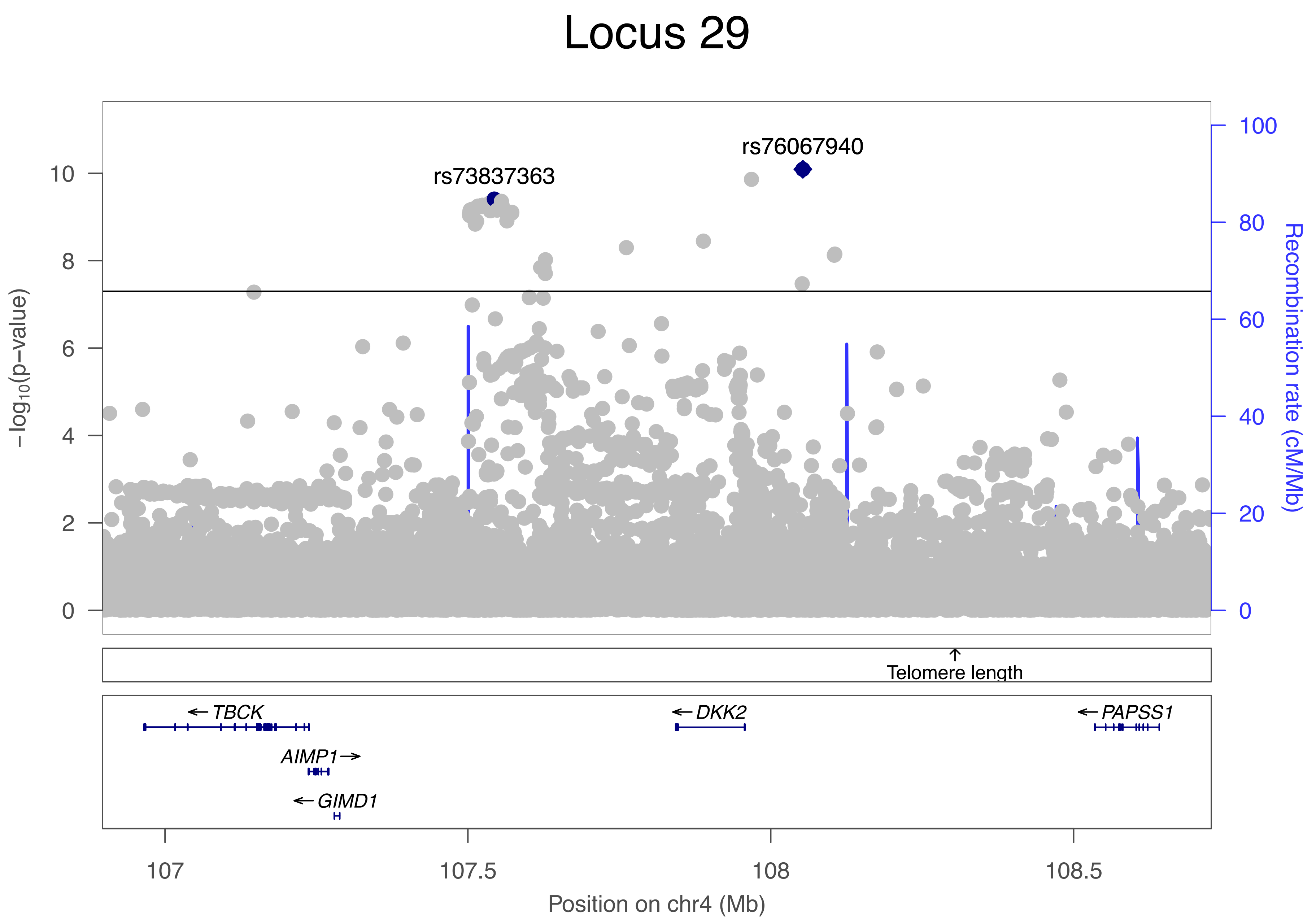

Locus 29
rs76067940
rs73837363
−log10(p−value)
Recombination rate (cM/Mb)
0
2
4
6
8
10
0
20
40
60
80
100
Telomere length
TBCK
DKK2
PAPSS1
AIMP1
GIMD1
107
107.5
108
108.5
Position on chr4 (Mb)

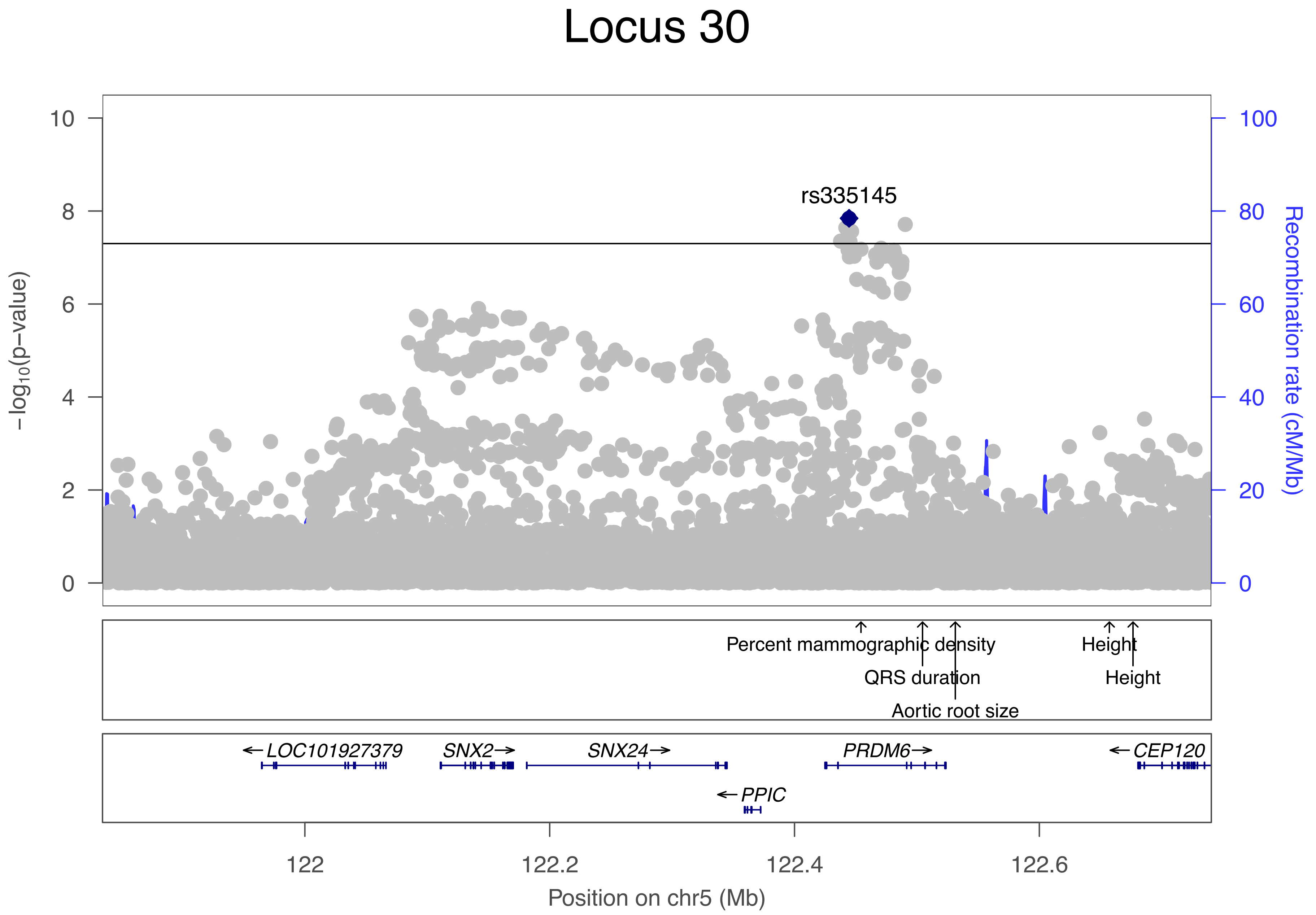

Locus 30
rs335145
−log10(p−value)
Recombination rate (cM/Mb)
0
2
4
6
8
10
0
20
40
60
80
100
Percent mammographic density
QRS duration
Aortic root size
Height
Height
LOC101927379
SNX2
SNX24
PPIC
PRDM6
CEP120
122
122.2
122.4
122.6
Position on chr5 (Mb)

Locus 31

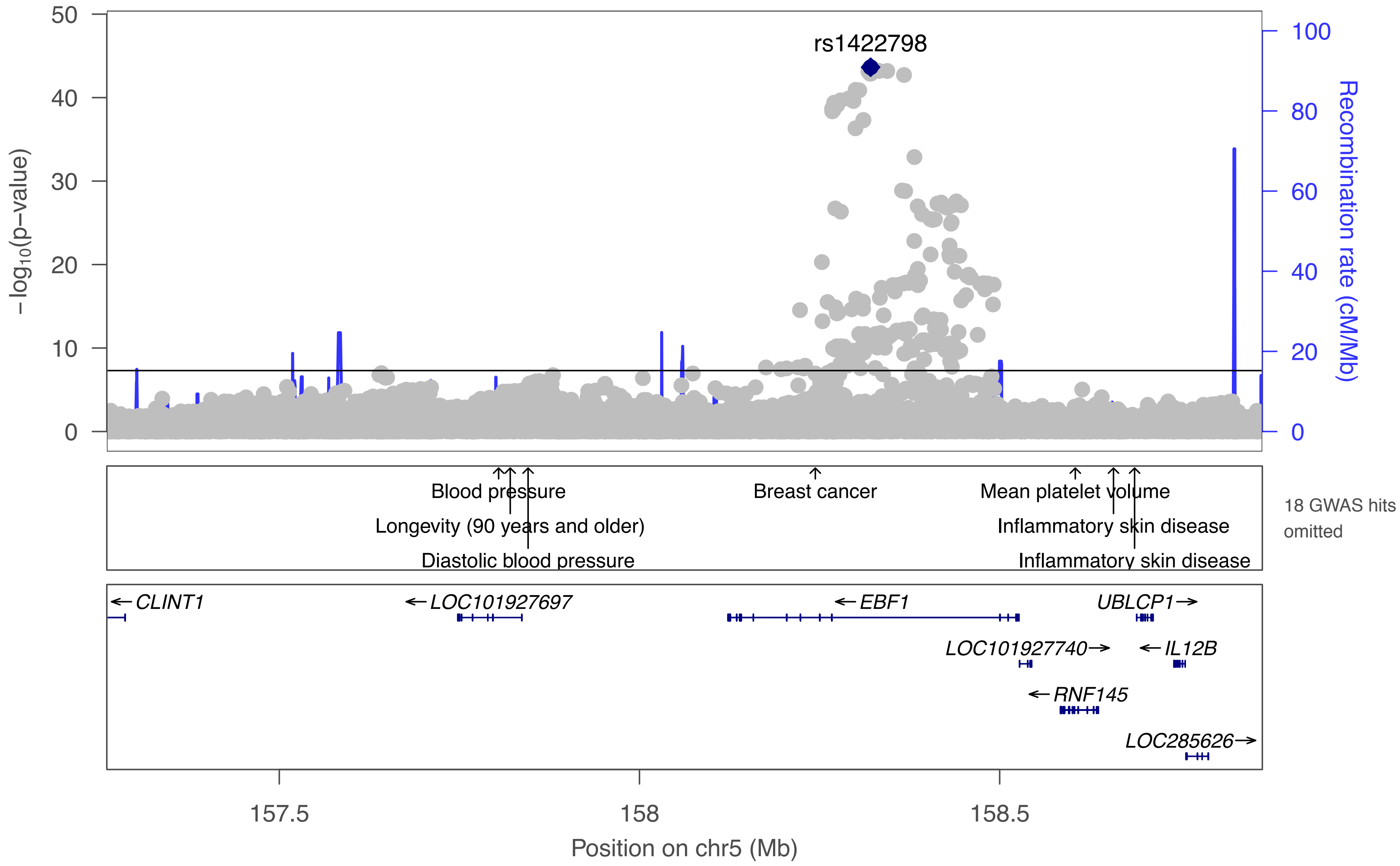

rs1422798
−log10(p−value)
Recombination rate (cM/Mb)
Blood pressure
Longevity (90 years and older)
Diastolic blood pressure
Breast cancer
Mean platelet volume
Inflammatory skin disease
Inflammatory skin disease
18 GWAS hits omitted
CLINT1
LOC101927697
EBF1
UBLCP1
LOC101927740
IL12B
RNF145
LOC285626
157.5
158
158.5
Position on chr5 (Mb)

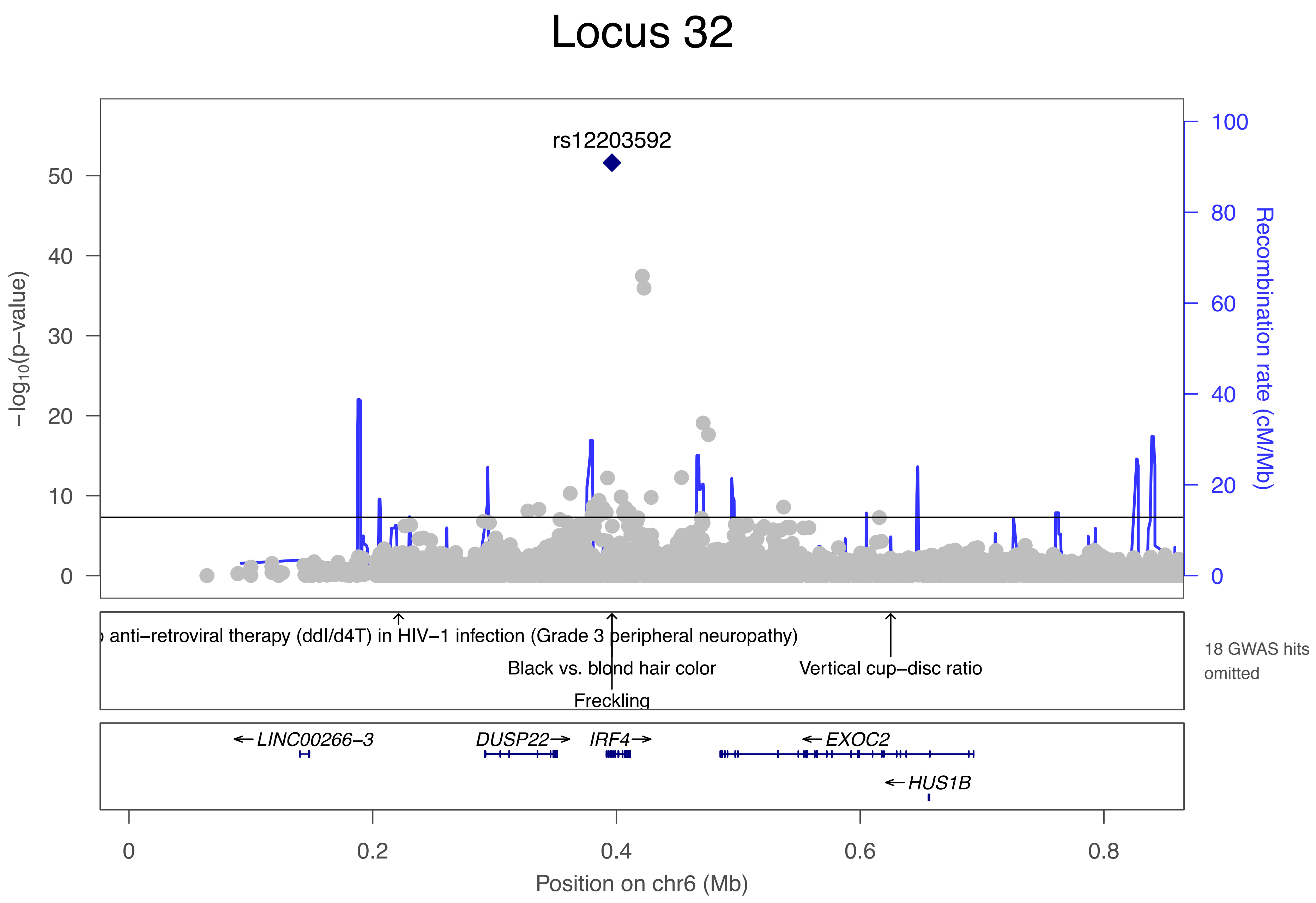

Locus 32
rs12203592
−log10(p−value)
50
40
30
20
10
0
100
80
60
40
20
0
Recombination rate (cM/Mb)
anti−retroviral therapy (ddI/d4T) in HIV−1 infection (Grade 3 peripheral neuropathy)
Black vs. blond hair color
Freckling
Vertical cup−disc ratio
18 GWAS hits omitted
← LINC00266−3
DUSP22 →
IRF4 →
← EXOC2
← HUS1B
0
0.2
0.4
0.6
0.8
Position on chr6 (Mb)

Locus 33

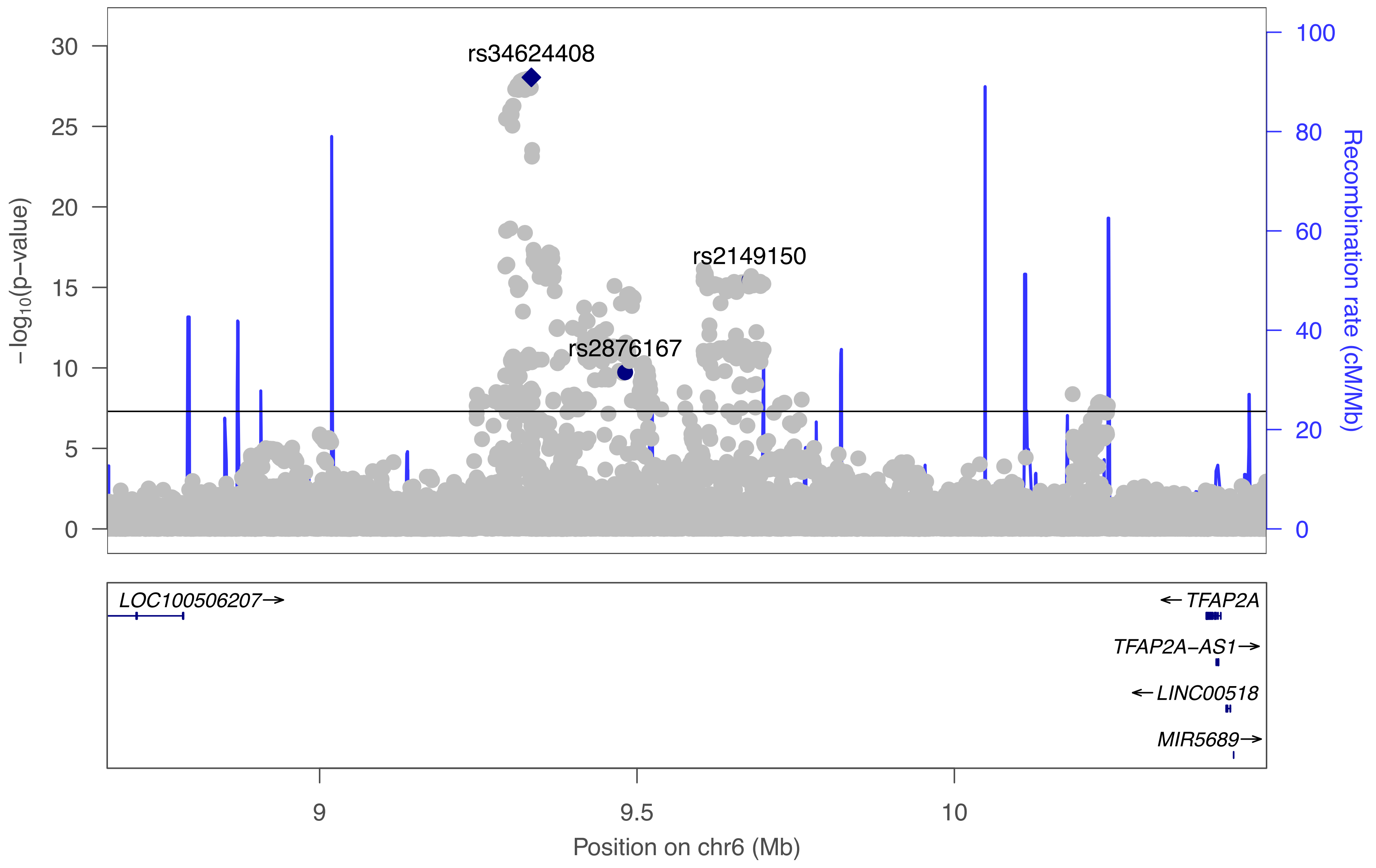

rs34624408
rs2149150
rs2876167
−log10(p−value)
Recombination rate (cM/Mb)
30
25
20
15
10
5
0
100
80
60
40
20
0
LOC100506207→
←TFAP2A
TFAP2A−AS1→
←LINC00518
MIR5689→
9
9.5
10
Position on chr6 (Mb)

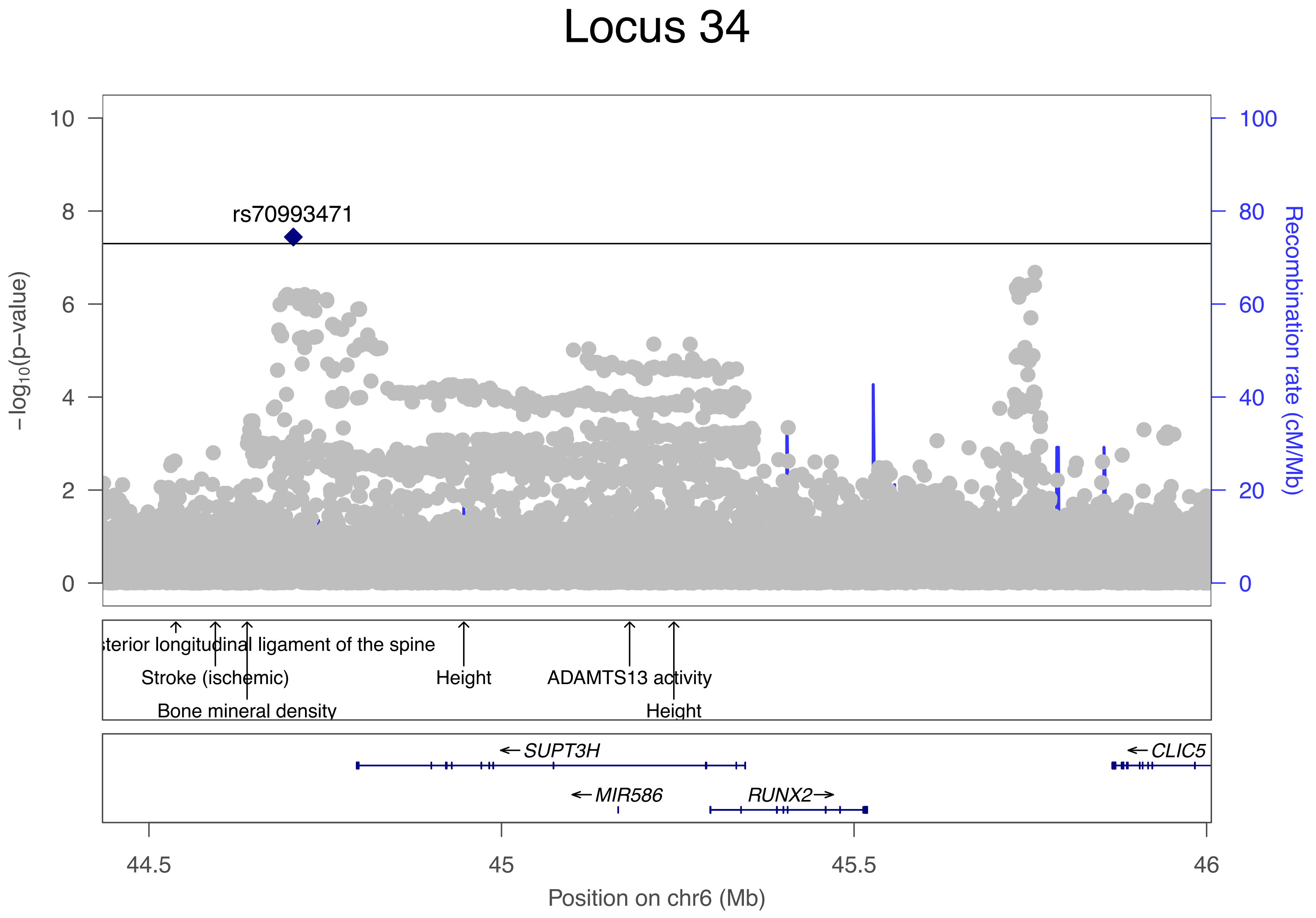

Locus 34
rs70993471
−log10(p−value)
Recombination rate (cM/Mb)
10
8
6
4
2
0
100
80
60
40
20
0
sterior longitudinal ligament of the spine
Stroke (ischemic)
Bone mineral density
Height
ADAMTS13 activity
Height
← SUPT3H
← CLIC5
← MIR586
RUNX2 →
44.5
45
45.5
46
Position on chr6 (Mb)

Locus 35

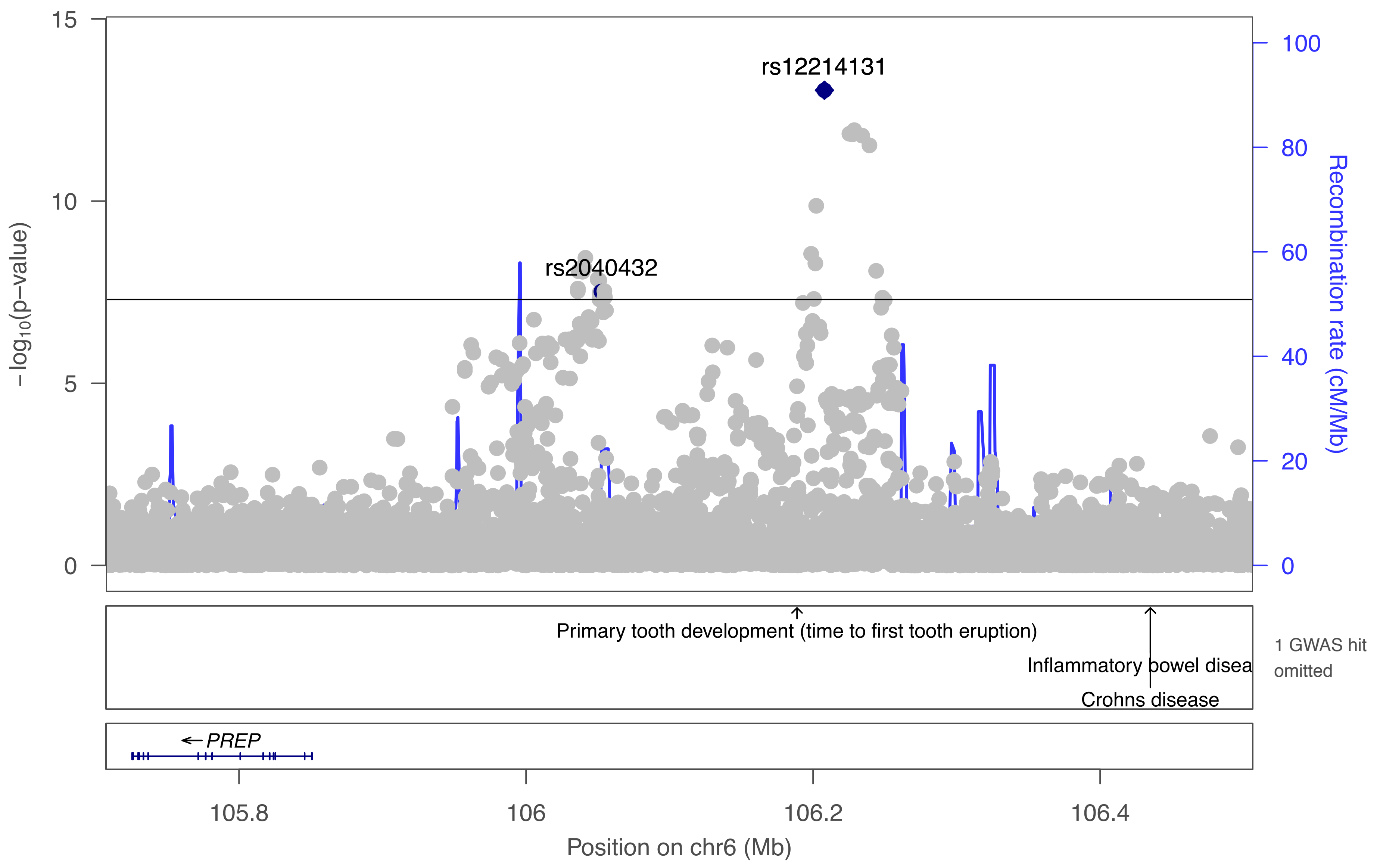

rs12214131
rs2040432
15
10
5
0
100
80
60
40
20
0
−log₁₀(p−value)
Recombination rate (cM/Mb)
Primary tooth development (time to first tooth eruption)
Inflammatory bowel disea
Crohns disease
1 GWAS hit
omitted
← PREP
105.8
106
106.2
106.4
Position on chr6 (Mb)

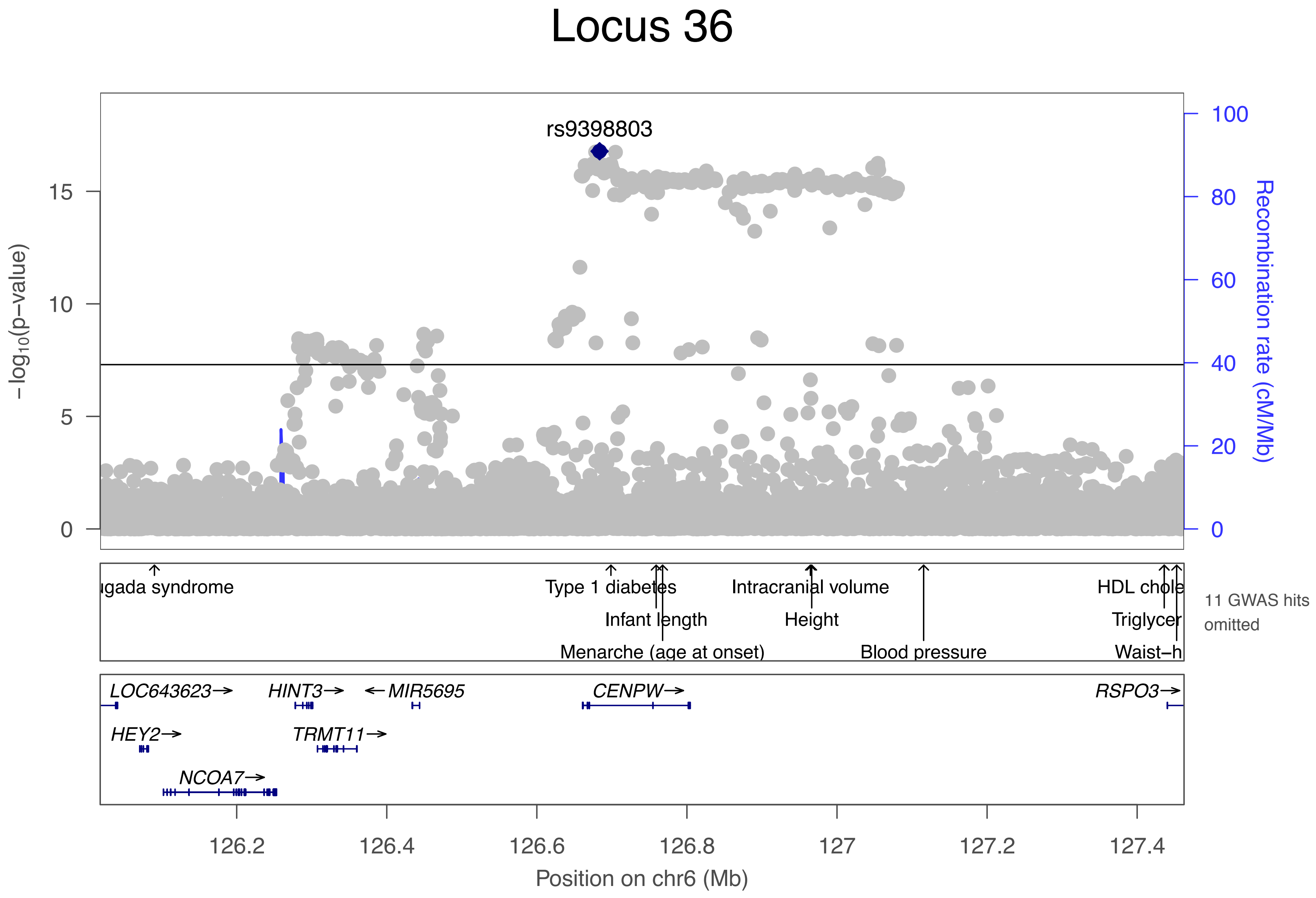

Locus 36
rs9398803
−log10(p−value)
Recombination rate (cM/Mb)
0
5
10
15
0
20
40
60
80
100
ıgada syndrome
Type 1 diabetes
Infant length
Menarche (age at onset)
Intracranial volume
Height
Blood pressure
HDL chole
Triglycer
Waist−h
11 GWAS hits omitted
LOC643623→
HEY2→
NCOA7→
HINT3→
TRMT11→
←MIR5695
CENPW→
RSPO3→
126.2
126.4
126.6
126.8
127
127.2
127.4
Position on chr6 (Mb)

Locus 37

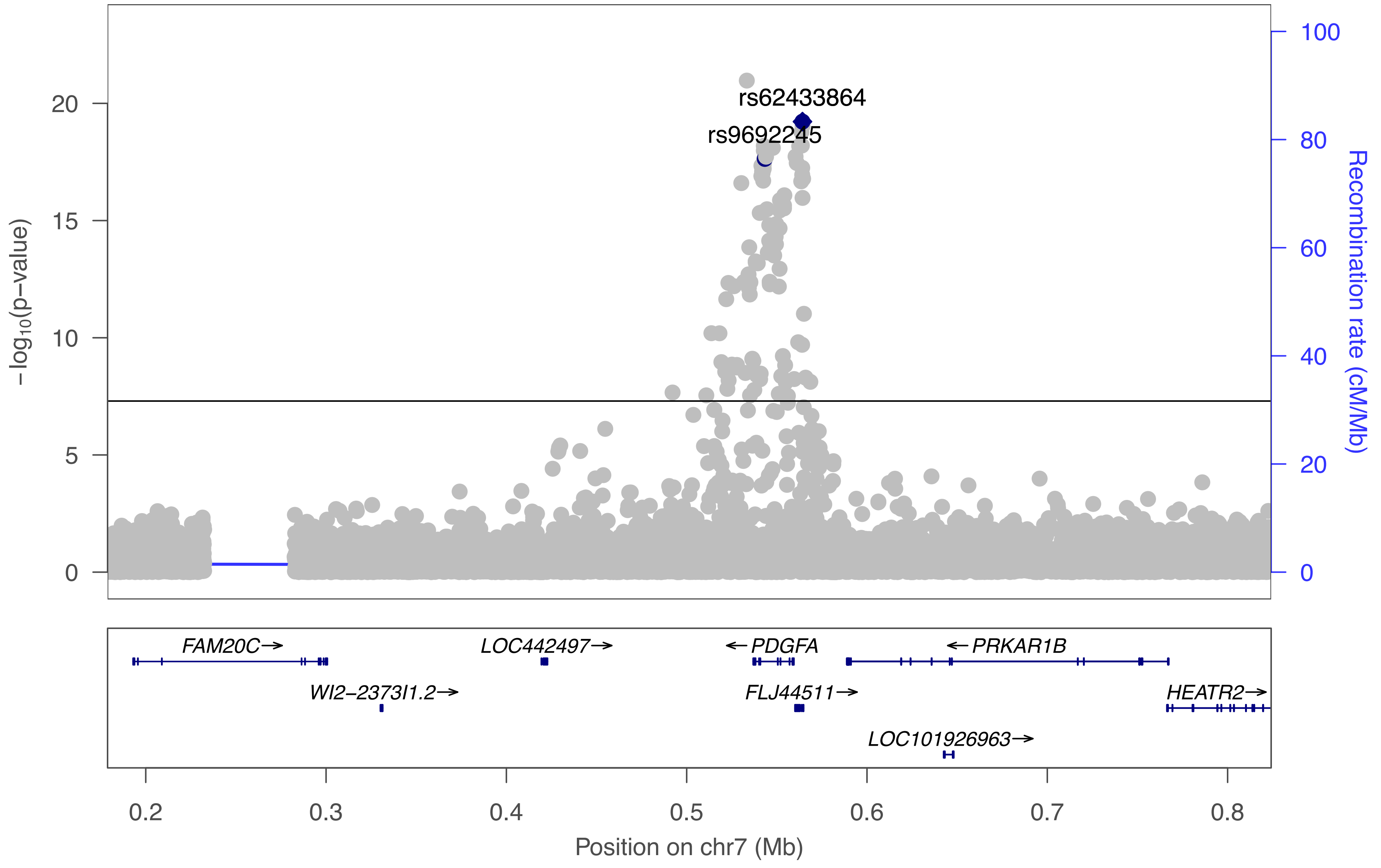

rs62433864
rs9692245
-log10(p-value)
20
15
10
5
0
Recombination rate (cM/Mb)
100
80
60
40
20
0
FAM20C→
LOC442497→
←PDGFA
←PRKAR1B
WI2-2373I1.2→
FLJ44511→
HEATR2→
LOC101926963→
0.2
0.3
0.4
0.5
0.6
0.7
0.8
Position on chr7 (Mb)

Locus 38

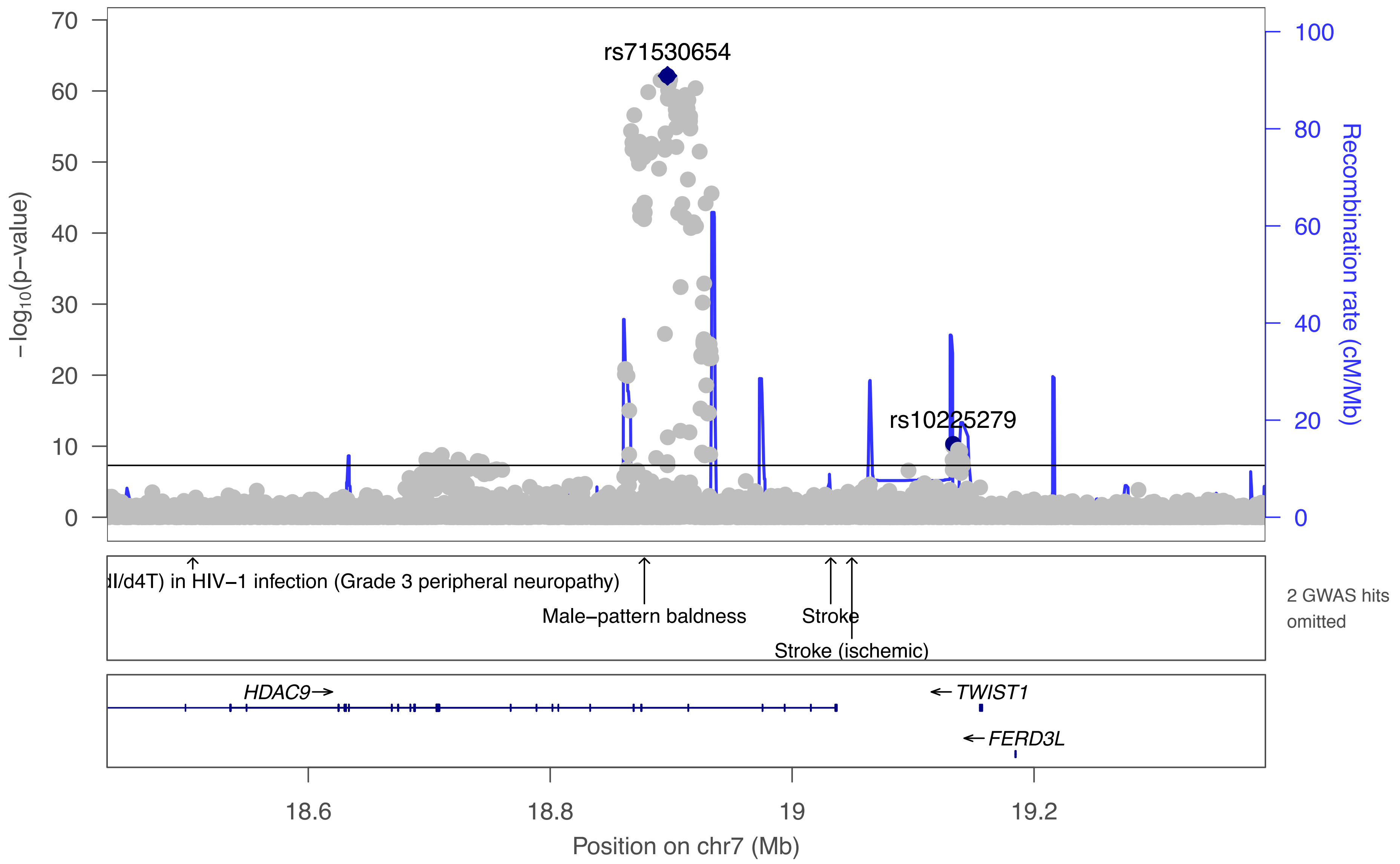

rs71530654
rs10225279
−log10(p−value)
Recombination rate (cM/Mb)
dl/d4T) in HIV−1 infection (Grade 3 peripheral neuropathy)
Male−pattern baldness
Stroke
Stroke (ischemic)
2 GWAS hits omitted
HDAC9→
← TWIST1
← FERD3L
18.6
18.8
19
19.2
Position on chr7 (Mb)

Locus 39

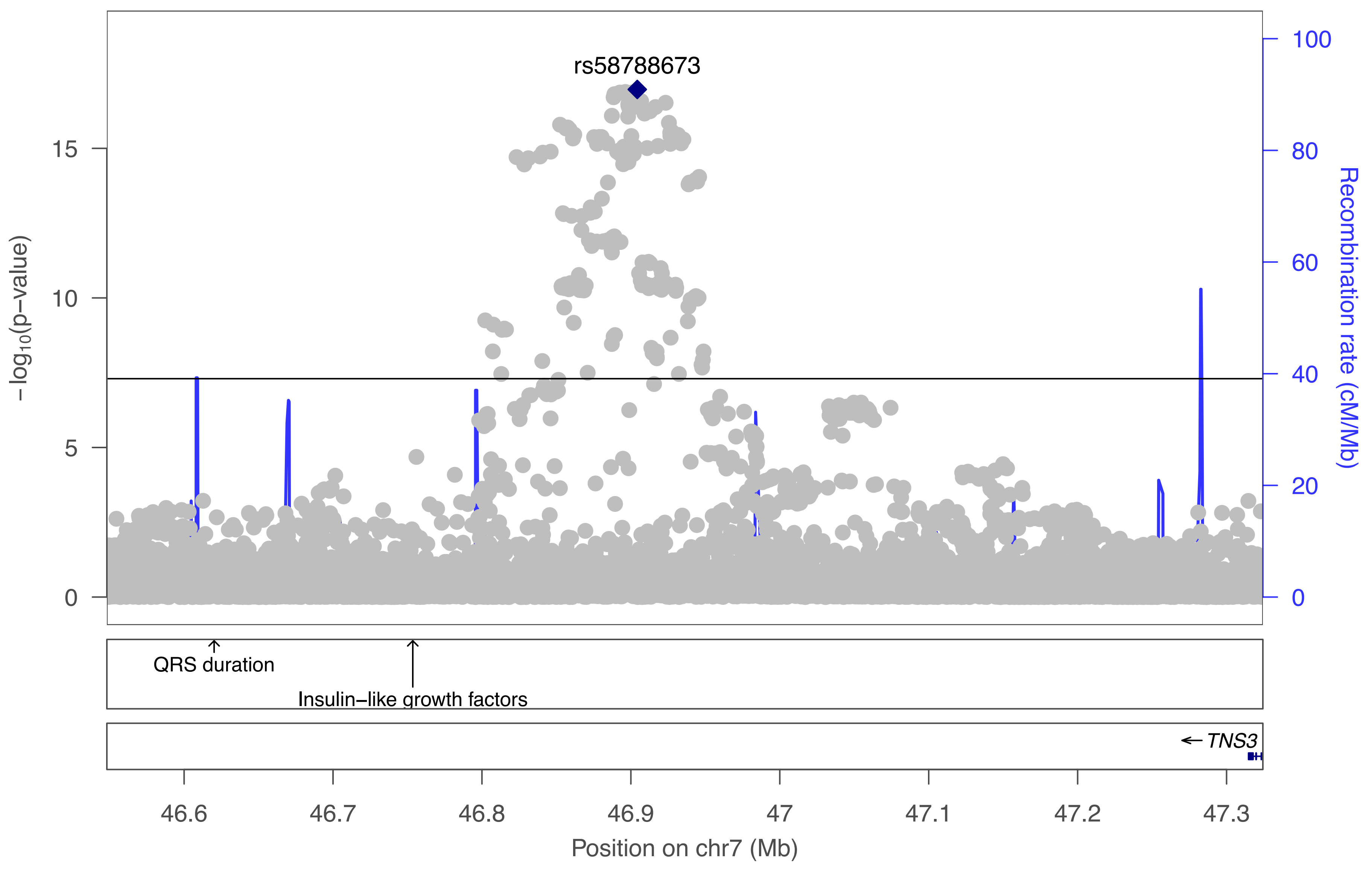

rs58788673
−log10(p−value)
Recombination rate (cM/Mb)
15
10
5
0
100
80
60
40
20
0
QRS duration
Insulin−like growth factors
← TNS3
46.6
46.7
46.8
46.9
47
47.1
47.2
47.3
Position on chr7 (Mb)

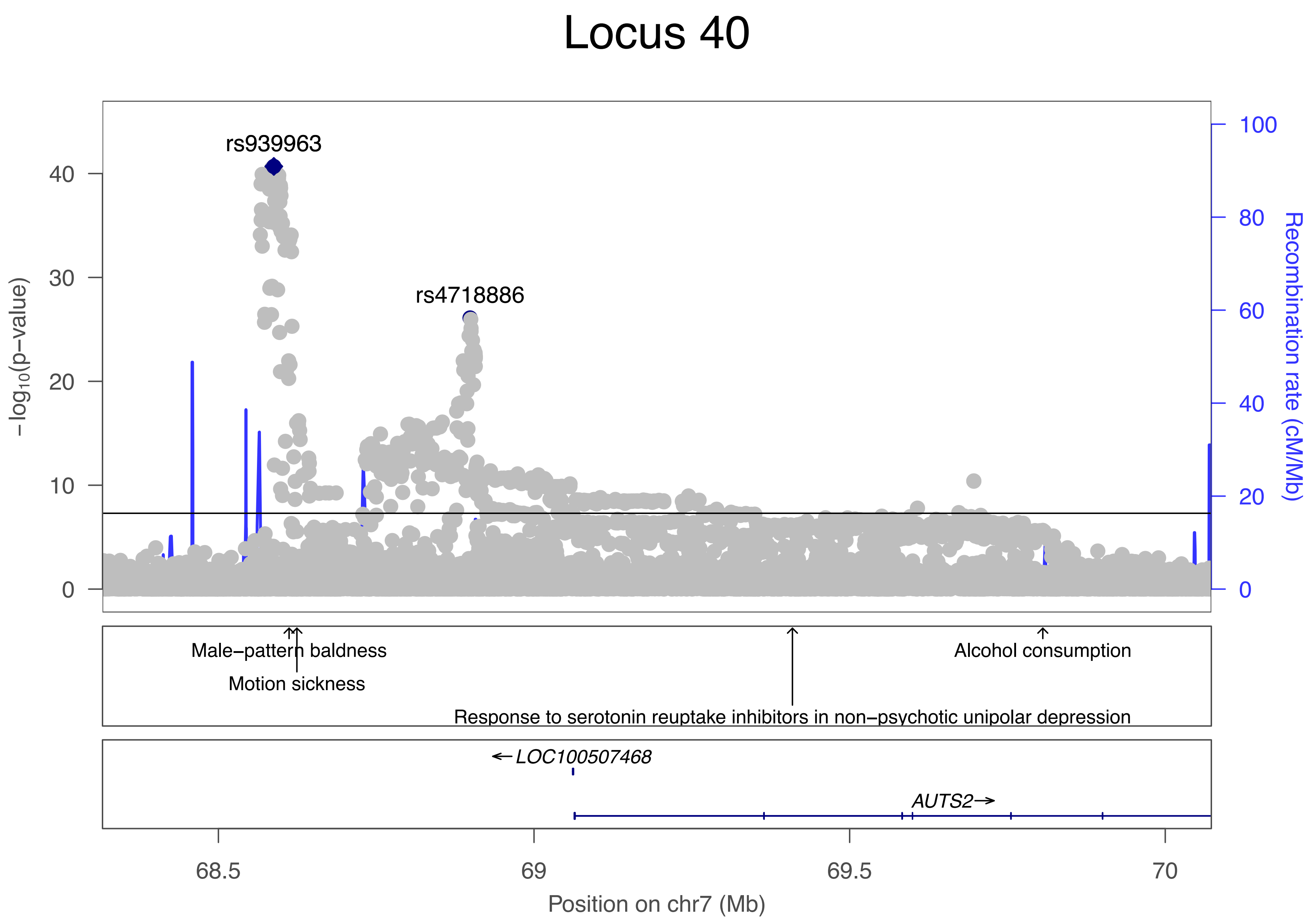
Locus 40
rs939963
rs4718886
40
30
20
10
0
$-\log_{10}$(p−value)
100
80
60
40
20
0
Recombination rate (cM/Mb)
Male−pattern baldness
Motion sickness
Alcohol consumption
Response to serotonin reuptake inhibitors in non−psychotic unipolar depression
←LOC100507468
AUTS2→
68.5
69
69.5
70
Position on chr7 (Mb)

Locus 41

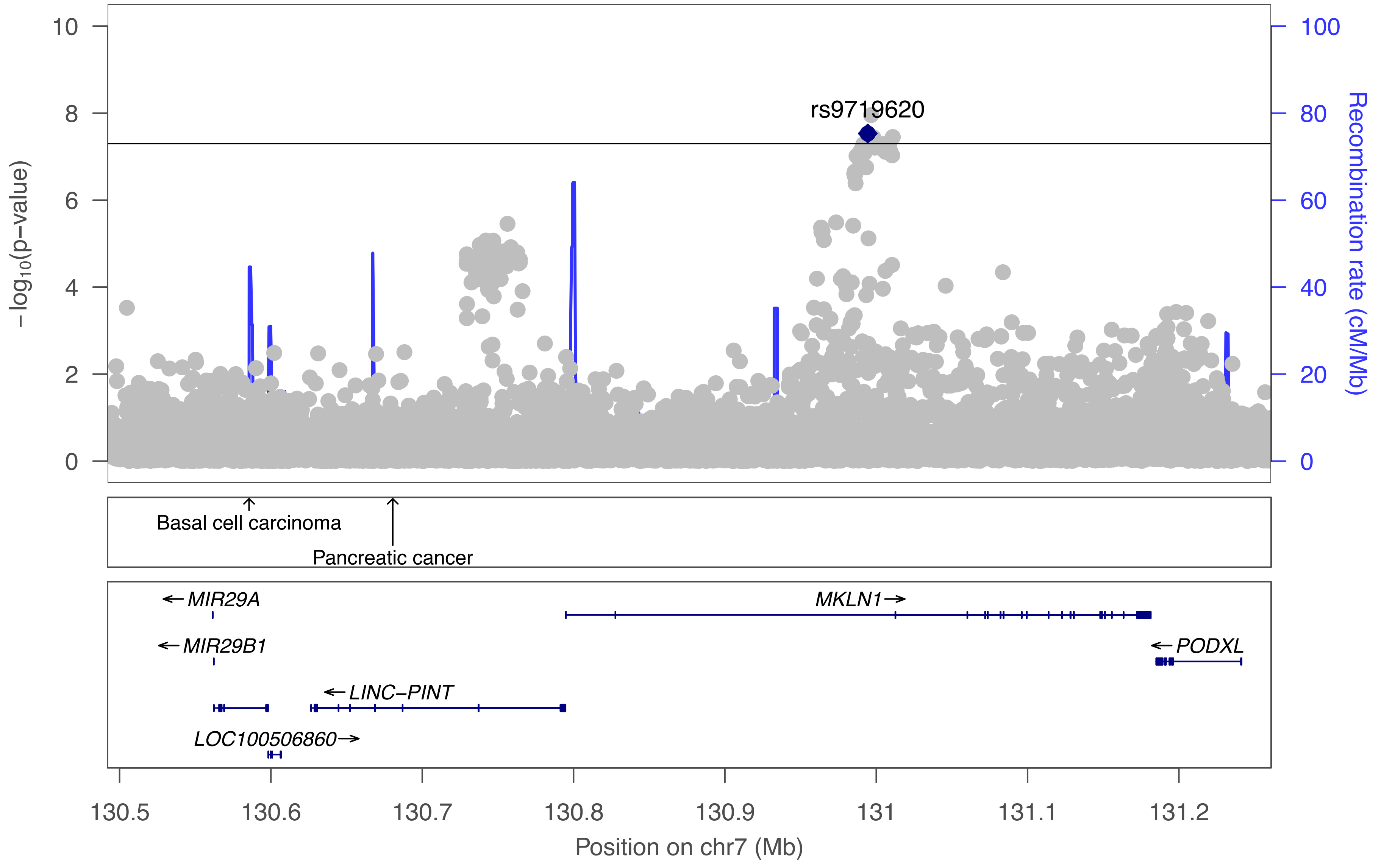
rs9719620
-log10(p-value)
Recombination rate (cM/Mb)
Basal cell carcinoma
Pancreatic cancer
MIR29A
MIR29B1
LINC-PINT
LOC100506860
MKLN1
PODXL
130.5
130.6
130.7
130.8
130.9
131
131.1
131.2
Position on chr7 (Mb)

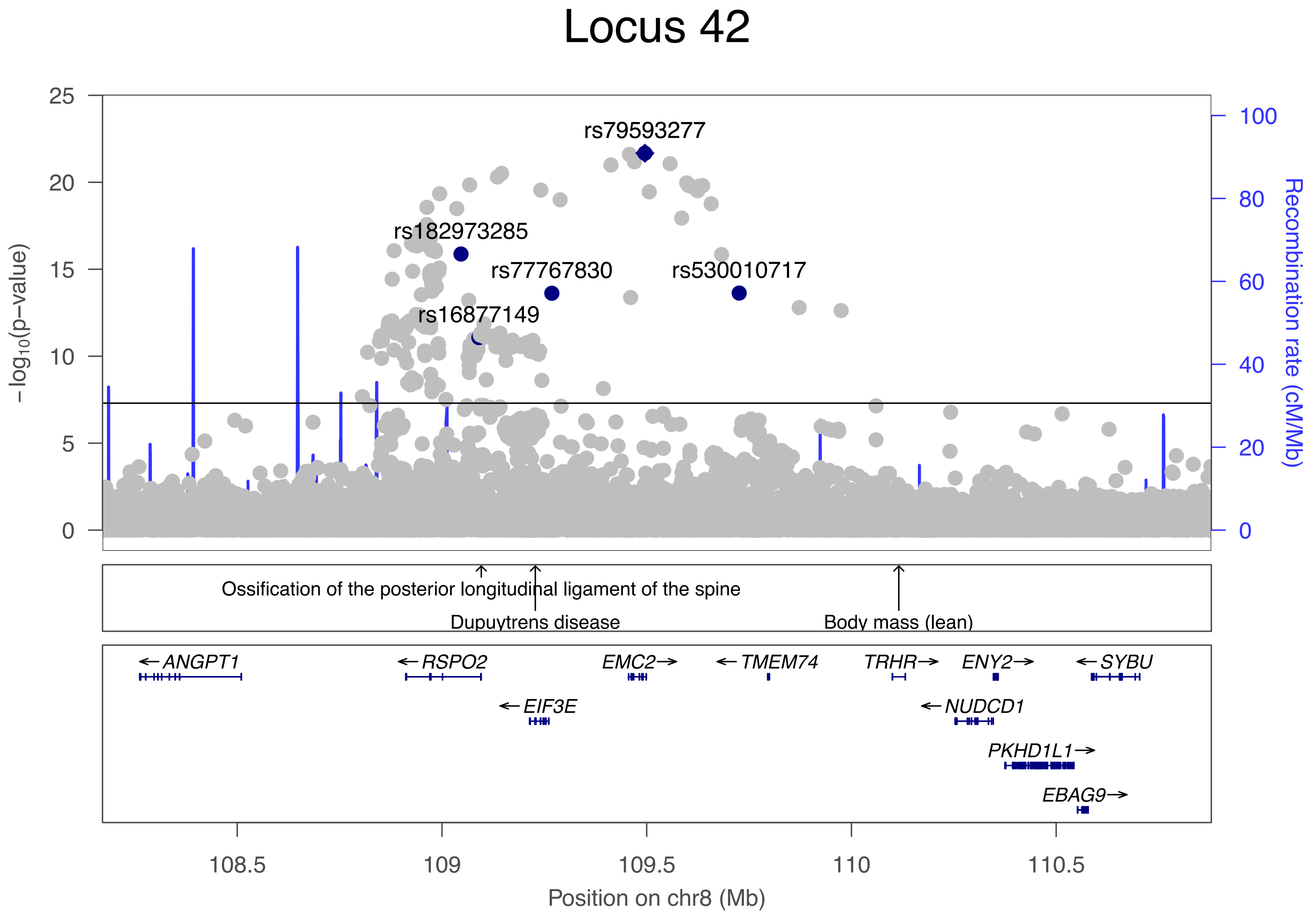

Locus 42
rs79593277
rs182973285
rs77767830
rs530010717
rs16877149
25
20
15
10
5
0
−log10(p−value)
100
80
60
40
20
0
Recombination rate (cM/Mb)
Ossification of the posterior longitudinal ligament of the spine
Dupuytrens disease
Body mass (lean)
← ANGPT1
← RSPO2
EMC2→
← TMEM74
TRHR→
ENY2→
← SYBU
← EIF3E
← NUDCD1
PKHD1L1→
EBAG9→
108.5
109
109.5
110
110.5
Position on chr8 (Mb)

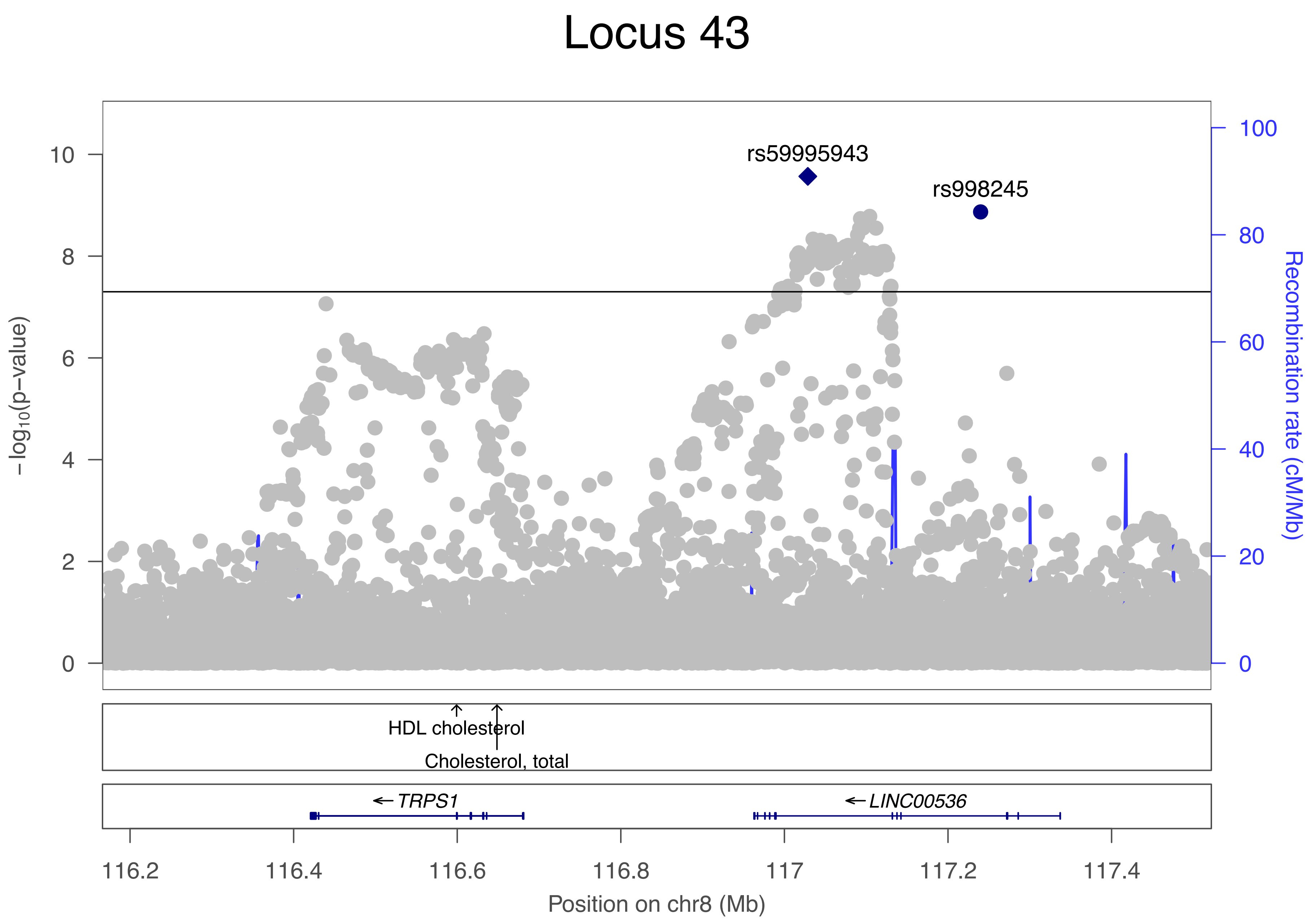
Locus 43
rs59995943
rs998245
−log10(p−value)
Recombination rate (cM/Mb)
0
2
4
6
8
10
0
20
40
60
80
100
HDL cholesterol
Cholesterol, total
TRPS1
LINC00536
116.2
116.4
116.6
116.8
117
117.2
117.4
Position on chr8 (Mb)

Locus 44

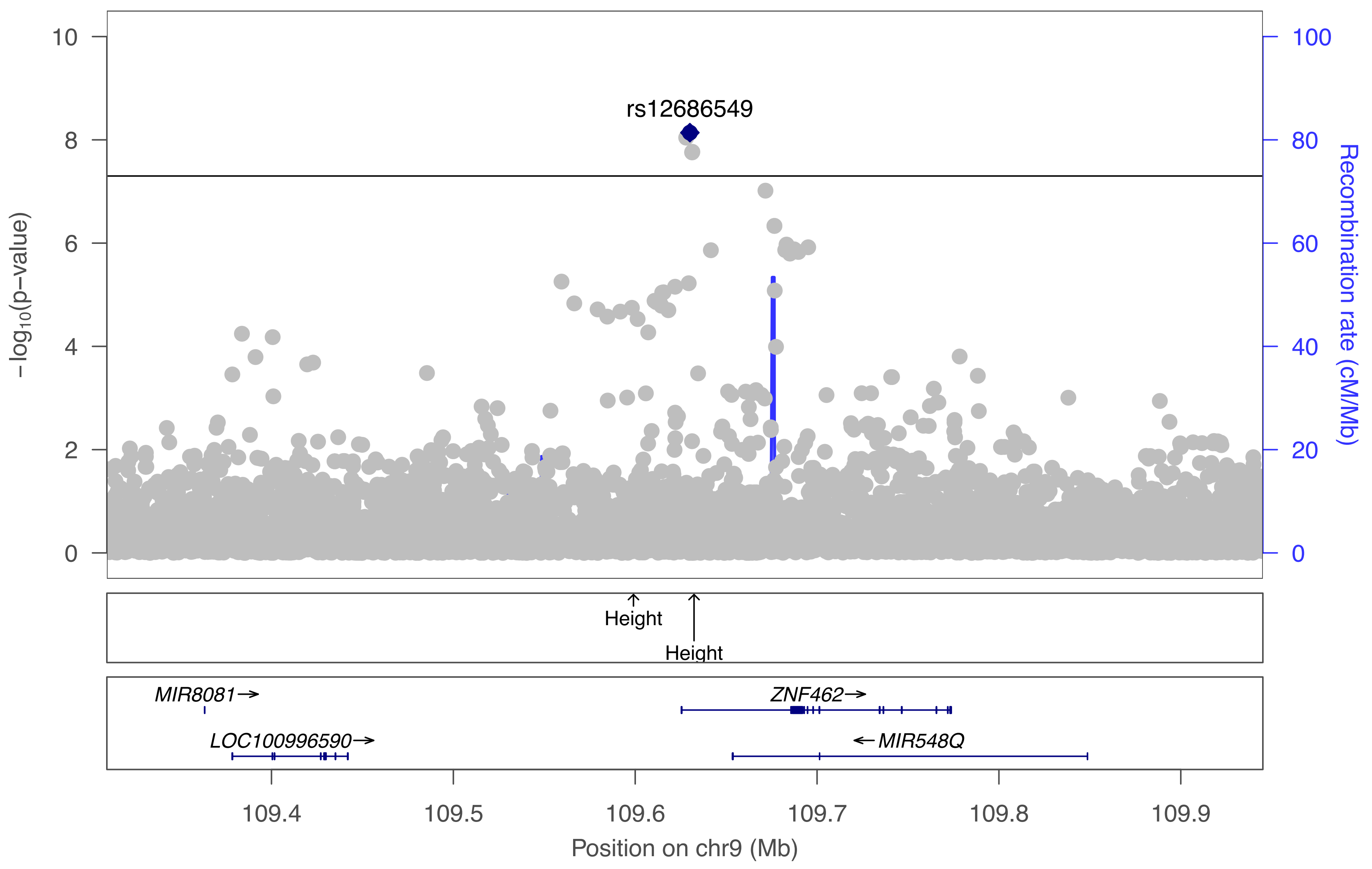
rs12686549
-log10(p-value)
Recombination rate (cM/Mb)
10
8
6
4
2
0
100
80
60
40
20
0
Height
Height
MIR8081→
LOC100996590→
ZNF462→
←MIR548Q
109.4
109.5
109.6
109.7
109.8
109.9
Position on chr9 (Mb)

Locus 45

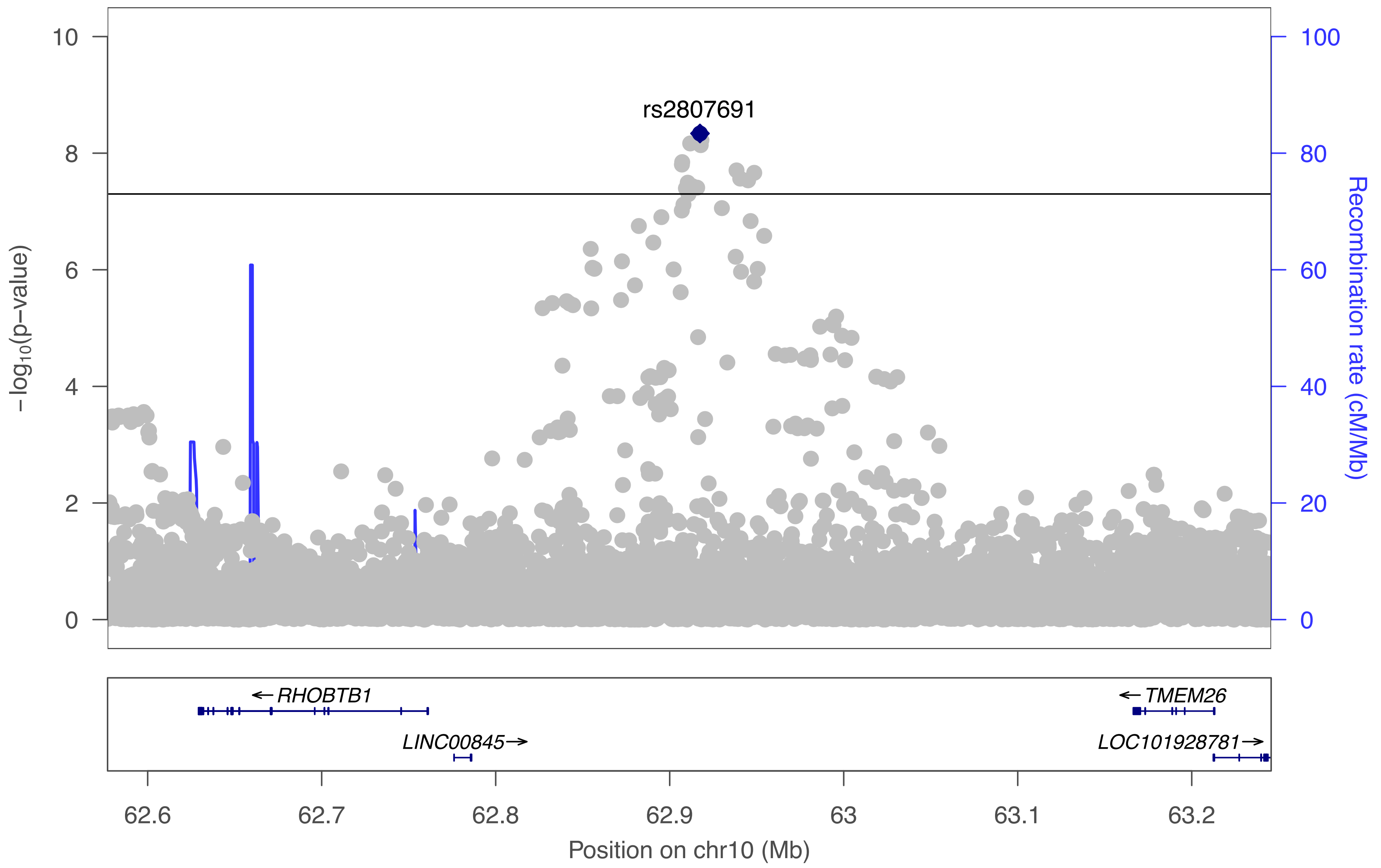

rs2807691
−log10(p−value)
Recombination rate (cM/Mb)
10
8
6
4
2
0
100
80
60
40
20
0
← RHOBTB1
LINC00845→
← TMEM26
LOC101928781→
62.6
62.7
62.8
62.9
63
63.1
63.2
Position on chr10 (Mb)

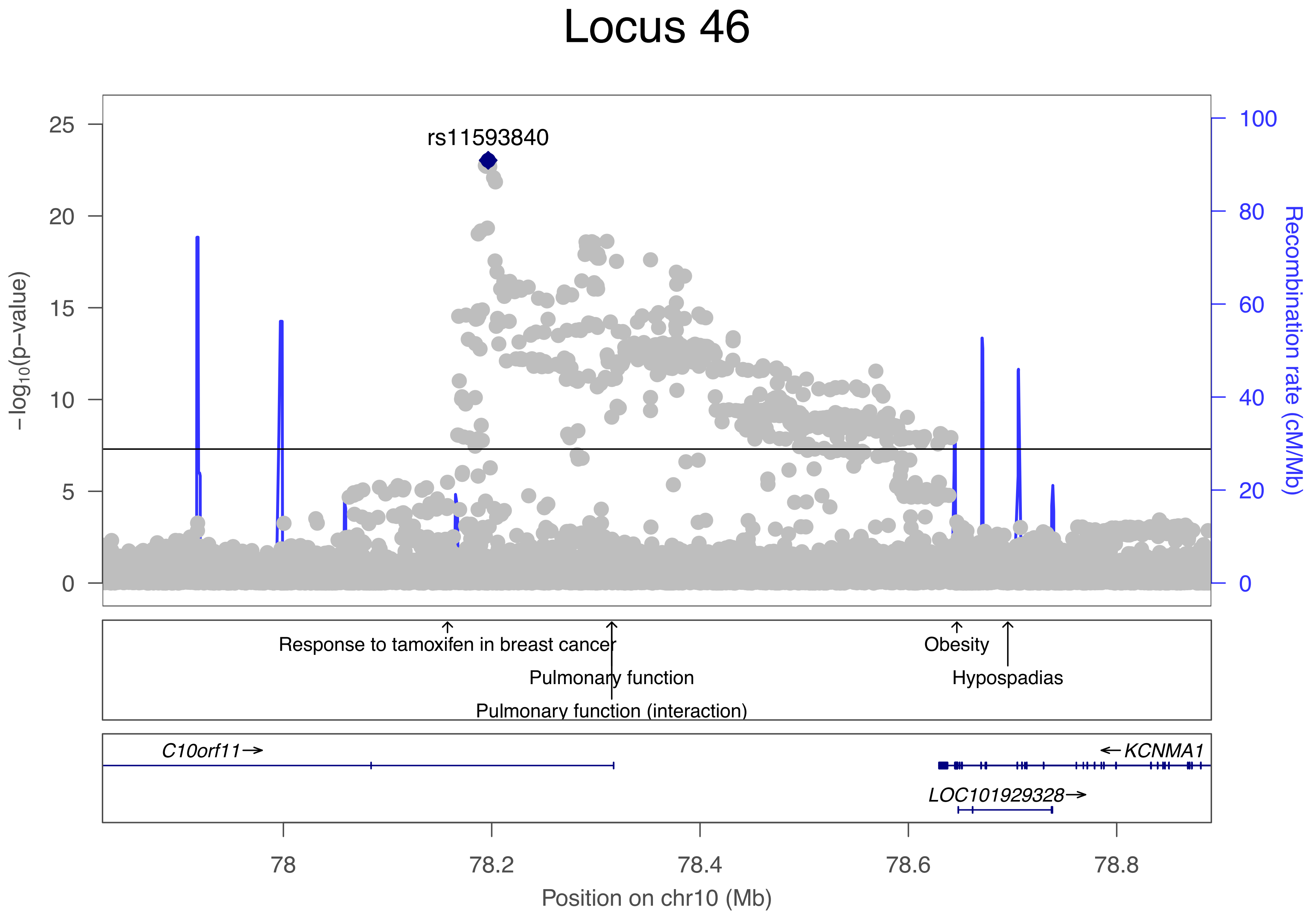
Locus 46
rs11593840
−log10(p−value)
Recombination rate (cM/Mb)
Response to tamoxifen in breast cancer
Pulmonary function
Pulmonary function (interaction)
Obesity
Hypospadias
C10orf11→
←KCNMA1
LOC101929328→
Position on chr10 (Mb)

Locus 47

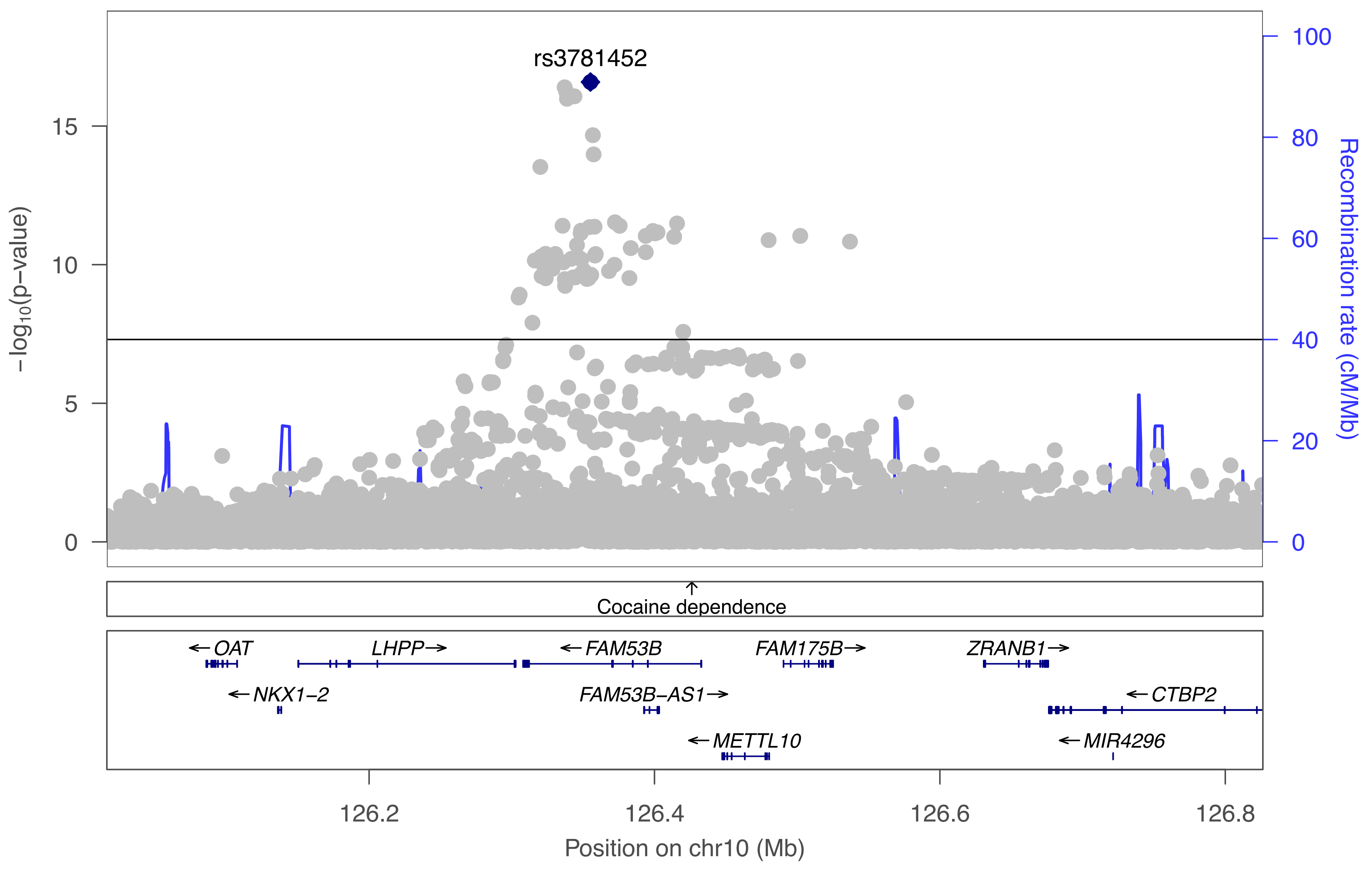

rs3781452
−log10(p−value)
15
10
5
0
100
80
60
40
20
0
Recombination rate (cM/Mb)
Cocaine dependence
←OAT
LHPP→
←FAM53B
FAM175B→
ZRANB1→
←NKX1−2
FAM53B−AS1→
←CTBP2
←METTL10
←MIR4296
126.2
126.4
126.6
126.8
Position on chr10 (Mb)

Locus 48

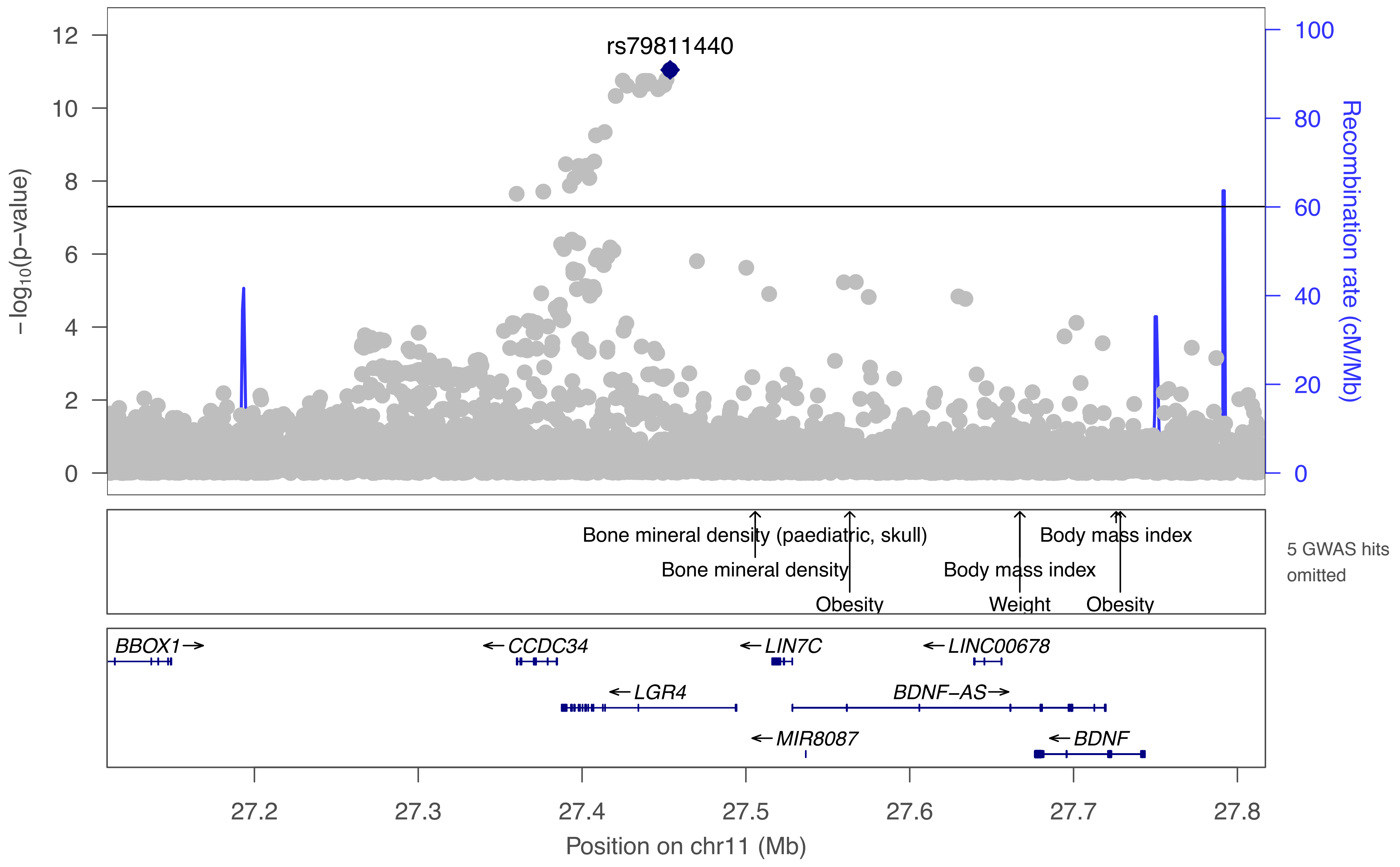

rs79811440
−log10(p−value)
Recombination rate (cM/Mb)
Bone mineral density (paediatric, skull)
Bone mineral density
Obesity
Body mass index
Body mass index
Weight
Obesity
5 GWAS hits omitted
BBOX1
CCDC34
LIN7C
LINC00678
LGR4
BDNF−AS
MIR8087
BDNF
27.2
27.3
27.4
27.5
27.6
27.7
27.8
Position on chr11 (Mb)

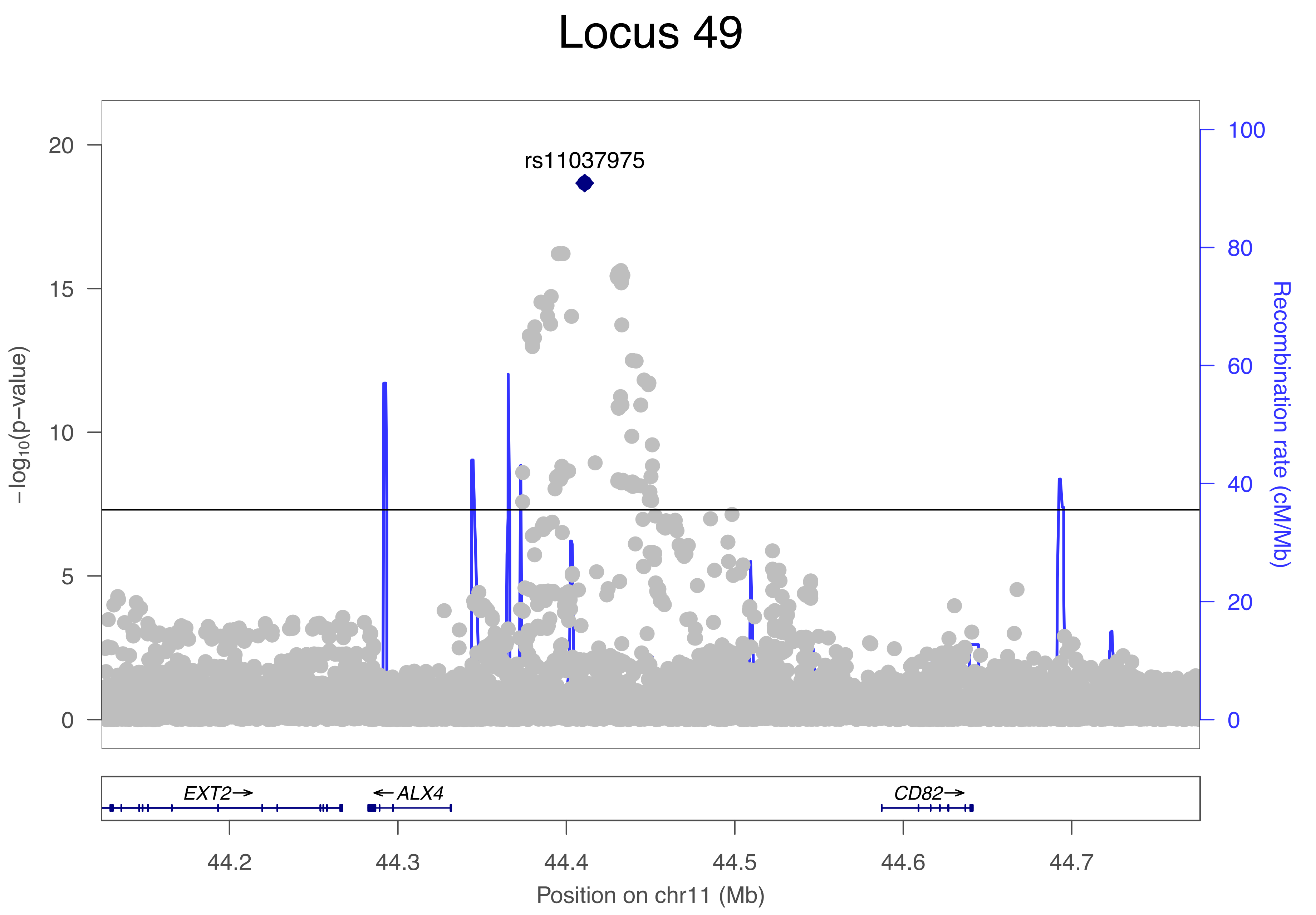
Locus 49
rs11037975
−log10(p−value)
20
15
10
5
0
Recombination rate (cM/Mb)
100
80
60
40
20
0
EXT2→
←ALX4
CD82→
44.2
44.3
44.4
44.5
44.6
44.7
Position on chr11 (Mb)

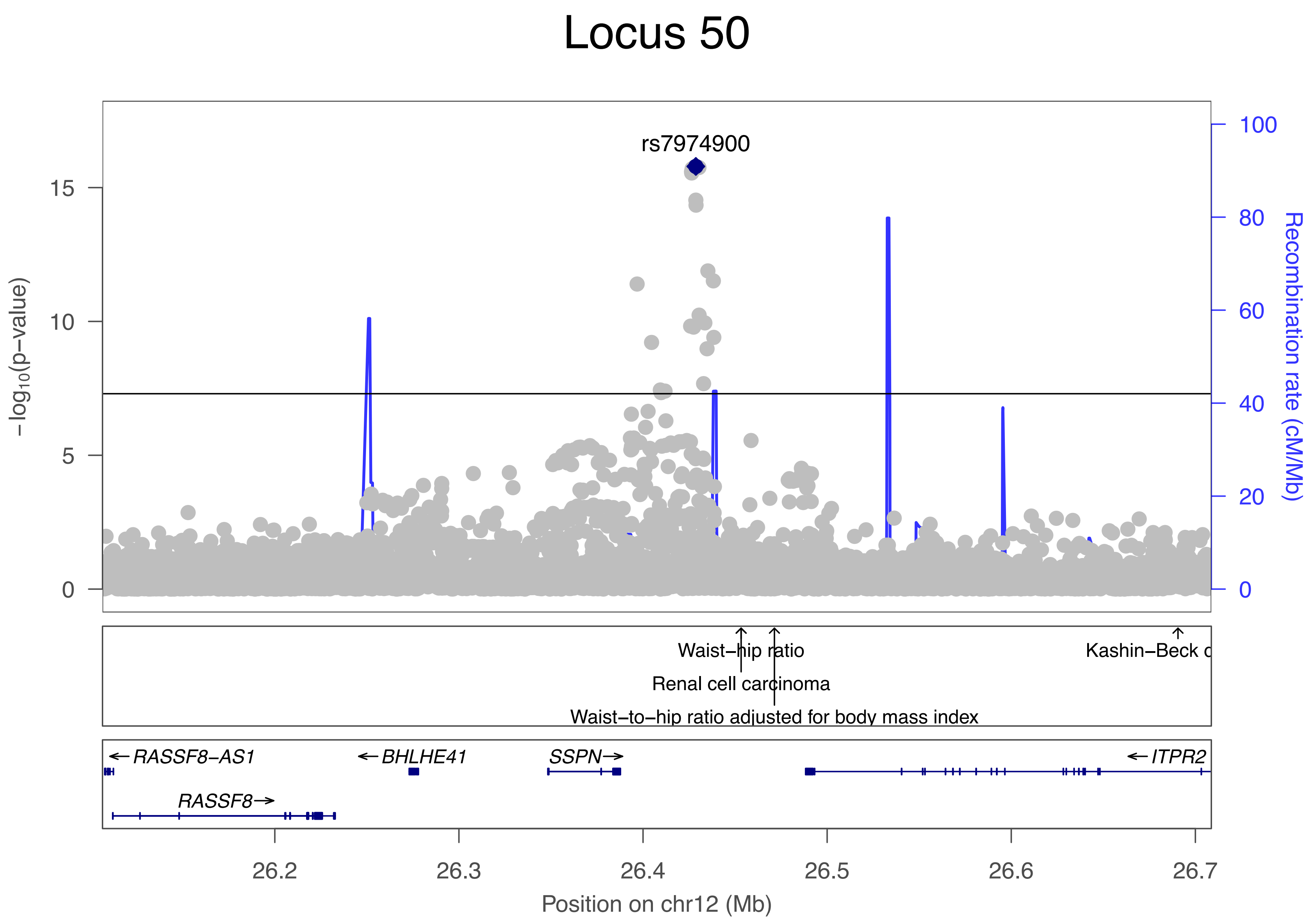

Locus 50
rs7974900
15
10
5
0
−log10(p−value)
100
80
60
40
20
0
Recombination rate (cM/Mb)
Waist−hip ratio
Renal cell carcinoma
Waist−to−hip ratio adjusted for body mass index
Kashin−Beck d
← RASSF8−AS1
← BHLHE41
SSPN→
← ITPR2
RASSF8→
26.2
26.3
26.4
26.5
26.6
26.7
Position on chr12 (Mb)

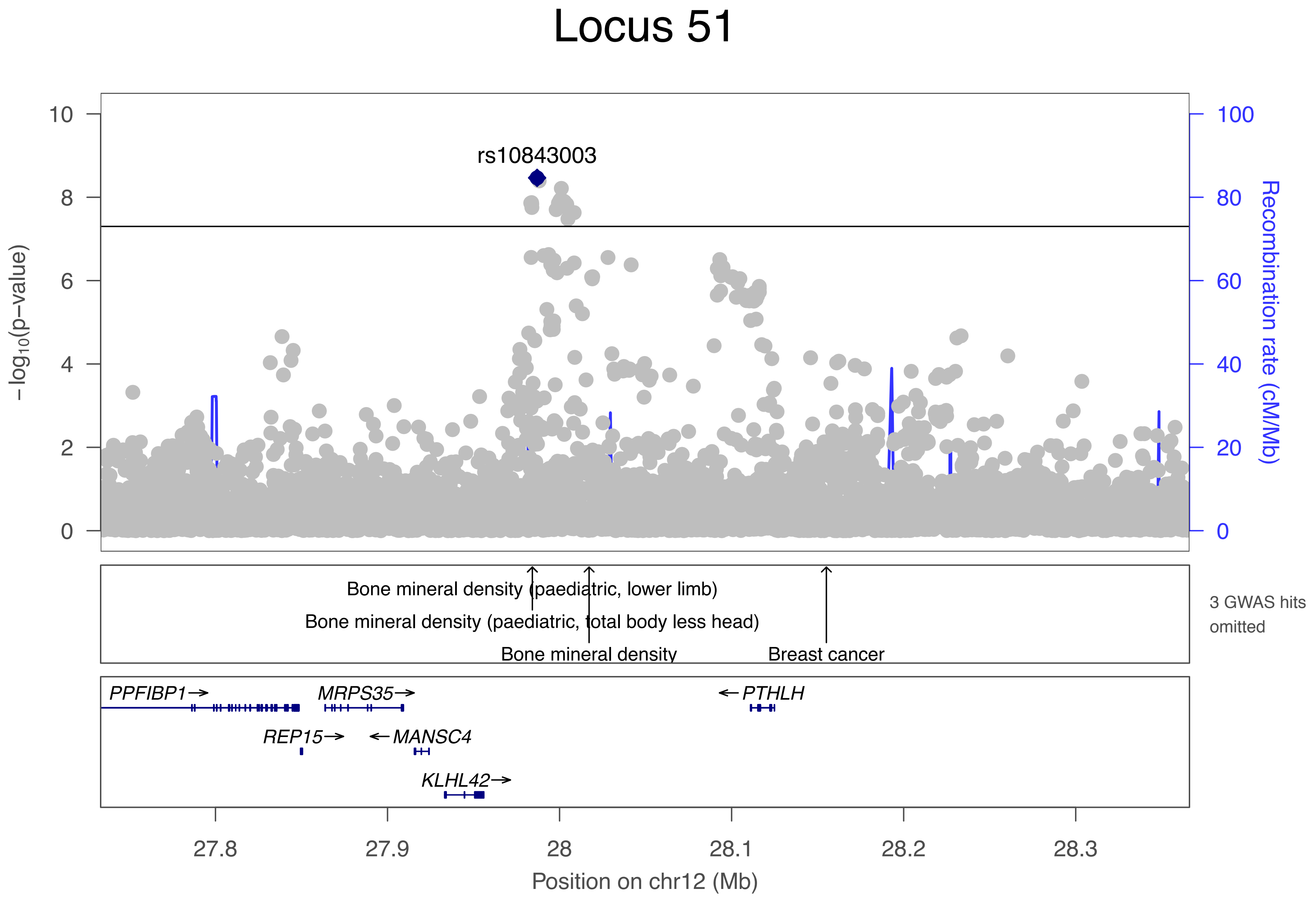
Locus 51
rs10843003
−log10(p−value)
Recombination rate (cM/Mb)
10
8
6
4
2
0
100
80
60
40
20
0
Bone mineral density (paediatric, lower limb)
Bone mineral density (paediatric, total body less head)
Bone mineral density
Breast cancer
3 GWAS hits omitted
PPFIBP1→
MRPS35→
←PTHLH
REP15→
←MANSC4
KLHL42→
27.8
27.9
28
28.1
28.2
28.3
Position on chr12 (Mb)

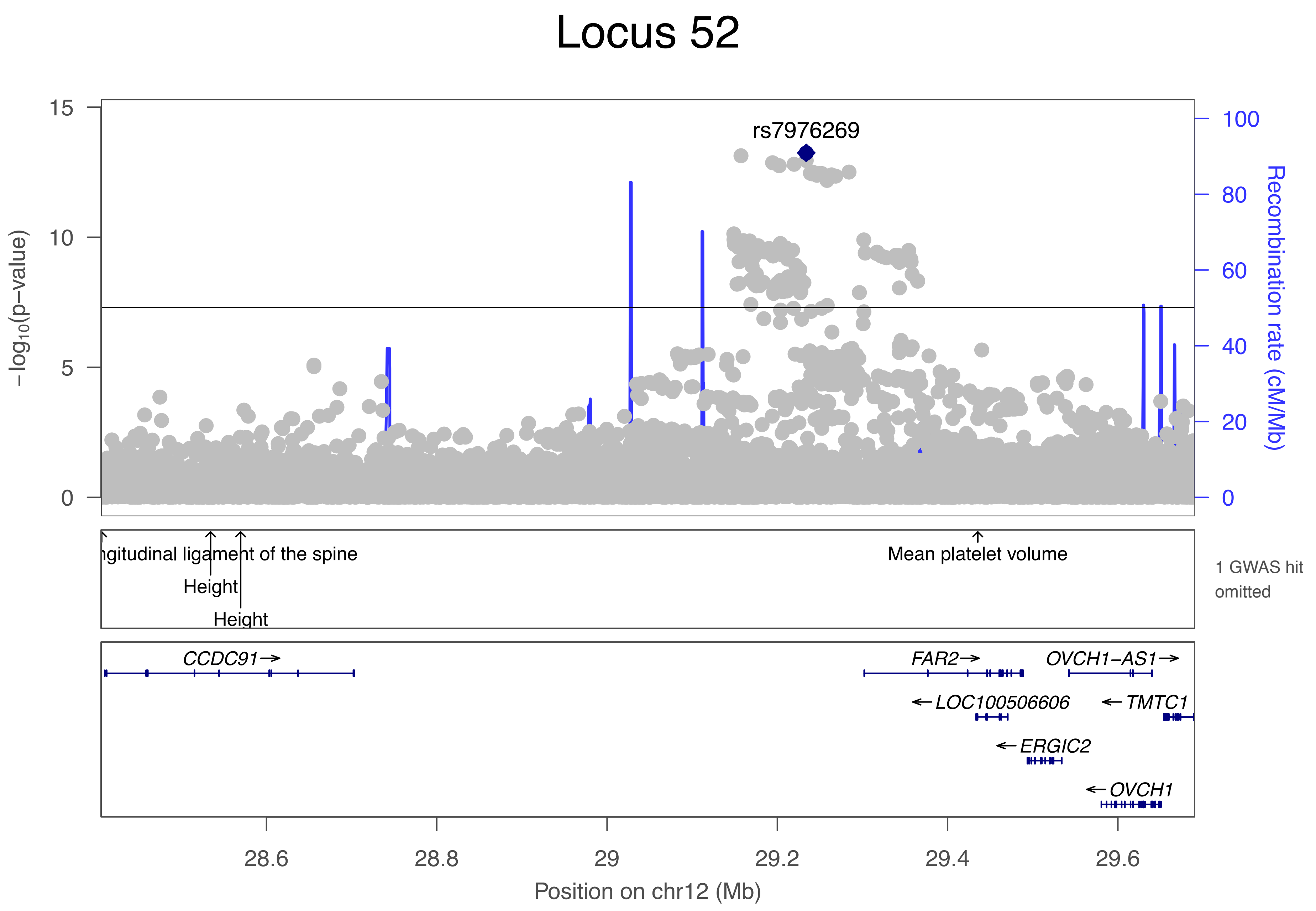

Locus 52
rs7976269
15
10
5
0
−log10(p−value)
100
80
60
40
20
0
Recombination rate (cM/Mb)
ngitudinal ligament of the spine
Height
Height
Mean platelet volume
1 GWAS hit omitted
CCDC91→
FAR2→
OVCH1−AS1→
←LOC100506606
←TMTC1
←ERGIC2
←OVCH1
28.6
28.8
29
29.2
29.4
29.6
Position on chr12 (Mb)

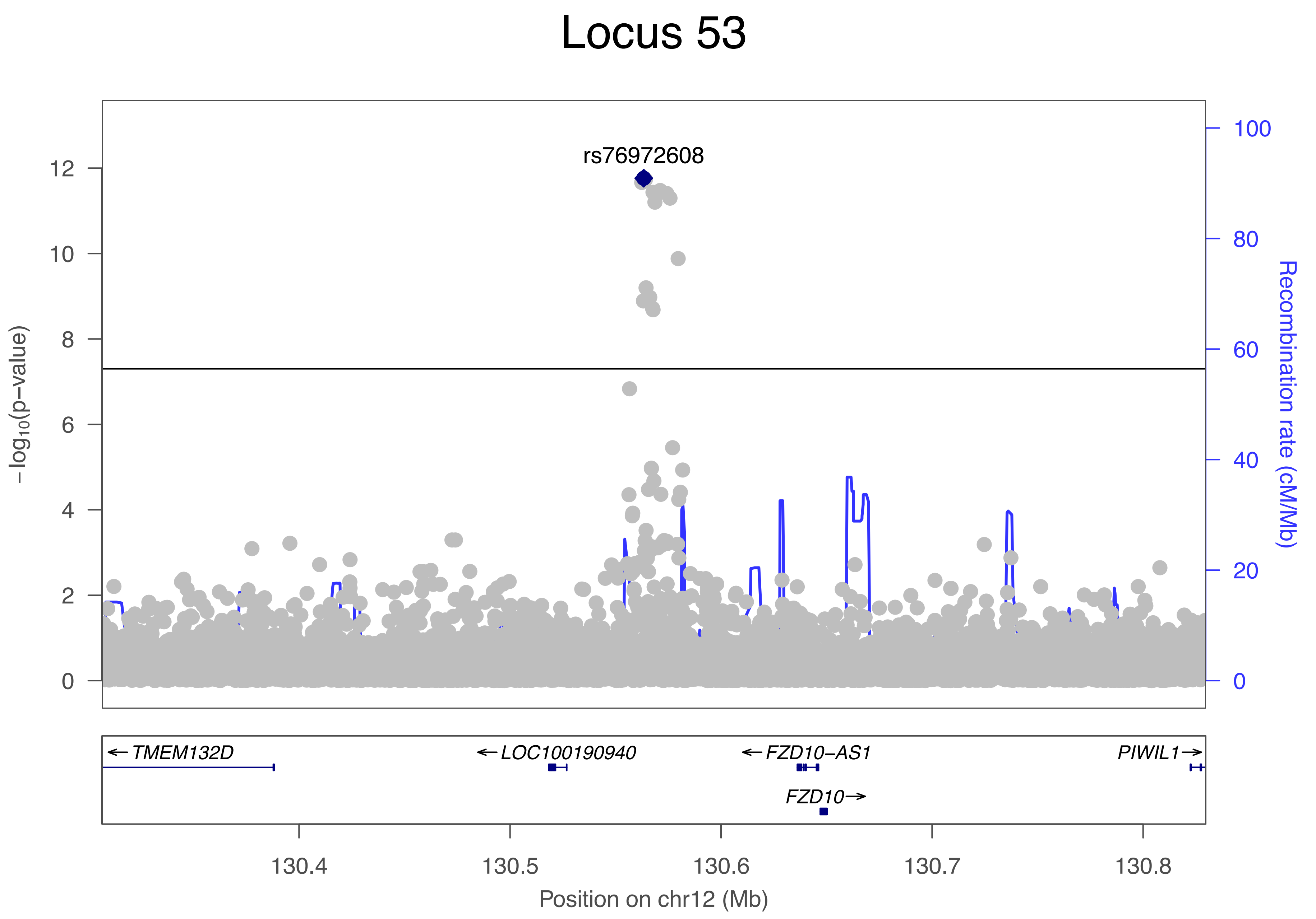

Locus 53
rs76972608
−log10(p−value)
Recombination rate (cM/Mb)
12
10
8
6
4
2
0
100
80
60
40
20
0
← TMEM132D
← LOC100190940
← FZD10−AS1
PIWIL1 →
FZD10 →
130.4
130.5
130.6
130.7
130.8
Position on chr12 (Mb)

Locus 54

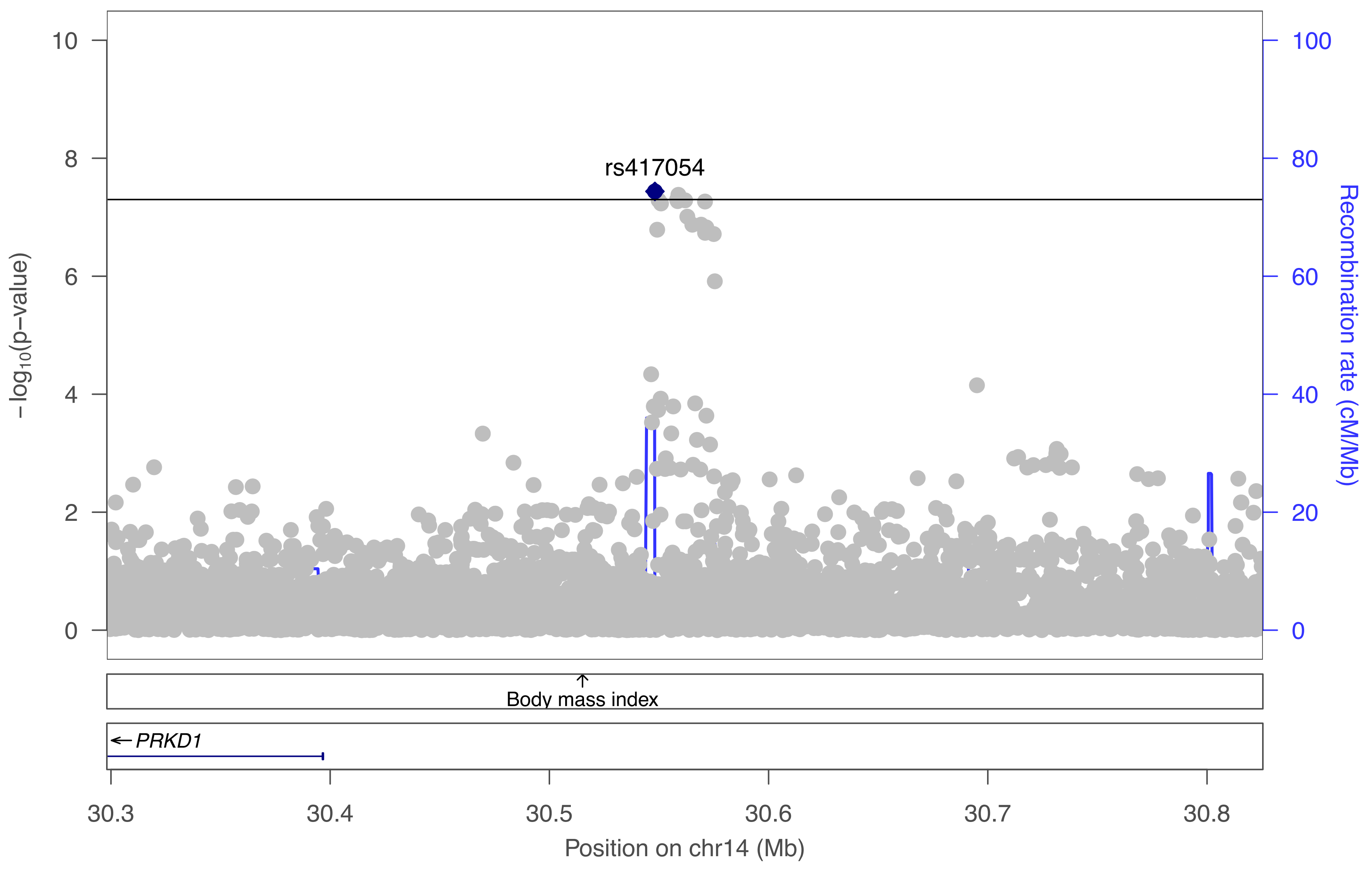

rs417054
−log10(p−value)
Recombination rate (cM/Mb)
0
2
4
6
8
10
0
20
40
60
80
100
Body mass index
PRKD1
30.3
30.4
30.5
30.6
30.7
30.8
Position on chr14 (Mb)

Locus 55

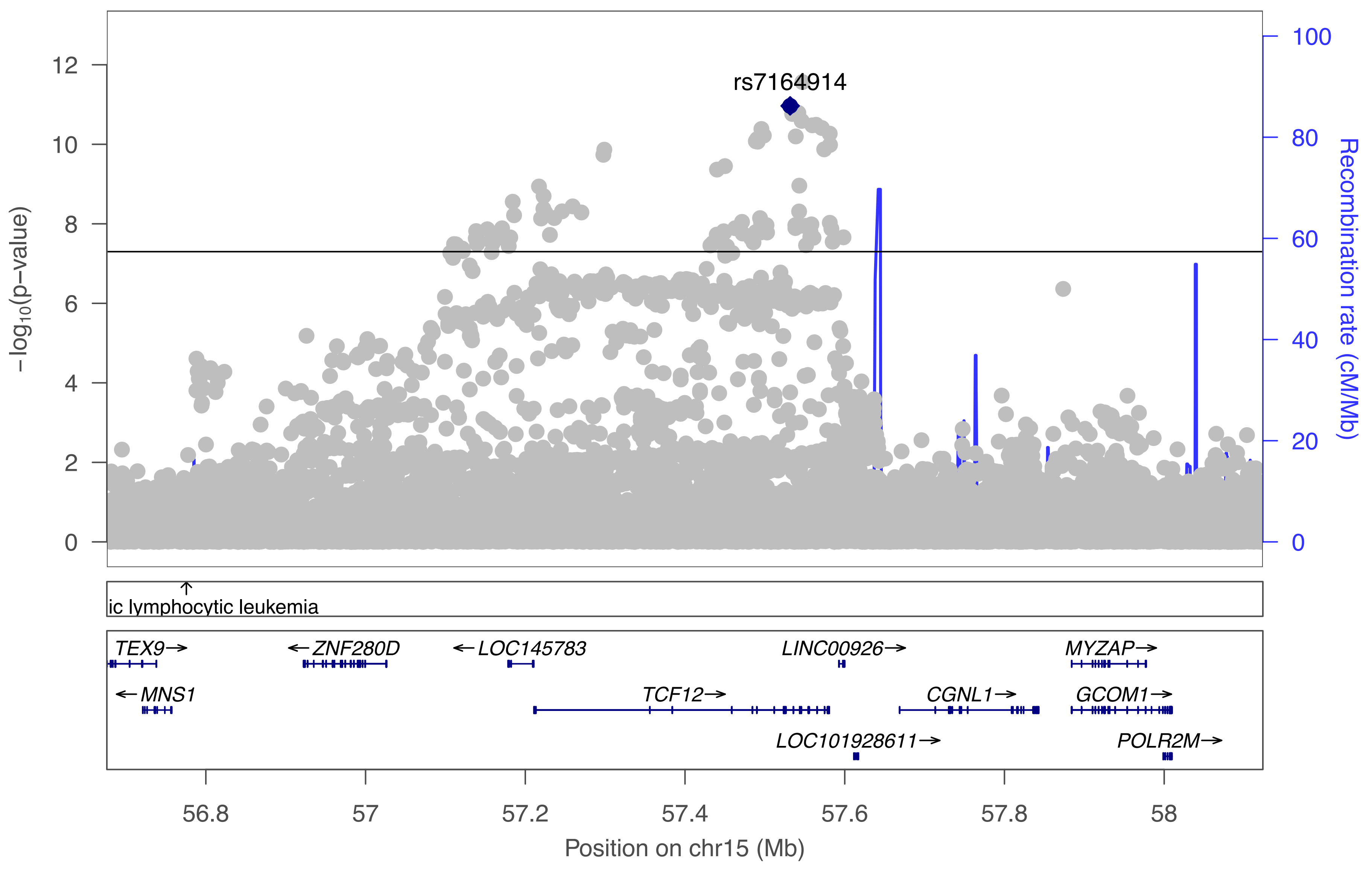

rs7164914
12
10
8
6
4
2
0
−log10(p−value)
100
80
60
40
20
0
Recombination rate (cM/Mb)
ic lymphocytic leukemia
TEX9→
←ZNF280D
←LOC145783
LINC00926→
MYZAP→
←MNS1
TCF12→
CGNL1→
GCOM1→
LOC101928611→
POLR2M→
56.8
57
57.2
57.4
57.6
57.8
58
Position on chr15 (Mb)

Locus 56

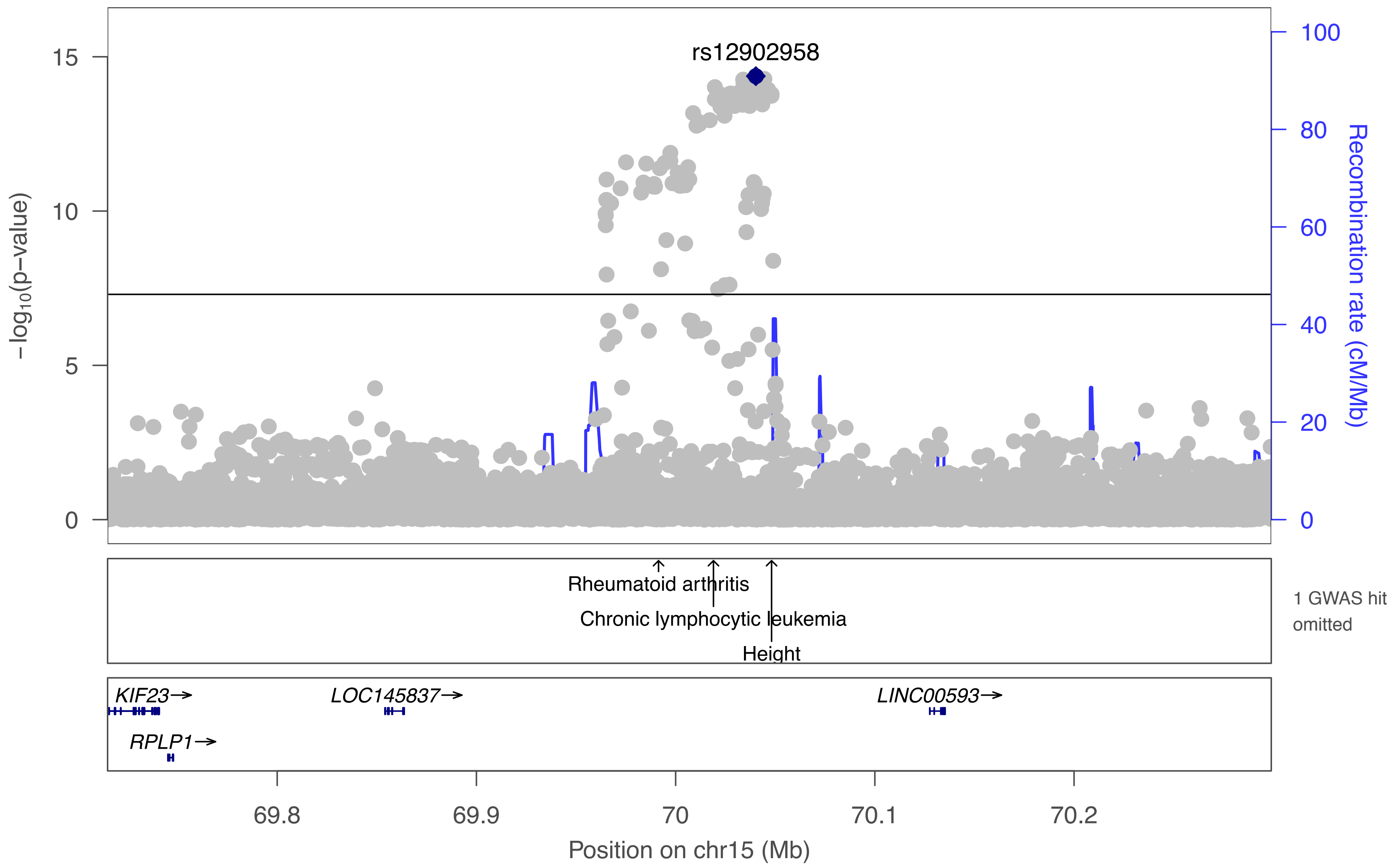
rs12902958
−log10(p−value)
15
10
5
0
Recombination rate (cM/Mb)
100
80
60
40
20
0
Rheumatoid arthritis
Chronic lymphocytic leukemia
Height
1 GWAS hit omitted
KIF23→
LOC145837→
LINC00593→
RPLP1→
69.8
69.9
70
70.1
70.2
Position on chr15 (Mb)

Locus 57

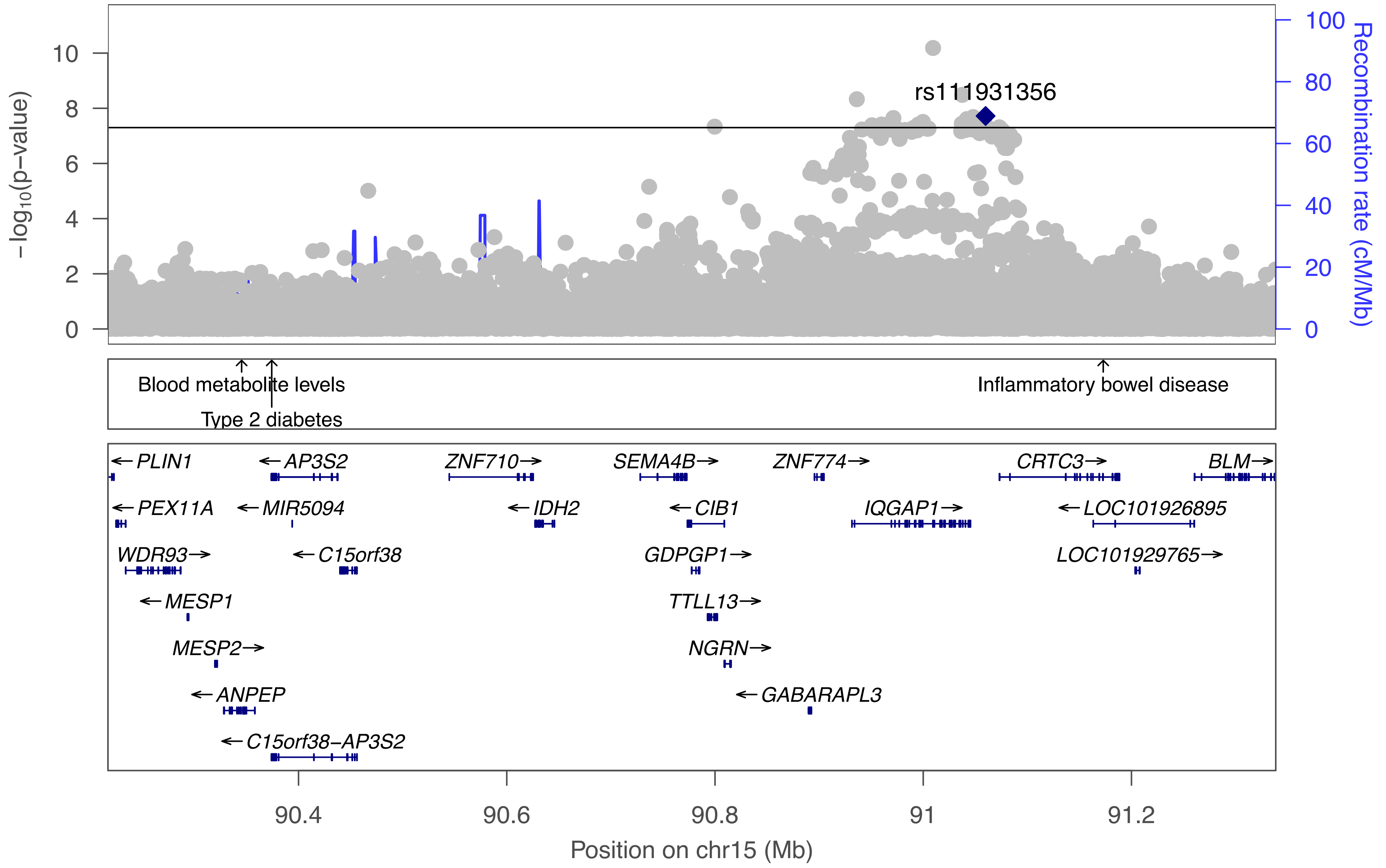

rs111931356
−log10(p−value)
Recombination rate (cM/Mb)
0
2
4
6
8
10
20
40
60
80
100
Blood metabolite levels
Type 2 diabetes
Inflammatory bowel disease
PLIN1
AP3S2
ZNF710
SEMA4B
ZNF774
CRTC3
BLM
PEX11A
MIR5094
IDH2
CIB1
IQGAP1
LOC101926895
WDR93
C15orf38
GDPGP1
LOC101929765
MESP1
TTLL13
MESP2
NGRN
ANPEP
GABARAPL3
C15orf38−AP3S2
90.4
90.6
90.8
91
91.2
Position on chr15 (Mb)

Locus 58

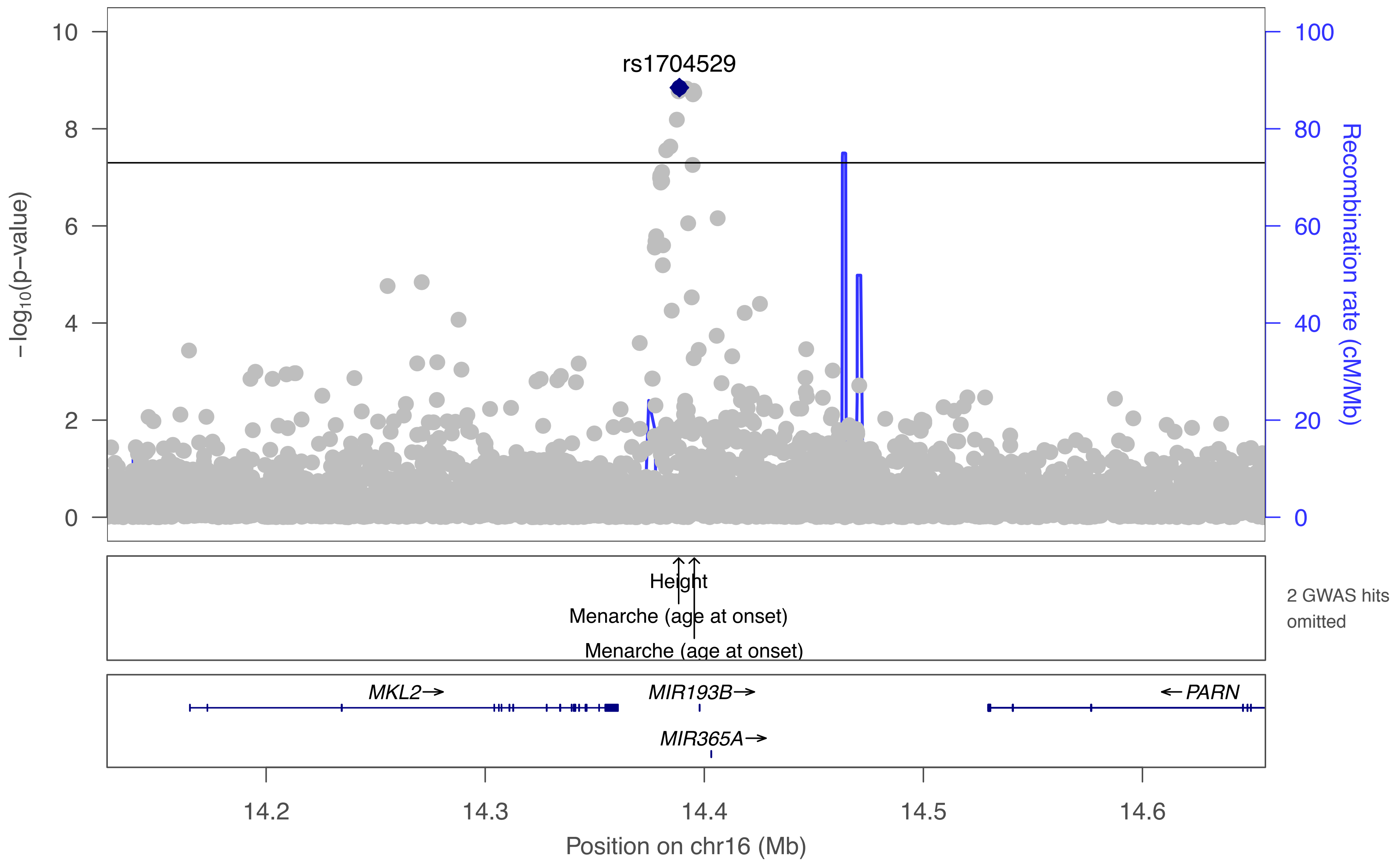
rs1704529
10
8
6
4
2
0
−log10(p−value)
100
80
60
40
20
0
Recombination rate (cM/Mb)
Height
Menarche (age at onset)
Menarche (age at onset)
2 GWAS hits omitted
MKL2→
MIR193B→
←PARN
MIR365A→
14.2
14.3
14.4
14.5
14.6
Position on chr16 (Mb)

Locus 59

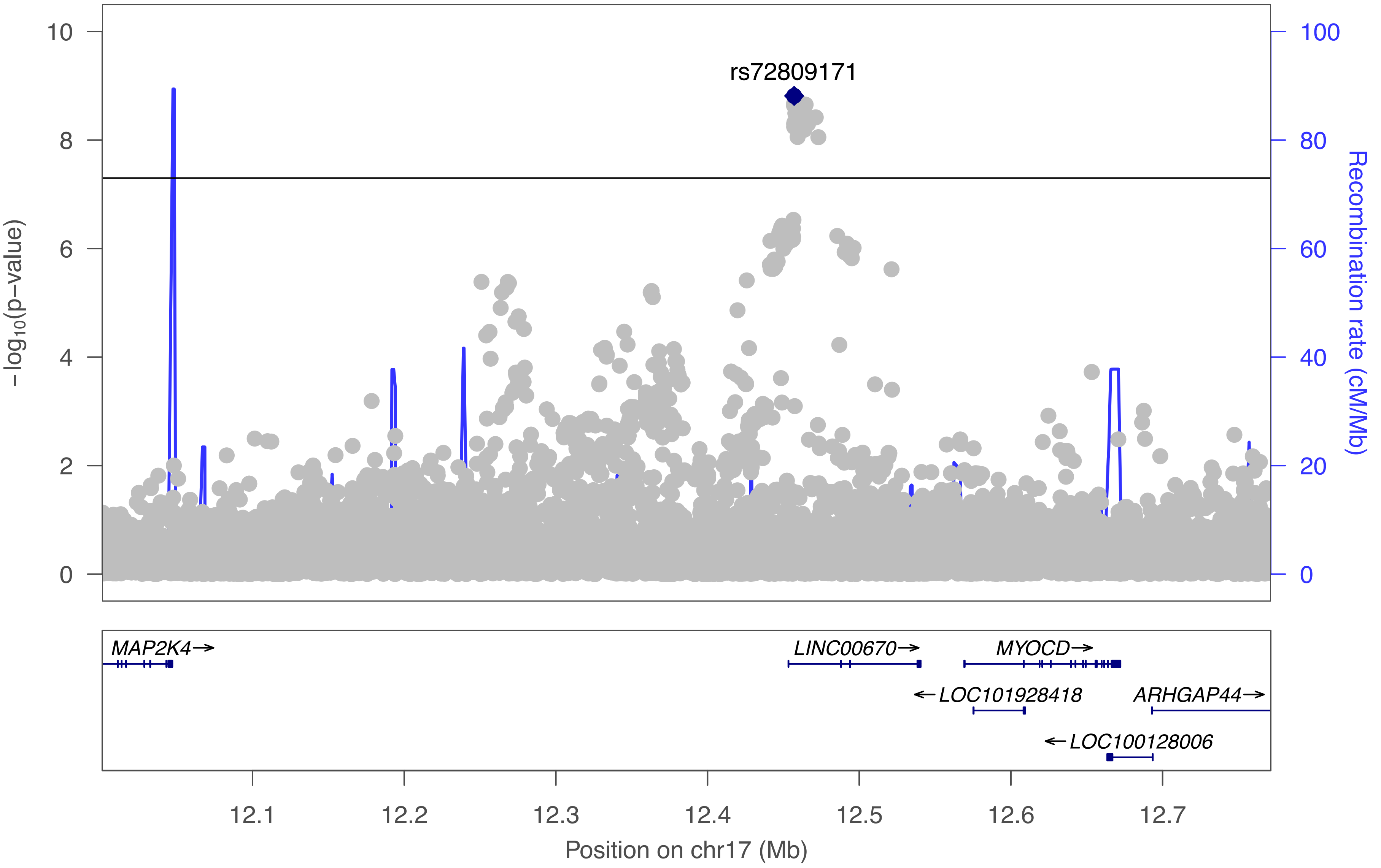

-log10(p-value)
Recombination rate (cM/Mb)
rs72809171
MAP2K4→
LINC00670→
MYOCD→
←LOC101928418
ARHGAP44→
←LOC100128006
Position on chr17 (Mb)

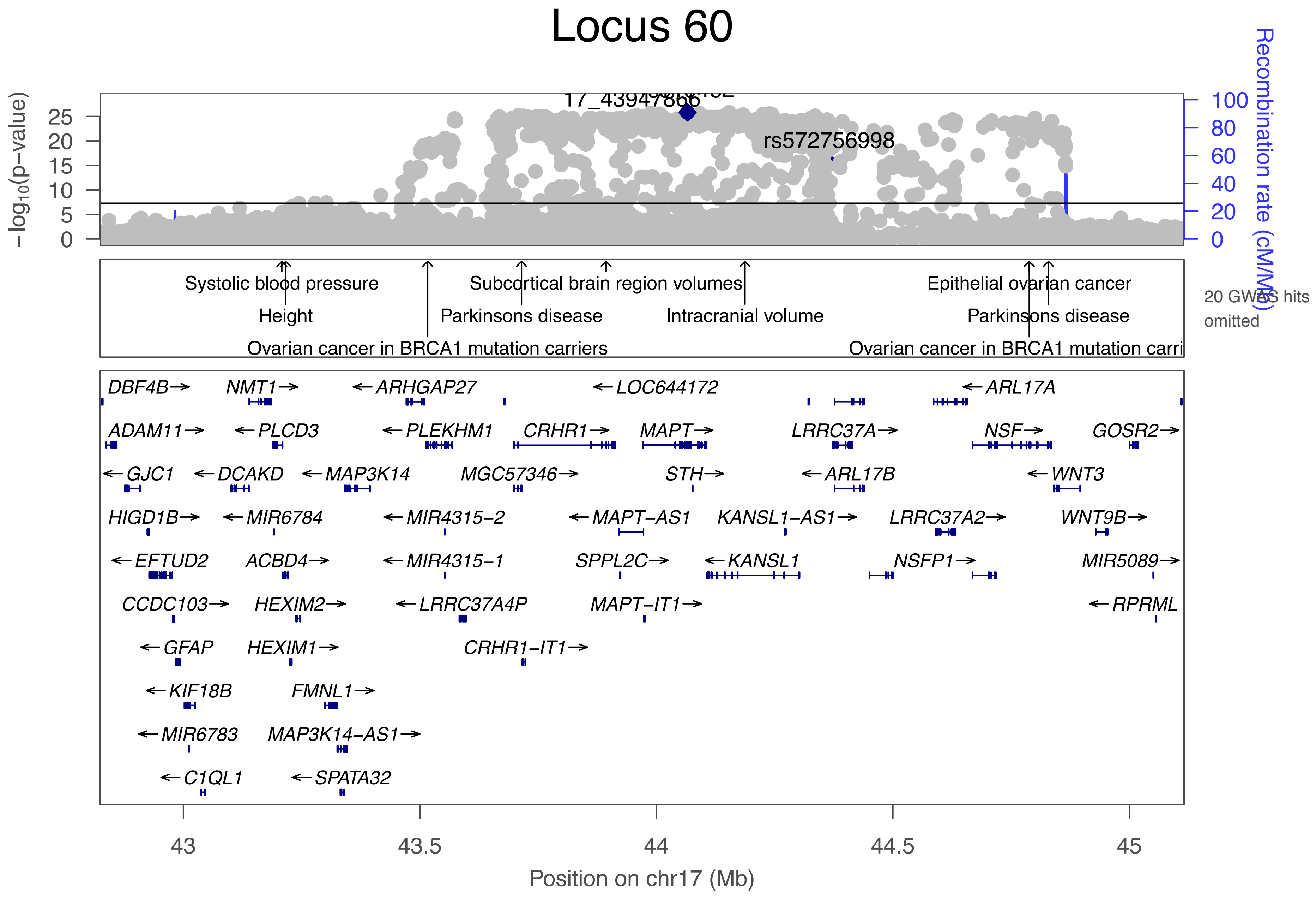

Locus 60
17_43947866
rs572756998
−log10(p−value)
25
20
15
10
5
0
Recombination rate (cM/Mb)
100
80
60
40
20
0
Systolic blood pressure
Height
Ovarian cancer in BRCA1 mutation carriers
Subcortical brain region volumes
Parkinsons disease
Intracranial volume
Epithelial ovarian cancer
Parkinsons disease
Ovarian cancer in BRCA1 mutation carri
20 GWAS hits omitted
DBF4B→
NMT1→
←ARHGAP27
←LOC644172
←ARL17A
ADAM11→
←PLCD3
←PLEKHM1
CRHR1→
MAPT→
LRRC37A→
NSF→
GOSR2→
←GJC1
←DCAKD
←MAP3K14
MGC57346→
STH→
←ARL17B
←WNT3
HIGD1B→
←MIR6784
←MIR4315−2
←MAPT−AS1
KANSL1−AS1→
LRRC37A2→
WNT9B→
←EFTUD2
ACBD4→
←MIR4315−1
SPPL2C→
←KANSL1
NSFP1→
MIR5089→
CCDC103→
HEXIM2→
←LRRC37A4P
MAPT−IT1→
←RPRML
←GFAP
HEXIM1→
CRHR1−IT1→
←KIF18B
FMNL1→
←MIR6783
MAP3K14−AS1→
←C1QL1
←SPATA32
43
43.5
44
44.5
45
Position on chr17 (Mb)

Locus 61

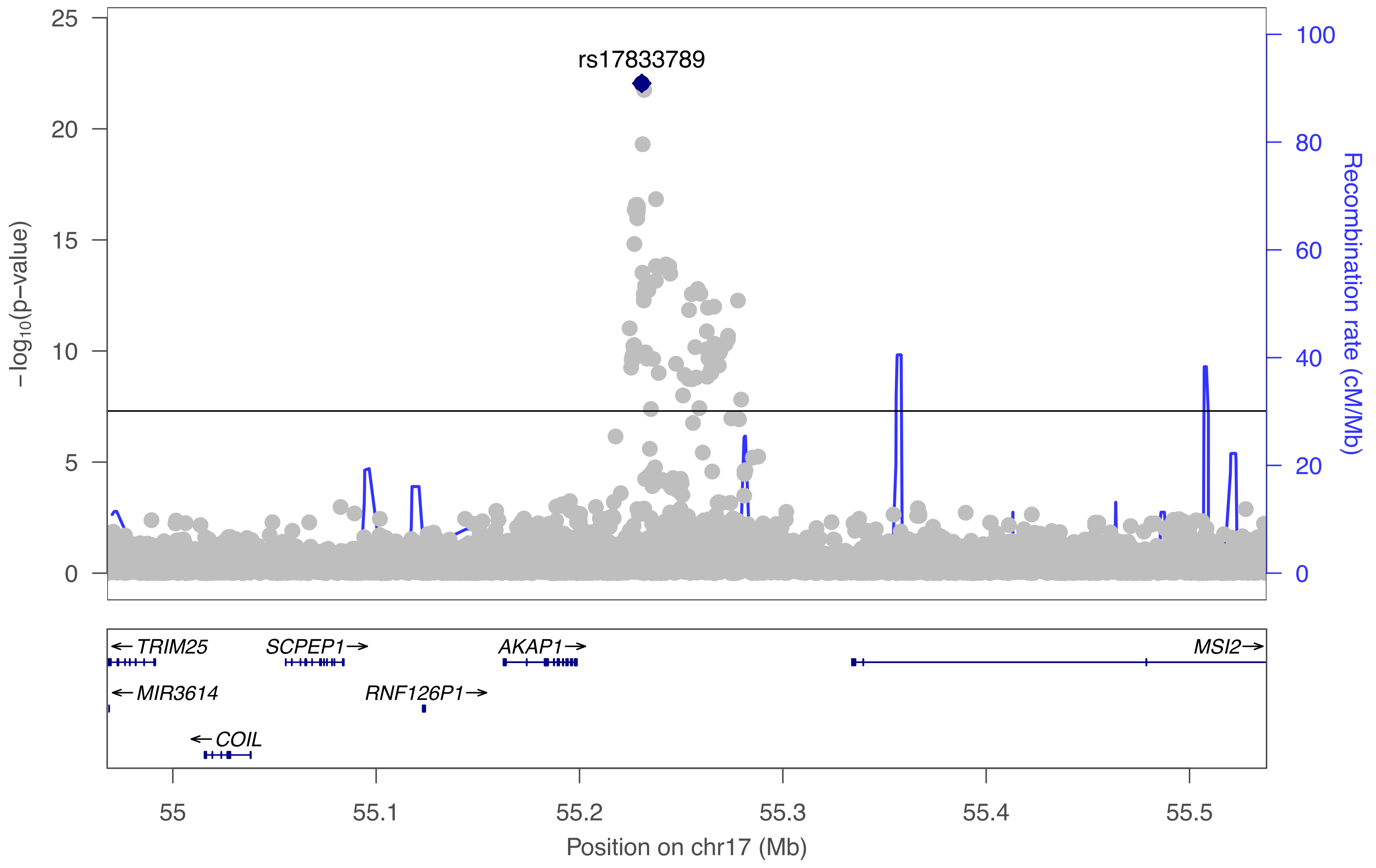

rs17833789
−log10(p−value)
Recombination rate (cM/Mb)
TRIM25
SCPEP1
AKAP1
MSI2
MIR3614
RNF126P1
COIL
Position on chr17 (Mb)

Locus 62

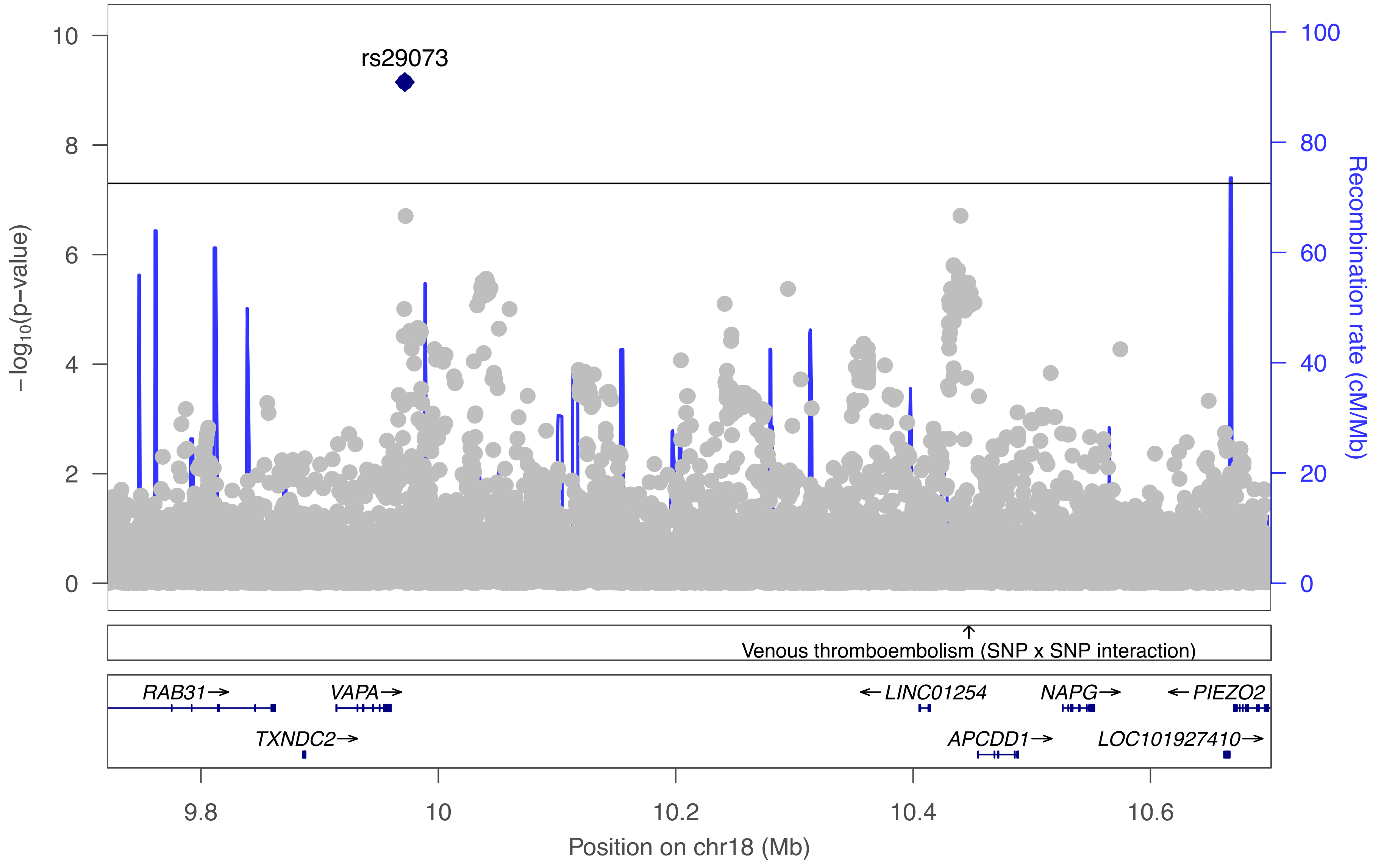

rs29073
−log10(p−value)
Recombination rate (cM/Mb)
10
8
6
4
2
0
100
80
60
40
20
0
Venous thromboembolism (SNP x SNP interaction)
RAB31→
VAPA→
←LINC01254
NAPG→
←PIEZO2
TXNDC2→
APCDD1→
LOC101927410→
9.8
10
10.2
10.4
10.6
Position on chr18 (Mb)

Locus 63

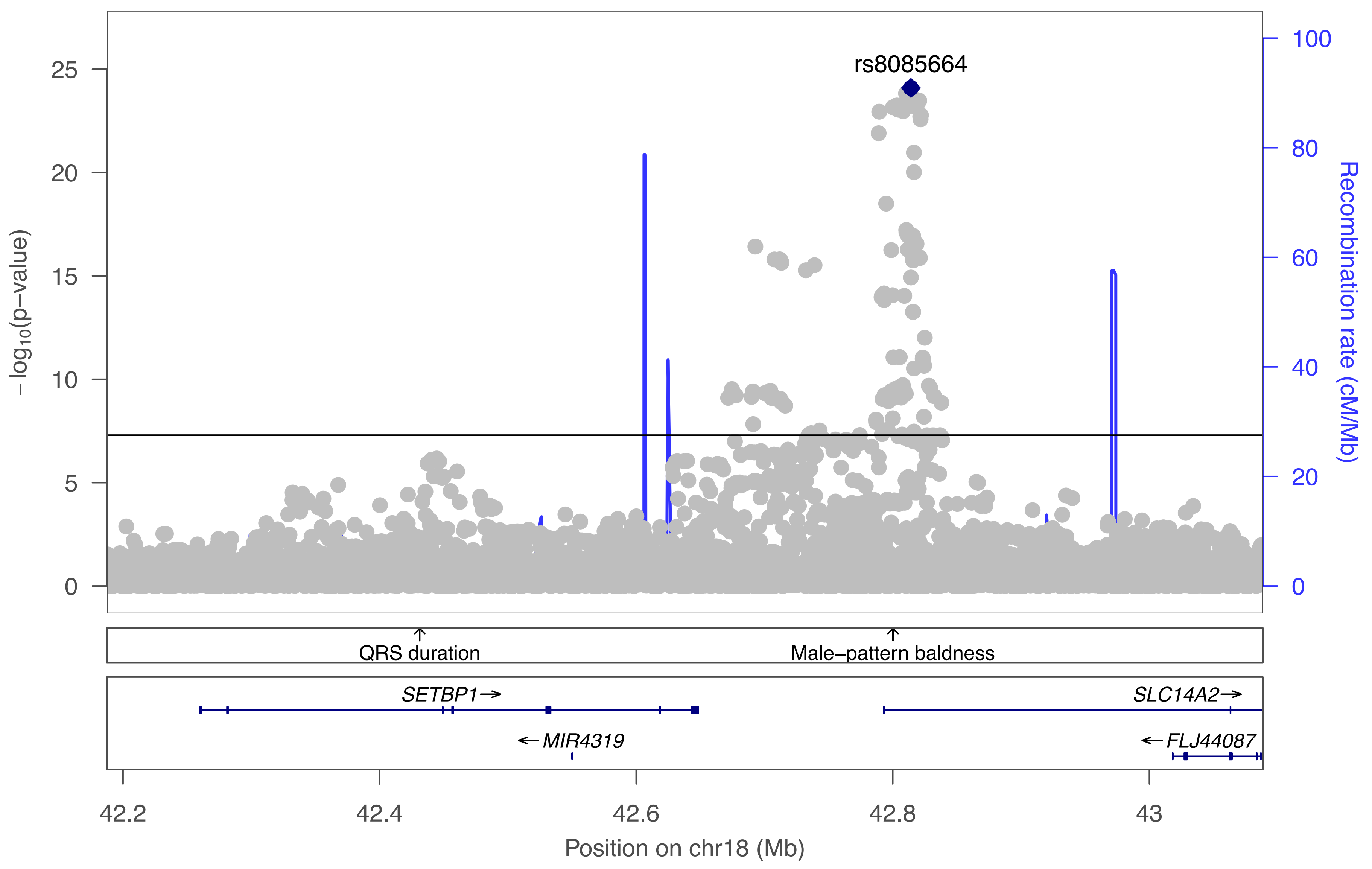

rs8085664
−log10(p−value)
Recombination rate (cM/Mb)
0
5
10
15
20
25
0
20
40
60
80
100
QRS duration
Male−pattern baldness
SETBP1→
←MIR4319
SLC14A2→
←FLJ44087
42.2
42.4
42.6
42.8
43
Position on chr18 (Mb)

Locus 64

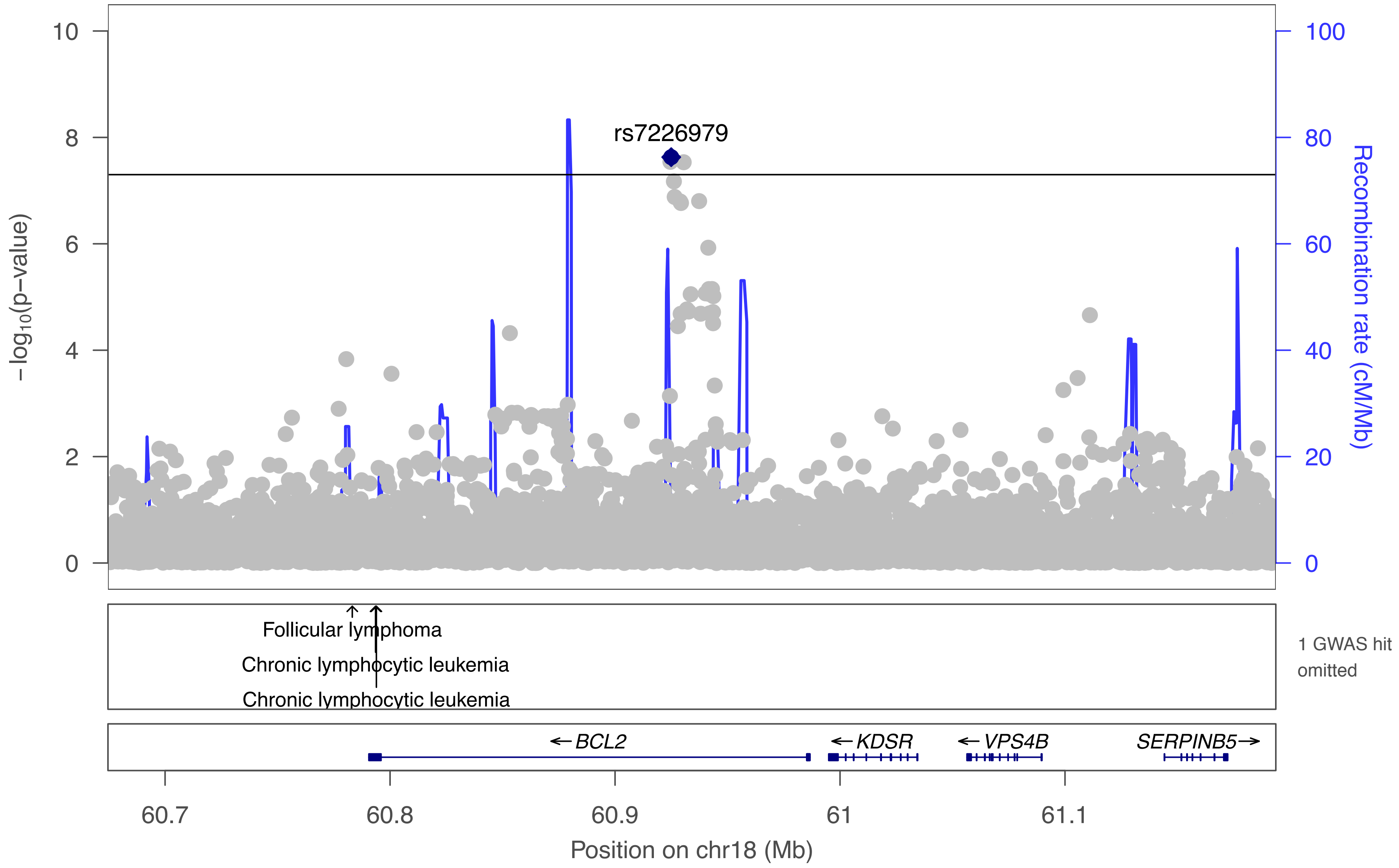

rs7226979
−log10(p−value)
Recombination rate (cM/Mb)
0
2
4
6
8
10
0
20
40
60
80
100
Follicular lymphoma
Chronic lymphocytic leukemia
Chronic lymphocytic leukemia
1 GWAS hit omitted
BCL2
KDSR
VPS4B
SERPINB5
60.7
60.8
60.9
61
61.1
Position on chr18 (Mb)

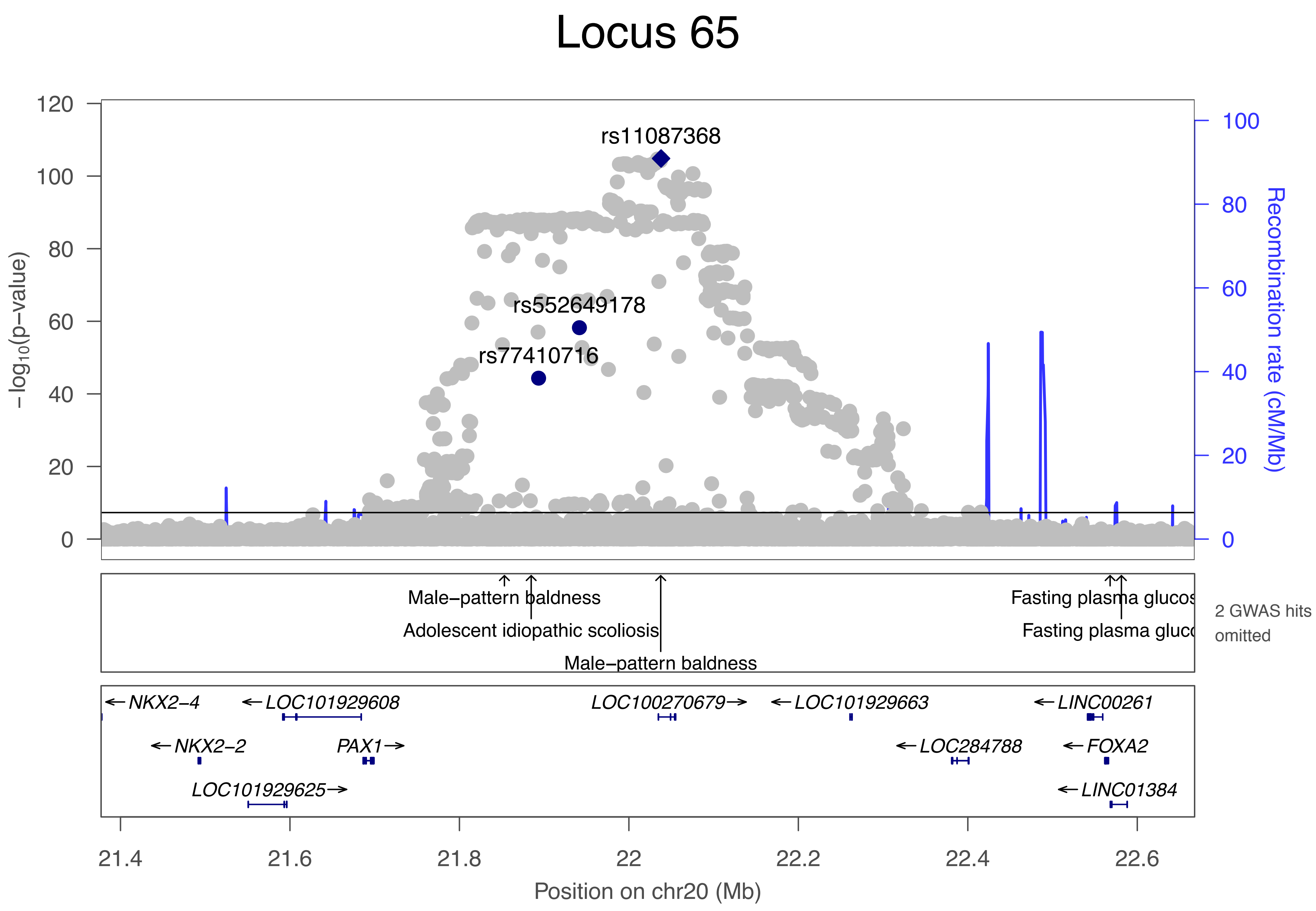
Locus 65
rs11087368
rs552649178
rs77410716
−log10(p−value)
Recombination rate (cM/Mb)
Male−pattern baldness
Adolescent idiopathic scoliosis
Male−pattern baldness
Fasting plasma glucos
Fasting plasma gluco
2 GWAS hits omitted
NKX2−4
LOC101929608
LOC100270679
LOC101929663
LINC00261
NKX2−2
PAX1
LOC284788
FOXA2
LOC101929625
LINC01384
Position on chr20 (Mb)

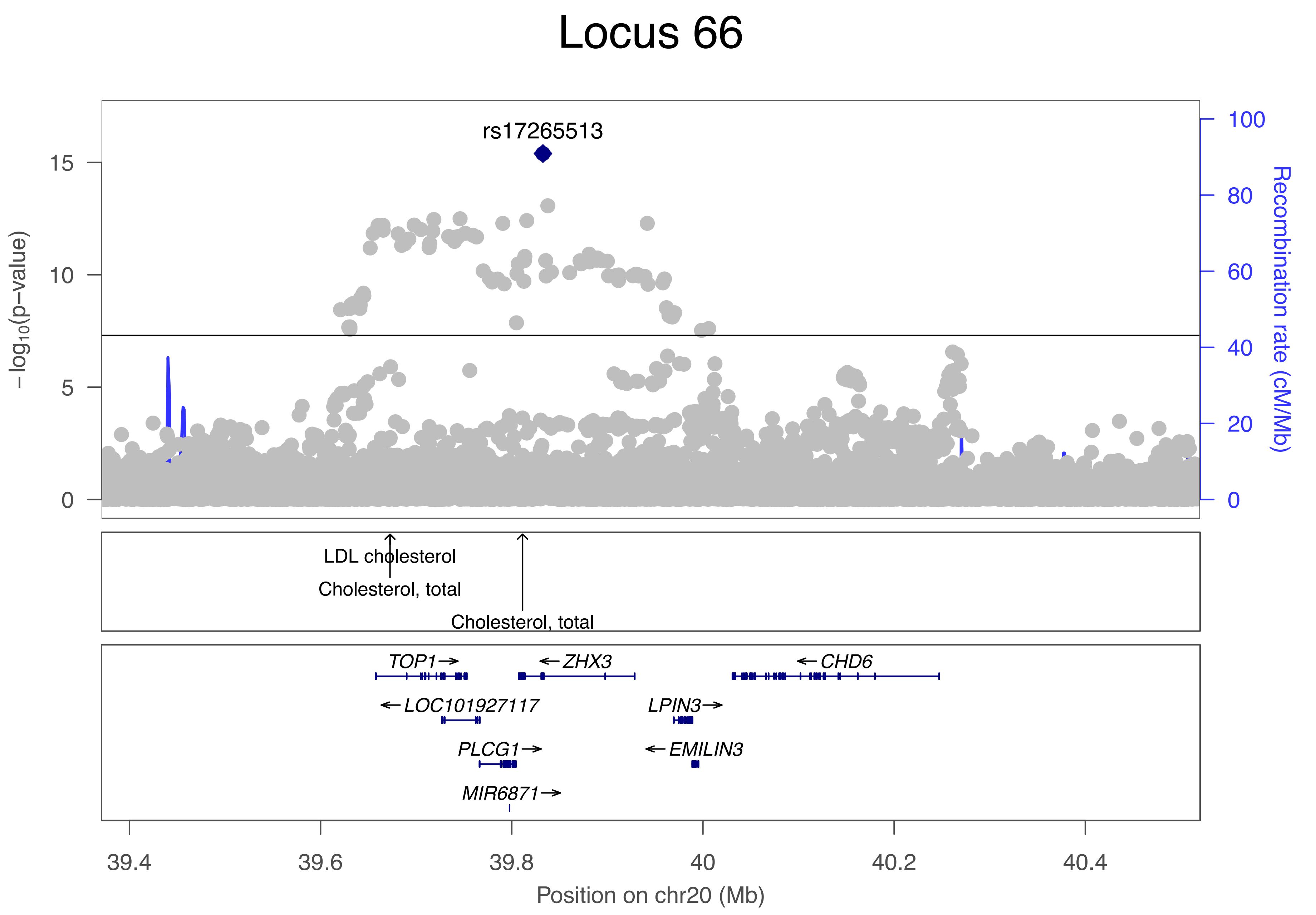

Locus 66
rs17265513
−log10(p−value)
Recombination rate (cM/Mb)
0
5
10
15
0
20
40
60
80
100
LDL cholesterol
Cholesterol, total
Cholesterol, total
TOP1→
←ZHX3
←CHD6
←LOC101927117
LPIN3→
PLCG1→
←EMILIN3
MIR6871→
39.4
39.6
39.8
40
40.2
40.4
Position on chr20 (Mb)

Locus 67

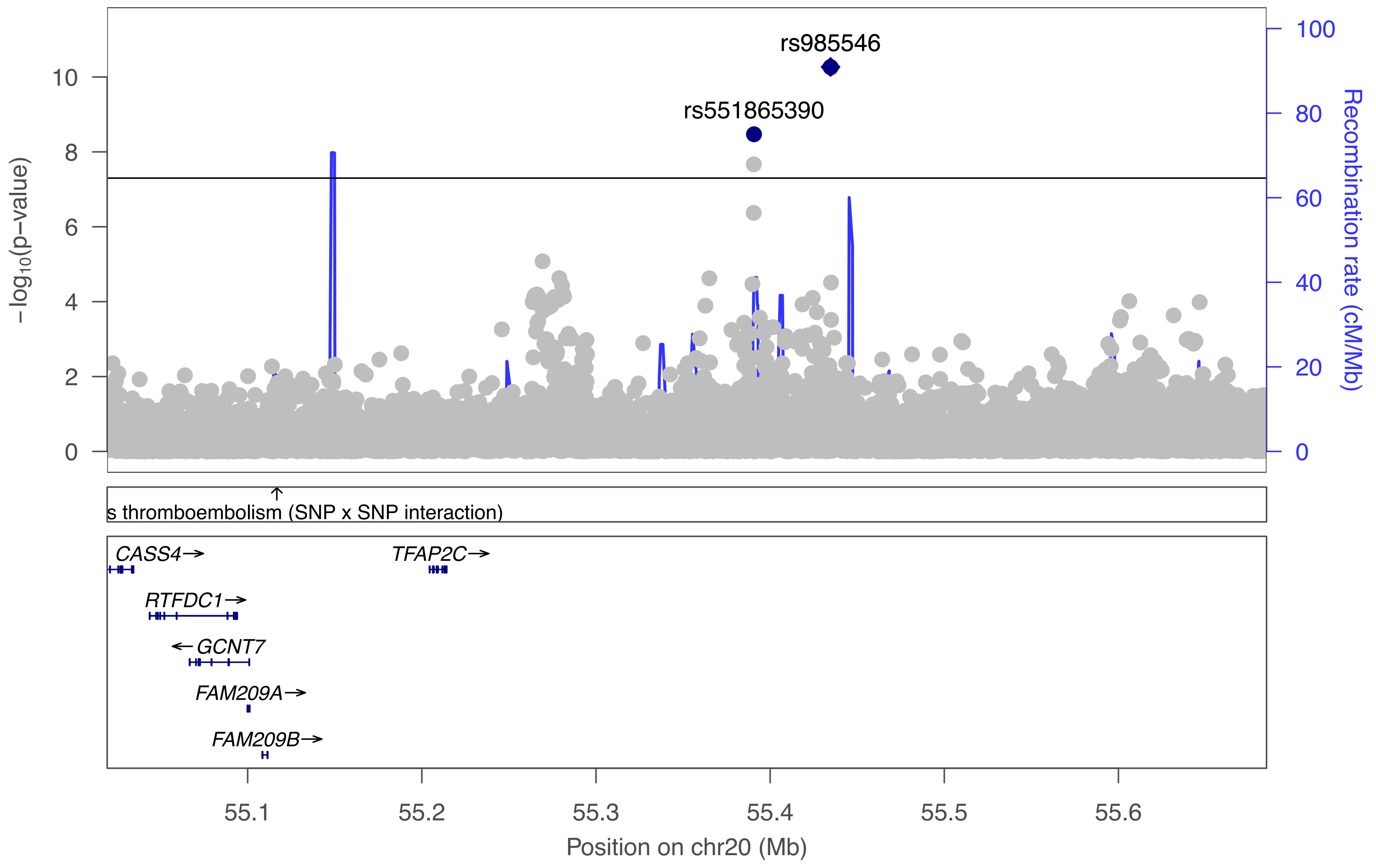
rs985546
rs551865390
−log10(p−value)
Recombination rate (cM/Mb)
0
2
4
6
8
10
20
40
60
80
100
s thromboembolism (SNP x SNP interaction)
CASS4→
TFAP2C→
RTFDC1→
←GCNT7
FAM209A→
FAM209B→
55.1
55.2
55.3
55.4
55.5
55.6
Position on chr20 (Mb)

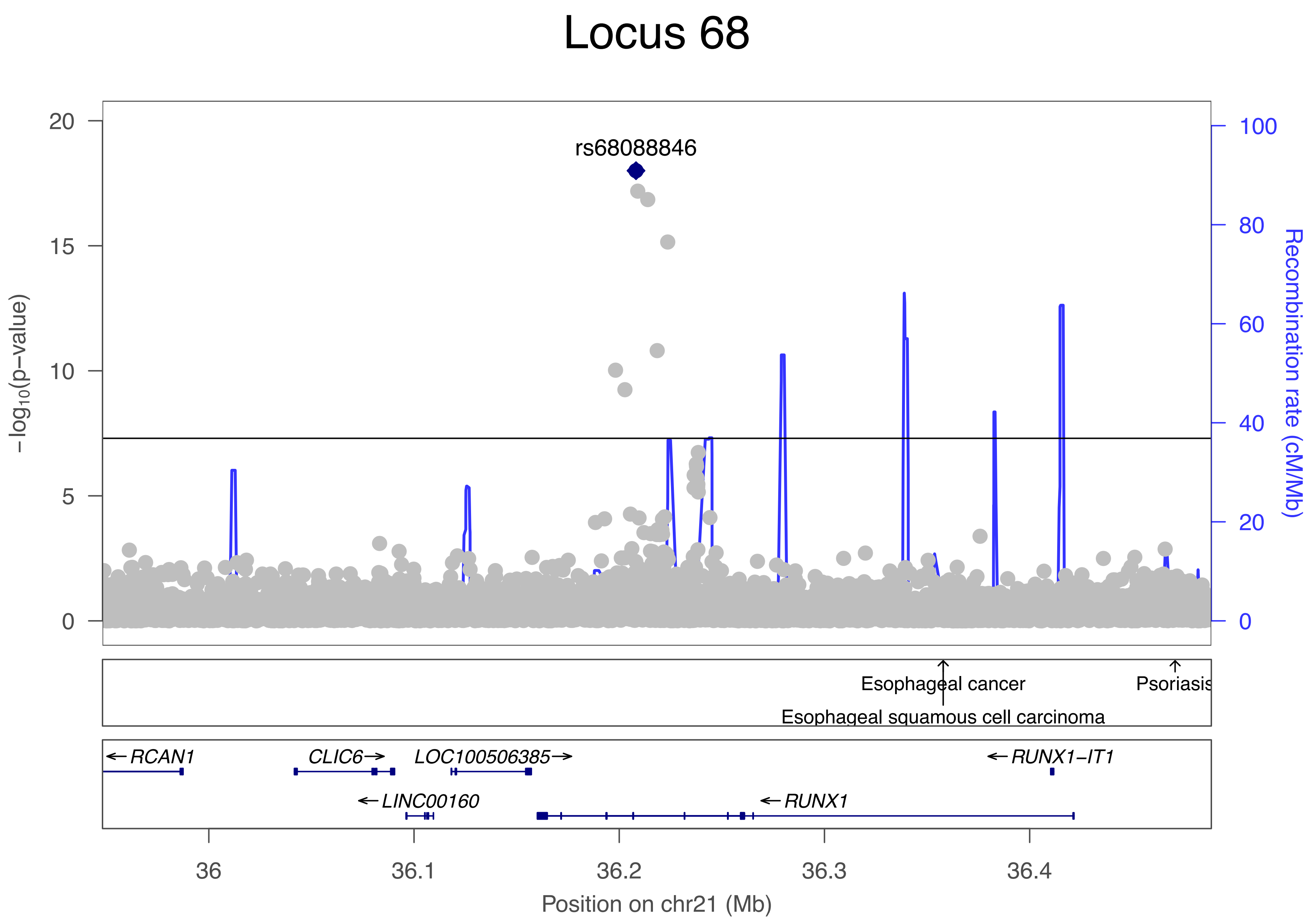

Locus 68
rs68088846
−log10(p−value)
Recombination rate (cM/Mb)
20
15
10
5
0
100
80
60
40
20
0
Esophageal cancer
Esophageal squamous cell carcinoma
Psoriasis
← RCAN1
CLIC6 →
LOC100506385 →
← RUNX1−IT1
← LINC00160
← RUNX1
36
36.1
36.2
36.3
36.4
Position on chr21 (Mb)

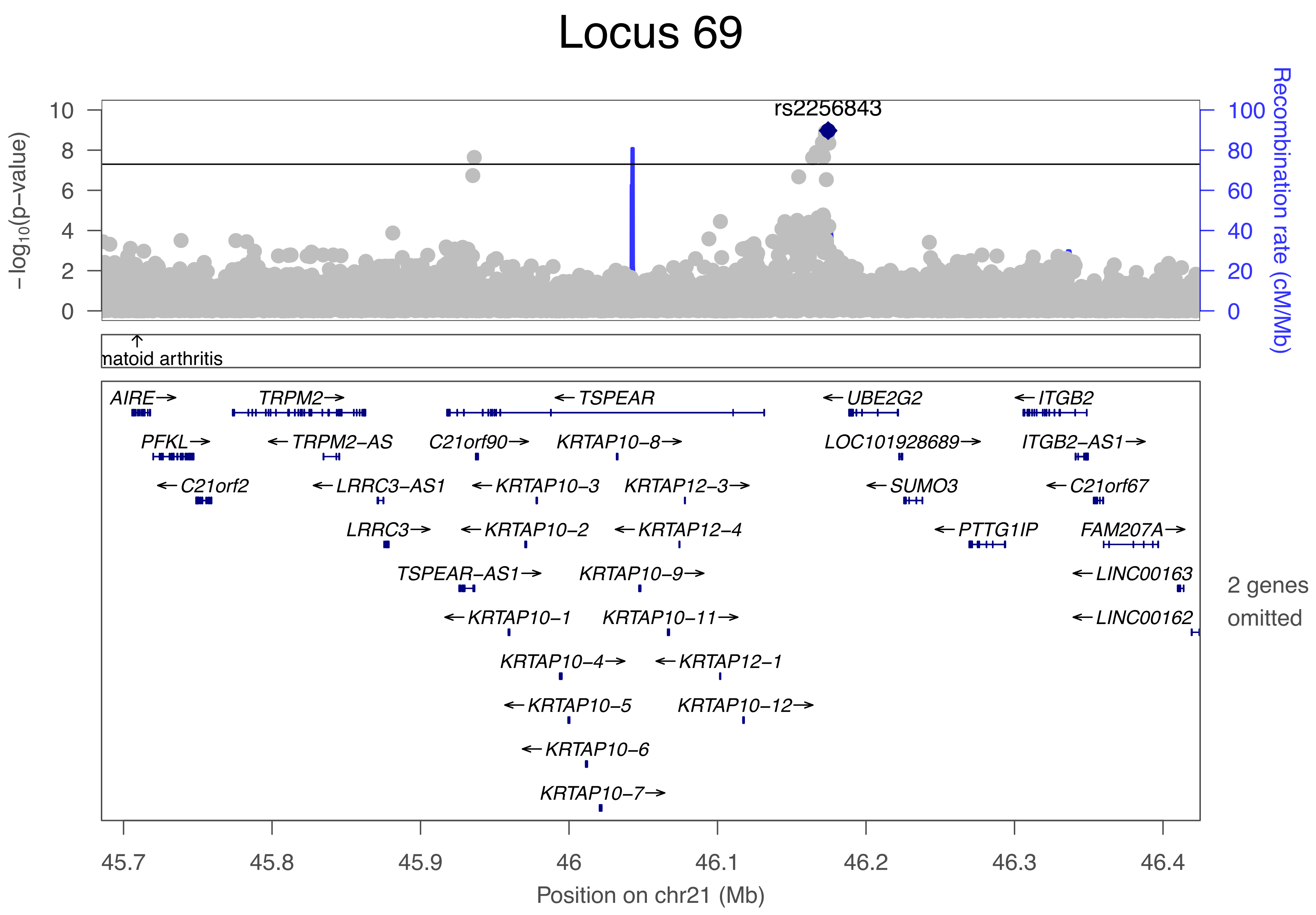

Locus 69
rs2256843
−log10(p−value)
Recombination rate (cM/Mb)
10
8
6
4
2
0
100
80
60
40
20
0
matoid arthritis
AIRE→
TRPM2→
←TSPEAR
←UBE2G2
←ITGB2
PFKL→
←TRPM2−AS
C21orf90→
KRTAP10−8→
LOC101928689→
ITGB2−AS1→
←C21orf2
←LRRC3−AS1
←KRTAP10−3
KRTAP12−3→
←SUMO3
←C21orf67
LRRC3→
←KRTAP10−2
←KRTAP12−4
←PTTG1IP
FAM207A→
TSPEAR−AS1→
KRTAP10−9→
←LINC00163
←KRTAP10−1
KRTAP10−11→
←LINC00162
KRTAP10−4→
←KRTAP12−1
←KRTAP10−5
KRTAP10−12→
←KRTAP10−6
KRTAP10−7→
2 genes omitted
45.7
45.8
45.9
46
46.1
46.2
46.3
46.4
Position on chr21 (Mb)

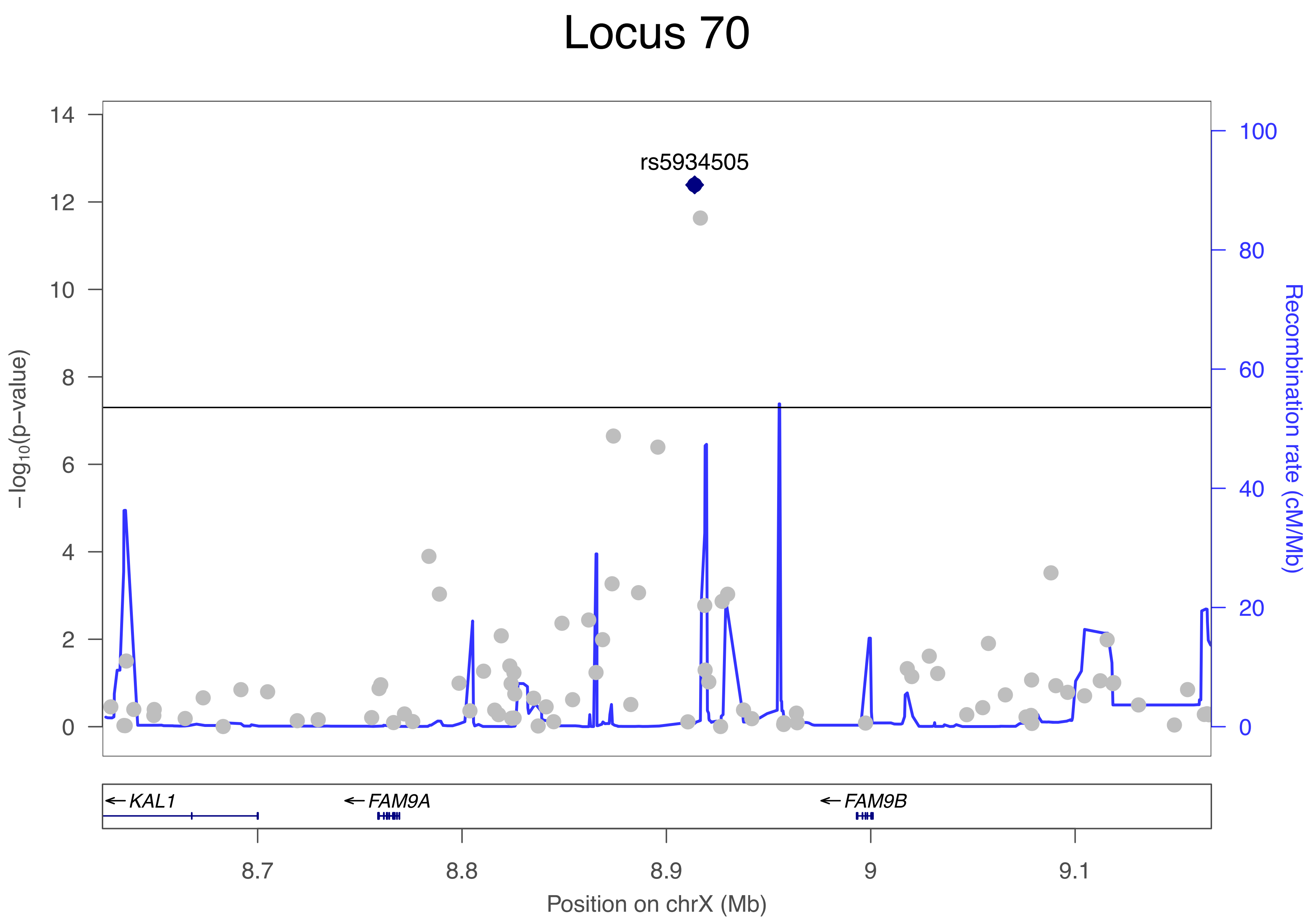
Locus 70
rs5934505
−log10(p−value)
Recombination rate (cM/Mb)
14
12
10
8
6
4
2
0
100
80
60
40
20
0
KAL1
FAM9A
FAM9B
8.7
8.8
8.9
9
9.1
Position on chrX (Mb)

Locus 71

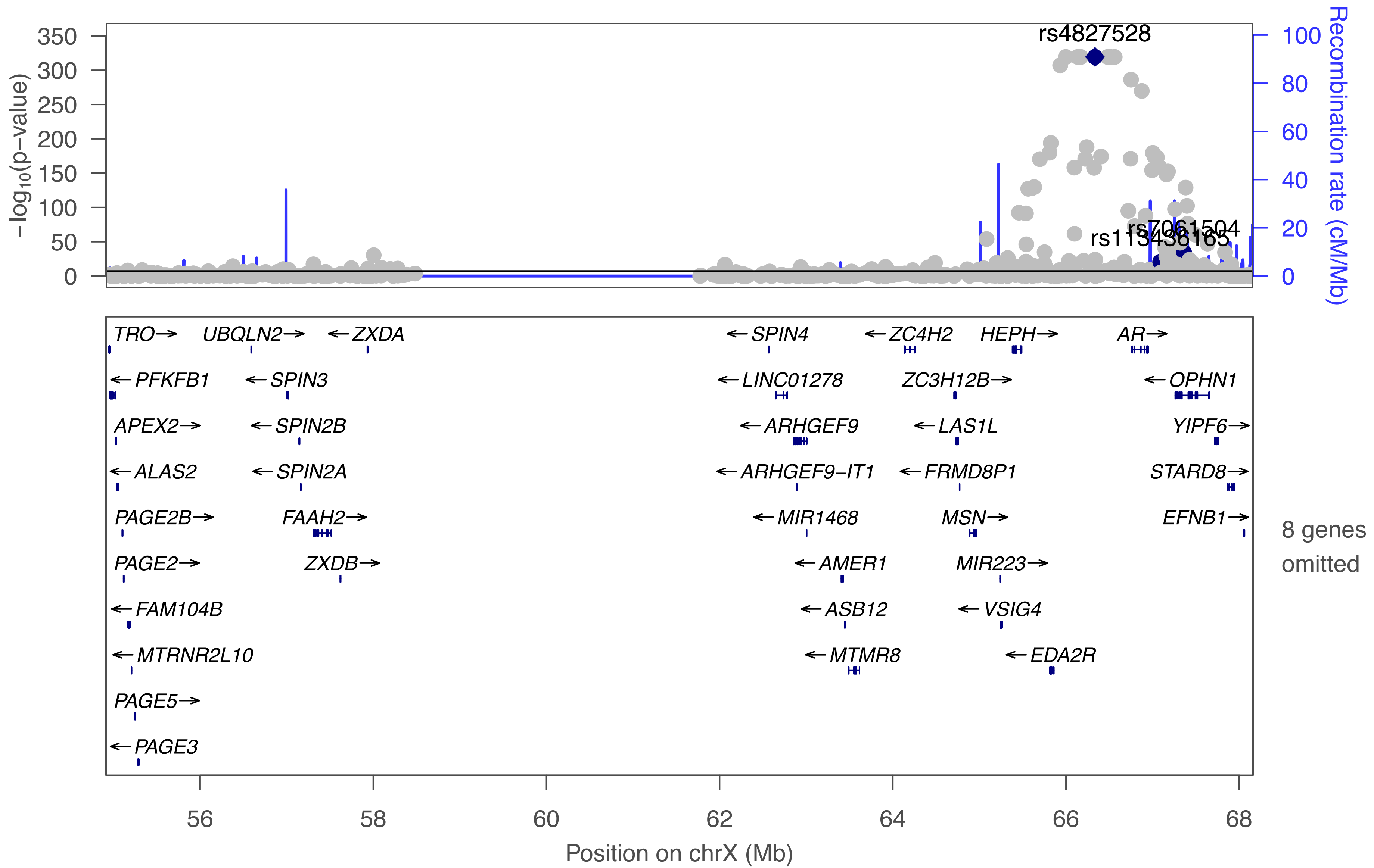

rs4827528
rs7061504
rs113436165
−log10(p−value)
350
300
250
200
150
100
50
0
Recombination rate (cM/Mb)
100
80
60
40
20
0
TRO→
UBQLN2→
←ZXDA
←SPIN4
←ZC4H2
HEPH→
AR→
←PFKFB1
←SPIN3
←LINC01278
ZC3H12B→
←OPHN1
APEX2→
←SPIN2B
←ARHGEF9
←LAS1L
YIPF6→
←ALAS2
←SPIN2A
←ARHGEF9−IT1
←FRMD8P1
STARD8→
PAGE2B→
FAAH2→
←MIR1468
MSN→
EFNB1→
PAGE2→
ZXDB→
←AMER1
MIR223→
←FAM104B
←ASB12
←VSIG4
←MTRNR2L10
←MTMR8
←EDA2R
PAGE5→
←PAGE3
8 genes omitted
56
58
60
62
64
66
68
Position on chrX (Mb)
