# Supplementary material for: GWAS for male-pattern baldness identifies 71 susceptibility loci explaining 38% of the risk
Source: Nat Commun. 2017 Nov 17;8:1584. doi: 10.1038/s41467-017-01490-8 (PMC5691155; doi:10.1038/s41467-017-01490-8)
Supplement: Supplementary file 3 — Description of Additional Supplementary Files [file 41467_2017_1490_MOESM3_ESM.pdf]

File Name: Supplementary Data 1
Description: General Cohort information: The table reports for each cohort the numebr of cases and controls, mean age, scale of baldness assessment and case control definition, genotyping and imputation information.

File Name: Supplementary Data 2
Description: Summary statistics for all significant replicated SNPs: The table reports for each significant replicated SNP the locus to which it belongs, alternate and rsid names, the genomic position, coded and non-coded allele, p-value, beta and standard error at discovery, p-value, beta and standard error at replication.

File Name: Supplementary Data 3
Description: Summary statistics for all distinct SNPs. The table reports for each distinct SNP the locus to which it belongs, alternate and rsid names, the genomic position, coded and non-coded allele, p-value, beta and standard error at discovery, p-value, beta and standard error at replication and MAF in the discovery cohort.

File Name: Supplementary Data 4
Description: Haploreg enrichment analysis The table reports the results from the histon mark enrichment analysis conducted using Haploreg v4.1. The first column describes the cell type, the second the observed number of SNPs in the specific cell type, the expected number of SNPs over all SNPs or GWAS snps and p-values for both categories.

File Name: Supplementary Data 5
Description: DEPICT Tissue enrichment analysis. The table reports the results of the tissue enrichment analysis conducted with DEPICT. No SNPs were significant at FDR 0.05.

File Name: Supplementary Data 6
Description: DEPICT Gene-Set enrichment analysis. The table reports the results of the Gene set analysis conducted with DEPICT.

File Name: Supplementary Data 7
Description: Prioritization of genes. The table reports all genes in the identified loci as mapped with DEPICT. Each gene was evaluated according to different criteria, the final column reports for each gene the number of criteria met.

File Name: Supplementary Data 8
Description: Results from the pathway enrichment analysis performed over the whole set of genes present in all identified loci without any prioritization.

File Name: Supplementary Data 9
Description: Selected genes. The table reports the genes selected after the custom prioritization. ENSID refers to Ensemble gene id.

File Name: Supplementary Data 10
Description: List of druggable genes amongst the prioritized genes.

File Name: Supplementary Data 11
Description: List of pathways considered for defining genes associated to androgens for the prioritization.

File Name: Supplementary Data 12
Description: Enrichment pathway analysis results using ConsensusDBPhathway.

File Name: Supplementary Data 13
Description: Enrichment gene-set analysis results using ConsensusDBPhathway.

File Name: Supplementary Data 14
Description: Community analysis based on the adiancency matrix constructed on the co-membership to enriched gene-sets.

File Name: Supplementary Data 15
Description: Enrichment analysis based on the genes in the "Signalling" Community.

File Name: Supplementary Data 16
Description: Enrichment analysis based on the genes in the "Nuclear" Community.

File Name: Supplementary Data 17
Description: Correlation between the association results in each locus and previous traits estimated using the GENOSCORE platform.

File Name: Supplementary Data 18
Description: Results for the LD score regression correlations analysis conducted with LD-Hub

File Name: Supplementary Data 19
Description: Traits significant in polygenic risk score regressions. The first column reports the trait analysed. The second the name of the PGRS, n represents the number of samples and events, the number of observed cases. Beta represents the regression coefficient, the scale is quantitative, logistic or hazard ratio depending on the trait. z represents the z score from the analysis. Analysis model refers to the type of trait analysed and finally q-values have been reported.

File Name: Supplementary Data 20
Description: Full results for the PGRS regressions. The first column reports the trait the suffixes off, fath and moth refers to the trait considered, off if it was the participant to the study,, moth its mother and fath its father. The second the name of the PGRS, n represents the number of samples while events the number of observed cases. Beta represents the regression coefficient, the scale is quantitative , logistic or hazard ratio depending on the trait. exp_beta refers to the exp of beta and is reported only for logistic and cox regressions. z represents the z score from the analysis while p is the p-value. Type refers to the type of trait analysed and finally q-values have been reported.

File Name: Supplementary Data 21
Description: Prioritized genes differentially expressed in balding and non balding dermal papilla cells according to Chew et al 2016. DHT_only denotes differential expression only under DHT stimulation while "Same" both at baseline and after DHT stimulation. Direction refers to badling cells compared to non-balding cells.
